# Structure of a bacterial ribonucleoprotein complex central to the control of cell envelope biogenesis

**DOI:** 10.15252/embj.2022112574

**Published:** 2022-12-12

**Authors:** Md Saiful Islam, Steven W Hardwick, Laura Quell, Svetlana Durica‐Mitic, Dimitri Y Chirgadze, Boris Görke, Ben F Luisi

**Affiliations:** ^1^ Department of Biochemistry University of Cambridge Cambridge UK; ^2^ Department of Microbiology, Immunobiology and Genetics, Max Perutz Labs University of Vienna, Vienna Biocenter (VBC) Vienna Austria

**Keywords:** amino‐sugar regulation, Post‐transcriptional control, RNA chaperone, RNA metabolism, small regulatory RNA, Microbiology, Virology & Host Pathogen Interaction, RNA Biology, Structural Biology

## Abstract

Biogenesis of the essential precursor of the bacterial cell envelope, glucosamine‐6‐phosphate (GlcN6P), is controlled by intricate post‐transcriptional networks mediated by GlmZ, a small regulatory RNA (sRNA). GlmZ stimulates translation of the mRNA encoding GlcN6P synthtase in *Escherichia coli*, but when bound by RapZ protein, the sRNA becomes inactivated through cleavage by the endoribonuclease RNase E. Here, we report the cryoEM structure of the RapZ:GlmZ complex, revealing a complementary match of the RapZ tetrameric quaternary structure to structural repeats in the sRNA. The nucleic acid is contacted by RapZ mostly through a highly conserved domain that shares an evolutionary relationship with phosphofructokinase and suggests links between metabolism and riboregulation. We also present the structure of a precleavage intermediate formed between the binary RapZ:GlmZ complex and RNase E that reveals how GlmZ is presented and recognised by the enzyme. The structures provide a framework for understanding how other encounter complexes might guide recognition and action of endoribonucleases on target transcripts, and how structured substrates in polycistronic precursors may be recognised for processing by RNase E.

## Graphical abstract

The CryoEM structure of an sRNA‐protein complex provides insight into the regulation of bacterial cell wall synthesis.

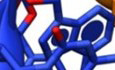

## Introduction

In all known bacteria, the post‐transcriptional control of gene expression is fine‐tuned and integrated into elaborate networks through the actions of numerous small regulatory RNAs (sRNAs). These RNA species can boost or suppress the expression of target mRNAs to which they base‐pair with imperfect complementarity (Wagner & Romby, 2015; Hör *et al*, 2020). sRNA activities are mediated by RNA‐binding proteins that facilitate specific and regulated recognition, and they can guide globally acting ribonucleases to silence specific targets. A few bacterial sRNA‐binding proteins have been studied and structurally elucidated, including the RNA chaperones Hfq, ProQ and CsrA (Holmqvist & Vogel, 2018; Babitzke *et al*, 2019; Quendera *et al*, 2020). However, how such RNA‐chaperones act mechanistically to achieve selective recognition and turnover of the target RNA by the ribonuclease is still unsolved.

A salient example of sRNA‐mediated riboregulation in network control is found in the biogenesis of the bacterial cell envelope (Khan *et al*, 2020). A key component of the cell envelope is a peptidoglycan layer that provides mechanical robustness and cellular integrity. In addition, Gram‐negative bacteria are surrounded by outer membranes containing lipopolysaccharide (LPS), which are recognised by the host innate immune system but also provide protection against many antimicrobials. Biosynthesis of both the peptidoglycan layer and LPS relies on UDP‐GlcNAc, which is produced from the amino‐sugar glucosamine‐6 phosphate (GlcN6P) (Khan *et al*, 2016). GlcN6P can be generated *de novo* by the enzyme glucosamine‐6‐phosphate synthase (GlmS) but may also derive from the recycling of the cell wall and available exogenous amino sugars. When cells grow rapidly or encounter antimicrobials that inhibit GlmS, the ensuing deficiency of GlcN6P is sensed and triggers a boost in the synthesis of GlmS mediated by a post‐transcriptional regulatory network. This feedback pathway enables the supply of the required metabolite to meet the cellular demand (Khan *et al*, 2016).

**Figure 1:**
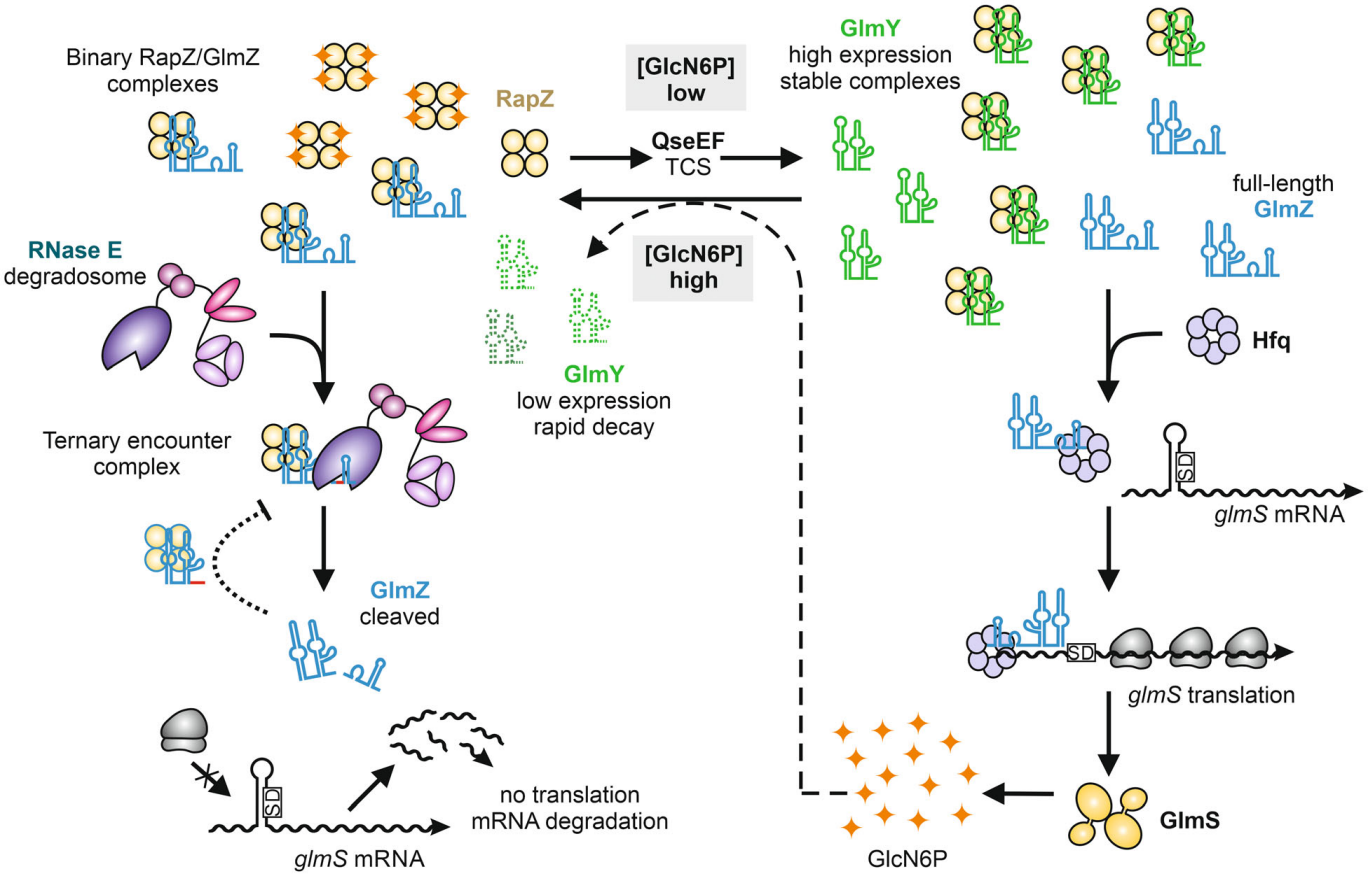
An abbreviated model for the role of RapZ for small RNA‐mediated regulation of GlcN6P synthesis When GlcN6P levels are depleted, RapZ interacts with the two‐component system QseE/QseF, triggering a boost in the expression of the sRNA GlmY, which in turn sequesters RapZ into stable complexes and allows the homologous sRNA GlmZ to interact with Hfq. Binding to Hfq facilitates GlmZ base‐pairing with the *glmS* mRNA, which stimulates translation of the mRNA, elevating GlmS enzyme levels and replenishing GlcN6P (right). When the GlcN6P concentration reaches the threshold, RapZ is released from complexes with GlmY and the sRNA is rapidly degraded. The liberated RapZ binds and presents GlmZ to endoribonuclease RNase E, which cleaves the sRNA in the base‐pairing site, thus stopping stimulation of *glmS* translation and returning enzyme levels to basal (left). The regulatory scheme is likely deeply networked with other cellular pathways through metabolite signalling.

Cellular levels of GlcN6P are sensed by the sRNA‐binding protein RapZ (Khan *et al*, 2020; Fig 1). Low levels of the metabolite favour RapZ interaction with a two‐component signalling system formed by the proteins QseE and QseF, initiating downstream processes that result in activation of the expression of the sRNA GlmY, which in turn is sequestered by RapZ in a stable complex. RapZ can also bind a structural orthologue of GlmY, known as GlmZ (Fig 1), but binding is competitive and high levels of GlmY displace GlmZ. The released GlmZ is captured by the RNA‐chaperone protein Hfq, which protects the sRNA against ribonuclease attack and facilitates its base‐pairing with an Anti‐Shine‐Dalgarno sequence in the *glmS* mRNA encoding the synthase. This interaction boosts the translation of the mRNA, increasing the levels of the enzyme and the metabolite (Kalamorz *et al*, 2007; Reichenbach *et al*, 2008; Urban & Vogel, 2008; Göpel *et al*, 2013, 2016). When GlcN6P levels reach the threshold, GlmY is released by RapZ and degraded, allowing RapZ to bind GlmZ instead (Khan *et al*, 2020). RapZ then presents GlmZ to the endoribonuclease RNase E in a manner that directs cleavage within the base‐pairing site of the sRNA, thereby inactivating it and silencing synthesis of the synthase (Göpel *et al*, 2013; Gonzalez *et al*, 2017; Durica‐Mitic *et al*, 2020; Fig 1). Upon accumulation, processed GlmZ (GlmZ*) binds and sequesters RapZ, thus inhibiting complete GlmZ turnover and ensuring that basal *glmS* expression is buffered against fluctuations in RapZ availability (Durica‐Mitic & Görke, 2019).

**Figure 2:**
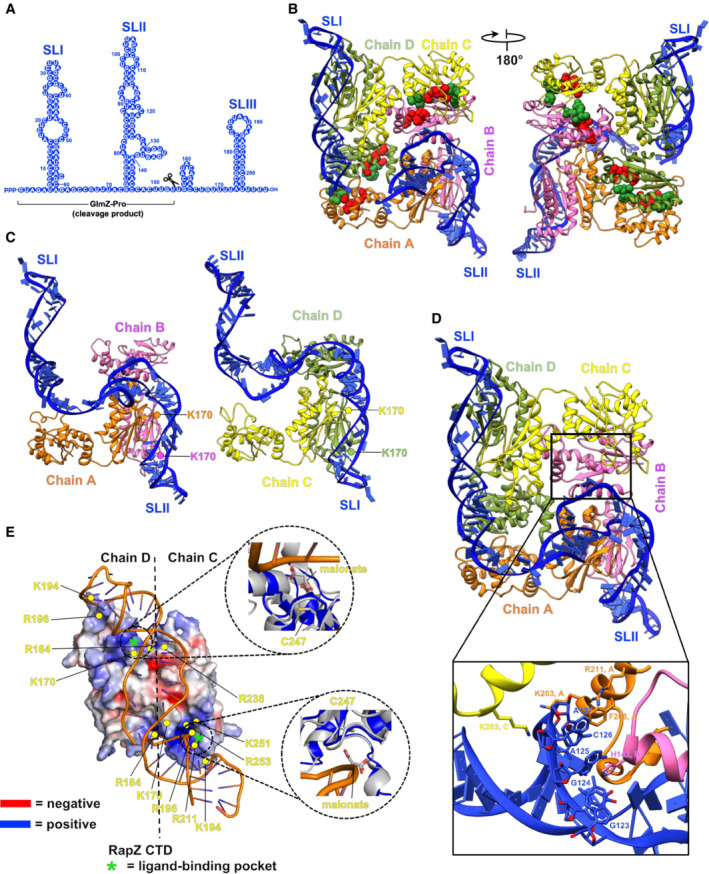
The cryoEM structure of the RapZ:GlmZ binary complex GlmZ secondary structure predicted by ViennaRNA Package 2.0 (Lorenz *et al*, 2011).3D model of the RapZ:GlmZ binary complex. The residues of the Walker A and Walker B motifs are shown in space‐filling representation and coloured red and green, respectively.Similarity of the interactions of RapZ C‐terminal domain (CTD) with SLI and SLII; a side‐by‐side comparison of the interactions of the stem loops with dimer sub‐assemblies of RapZ.Interaction between SLII and residues of RapZ chains A, B and C. K203 in chains A and C (K203^A,C^) interact with the phosphate backbone; F208^A^ interacts with A127^GlmZ^, and H142^B^ interacts with A125^GlmZ^.View down the pseudo two‐fold axis of SLI interacting with a dimer of the RapZ‐CTD. The RNA duplex is underwound, which is apparent from the widened groove as viewed in this perspective. Interaction with GlmZ is achieved by the complementary electrostatic surface of the RapZ structure. The RapZ‐CTD is enriched mostly with basic residues. The insets show overlays revealing that the location of malonate molecules in the crystal structure of apo‐RapZ (PDB:5O5S, Gonzalez *et al*, 2017) occurs at the sites contacting GlmZ in the RapZ:GlmZ binary complex.

RNase E plays a critical role in the complex network. The enzyme is a highly conserved, hydrolytic endoribonuclease encoded by bacteria of diverse families (Mackie, 2013), and it is known to prefer to cleave single‐stranded regions at the phosphate located two nucleotides upstream from uracil (Chao *et al*, 2017). The catalytic activity of RNase E is enhanced by a 5' monophosphate group on certain substrates (Deana *et al*, 2008), but this effect can be by‐passed for some RNA species (Clarke *et al*, 2014), most likely through fold recognition (Bandyra *et al*, 2018; Updegrove *et al*, 2019). One mechanistic puzzle is why RNase E requires RapZ to cleave GlmZ. Clues as to how other RNAs might be recognised by RNase E have been provided by crystal structures of the catalytic domain of the *E. coli* enzyme in apo and RNA‐bound forms, revealing a homotetramer organised as a dimer‐of‐dimers and details of the active site (Appendix Fig S1a; Callaghan *et al*, 2005; Koslover *et al*, 2008; Bandyra *et al*, 2018). Crystallographic data are also available for RapZ and reveal a tetrameric quaternary assembly (Gonzalez *et al*, 2017). The protomers have two domains of roughly equal size: the N‐terminal domain (NTD) bears Walker A and B motifs and resembles an adenylate kinase, while the C‐terminal domain (CTD) bears structural similarity with the sugar‐binding domain of phosphofructokinase. The far C‐terminus of RapZ contains a cluster of positively charged residues, which have been implicated in RNA binding (Göpel *et al*, 2013; Durica‐Mitic *et al*, 2020). The N‐ and C‐terminal domains form separate symmetrical dimers lying at the apexes of the RapZ tetramer in a cyclic organisation (Fig 2B), and the quaternary assembly is maintained by homotypic (NTD‐NTD and CTD‐CTD) and heterotypic interactions (NTD‐CTD) (Gonzalez *et al*, 2017). Interestingly, neither of the isolated domains interact with RNase E (Durica‐Mitic *et al*, 2020), while RapZ‐CTD dimer retains RNA‐binding capacity (Gonzalez *et al*, 2017). Molecular genetics data indicate the requirement of the quaternary structure for stimulation of RNase E cleavage activity *in vitro* and overall regulatory activity *in vivo* (Gonzalez *et al*, 2017; Durica‐Mitic *et al*, 2020). It is likely that the tetrameric organisation is required to present GlmZ for recognition and silencing by RNase E.

While detailed structures are available for apo‐RapZ, there is no structural insight illuminating how the protein recognises GlmZ or GlmY. Secondary structure predictions and structural probing for GlmZ indicate 3 major stem loop (SL) structures (I, II, III) that in principle could provide key recognition features for both RapZ and RNase E (Göpel *et al*, 2013, 2016; Fig 2A). SL III in GlmZ serves as a transcriptional terminator and does not appear critical for directing RNase E cleavage since it can be replaced with noncognate terminators (Göpel *et al*, 2016). RapZ directs RNase E to cleave GlmZ at a site located in the single‐stranded region between SLII and SLIII (Fig 2A). Structural probing experiments did not indicate any structural reorganisation of GlmZ when bound to RapZ (Göpel *et al*, 2013), which suggests that the protein does not simply make the cleavage site accessible for RNase E through induced fit.

Here, we have elucidated the structures of both the binary complex of RapZ:GlmZ and the ternary complex of the RNase E catalytic domain (RNase E‐NTD), RapZ and GlmZ, using electron cryo microscopy (cryoEM). The data reveal how an imperfect structural repeat in the RNA matches the tetrameric quaternary structure of RapZ. The protein largely contacts the RNA through the phosphofructokinase‐like domain, supporting the hypothesis that ancient metabolic enzymes can moonlight or evolve as RNA binders with regulatory consequences. The RNase E‐NTD:RapZ:GlmZ structure elucidates how hydrolytic cleavage of GlmZ occurs by the N‐terminal catalytic domain (NTD) of RNase E through a cooperative mechanism where RapZ plays an intricate role. Our data provide insights into the molecular recognition and the basis for discrimination between structurally similar RNAs through the binding of imperfect RNA duplex repeats and the presentation of a single‐stranded region to the catalytic site of RNase E. Moreover, our models suggest a general RNase E recognition pathway for complex substrates, and how other RNA chaperones such as Hfq might work in an analogous assembly to present base‐paired sRNA/mRNA pairs for cleavage.

## Results

### *In vitro* reconstitution of RapZ:GlmZ and RNase E:RapZ:GlmZ complexes

**Figure EV1:**
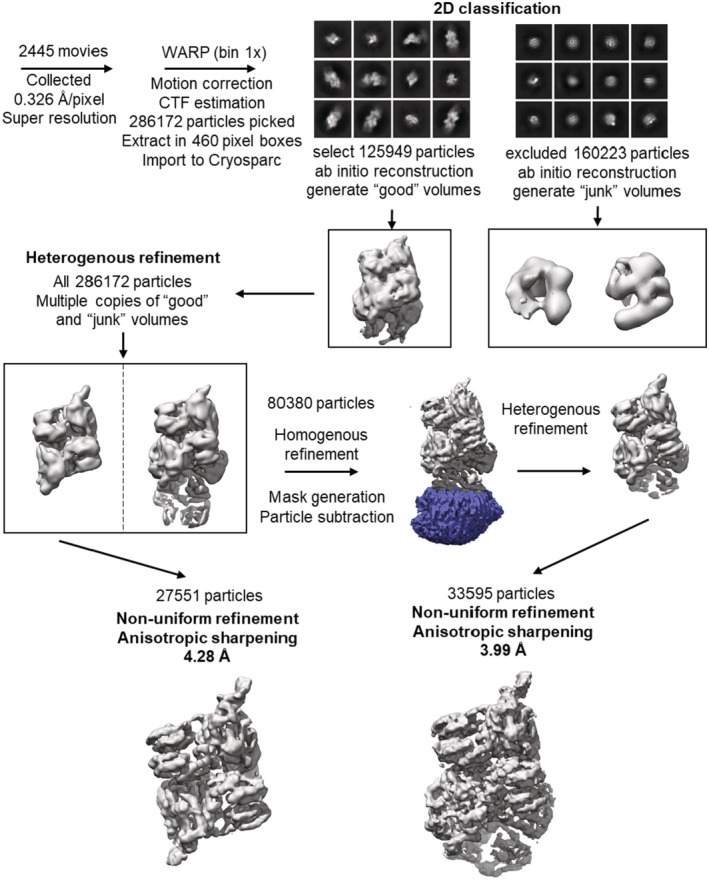
Workflow for cryoEM data collection and processing From the subset of particles giving good volumes, the heterogenous refinement separated the particles into two main groups, corresponding to binary and ternary complexes. Initial grid preparation with Quantifoil and Ultrafoil grids showed aggregation or visible particles (not shown). Better results were obtained with graphene oxide‐covered grids.

**Figure EV2:**
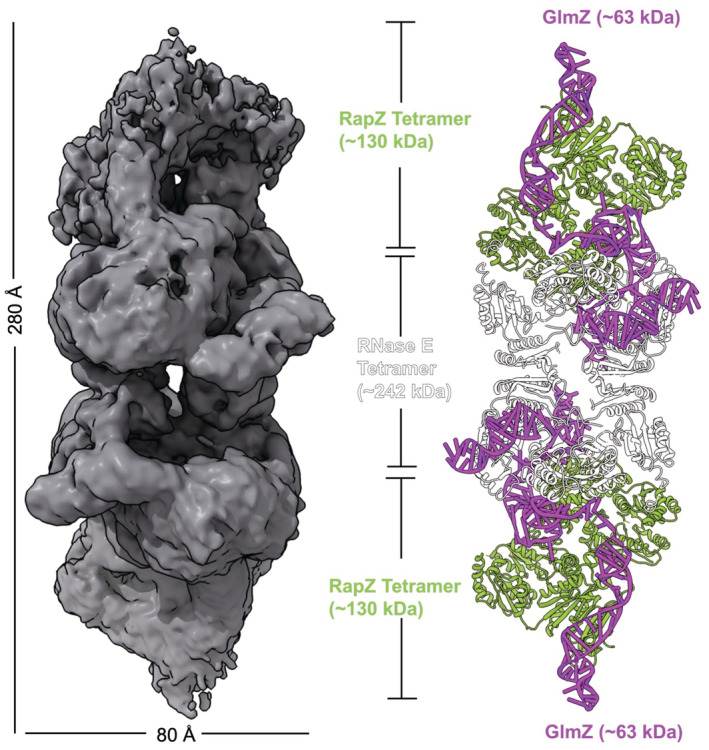
CryoEM model of the ternary RNase E‐NTD:RapZ:GlmZ complex The panel (left) shows the cryoEM map, which is in grey. The model (right) of RNase E‐NTD:RapZ:GlmZ complex comprising one copy of RNase E‐NTD tetramer (white), two copies of RapZ tetramer (olive) and two copies of GlmZ RNA (light purple). Previously reported crystal structures of RapZ (PDB: 5O5O) and RNase E‐NTD (PDB:6G63) and a model of GlmZ RNA generated by ViennaRNA Package 2.0 (Lorenz *et al*, 2011) were used to build the model of the RNase E‐NTD:RapZ:GlmZ complex.

**Figure EV3:**
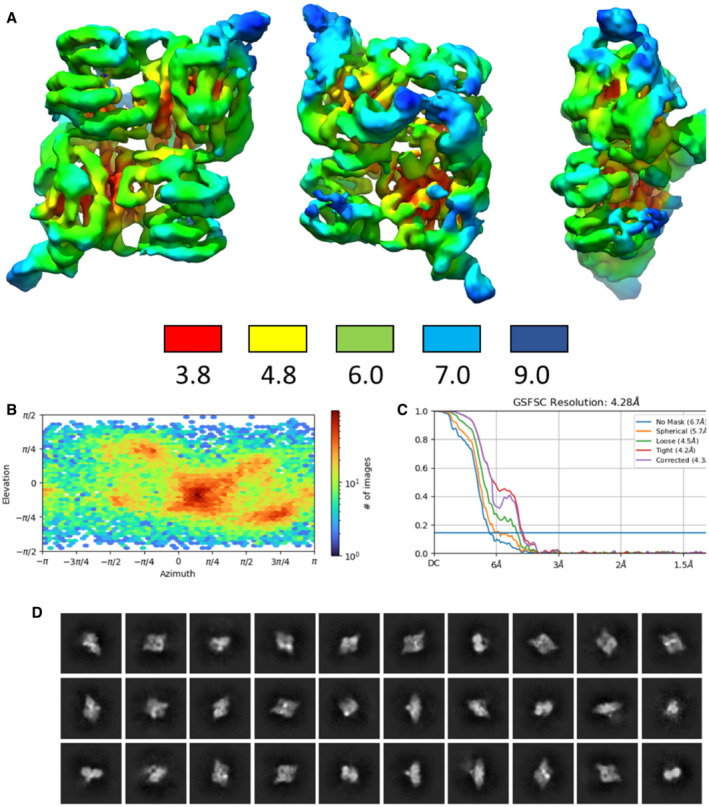
Summary of cryoEM analysis of the RapZ:GlmZ binary complex Three views of the local resolution map of the binary complex calculated in cryoSPARC and coloured by resolution in angstroms according to the key shown below the maps.Angular distribution calculated in cryoSPARC for particle projections contributing to the final map shown as a heat map. The colour coding for the heat map is shown in the bar to the right.Fourier shell correlation (FSC) resolution curves as calculated by cryoSPARC with different masks.2D class images from particles contributing to the final binary RapZ:GlmZ complex cryoEM map.

**Figure EV4:**
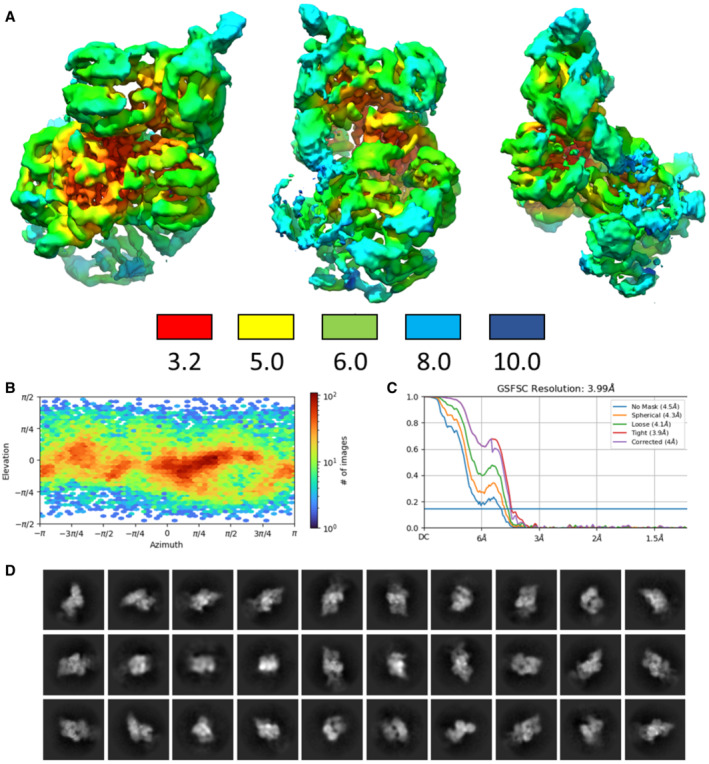
Summary of cryoEM analysis of the RNase E‐NTD:RapZ:GlmZ ternary complex Local resolution map of the ternary complex calculated in cryoSPARC and coloured by resolution in angstroms according to the key below.Angular distribution calculated in cryoSPARC for particle projections contributing to the final map shown as a heat map.FSC resolution curves as calculated by cryoSPARC with different masks.2D class images from particles contributing to the final cryoEM map of ternary complex RNaseE‐NTD:RapZ:GlmZ.

Previous reports have shown that, in the presence of RapZ, the catalytic domain of RNase E (RNase E‐NTD) is sufficient to cleave GlmZ *in vivo* and *in vitro* (Göpel *et al*, 2013; Gonzalez *et al*, 2017; Durica‐Mitic *et al*, 2020). To capture a precleavage Michaelis–Menten state of the catalytic domain, the active site residue aspartate 346 in RNase E‐NTD was replaced with cysteine (Appendix Fig S1a), which changes the metal requirement for ribonuclease activity from Mg^2+^ to Mn^2+^ (Thompson *et al*, 2015). An *in vitro* assay including RapZ confirms that this variant is inactive in the presence of Mg^2+^ but can cleave GlmZ in the presence of Mn^2+^ (Islam *et al*, 2021) (Appendix Fig S1b and c). We purified the recombinant mutant RNase E‐NTD, RapZ and GlmZ and combined these components to reconstitute the ternary ribonucleoprotein complex in buffer without Mn^2+^ but with Mg^2+^to ensure proper folding of GlmZ and to lock the complex in the precleavage encounter state (Appendix Fig S2). Formation of an equilibrium complex containing mutant RNase E‐NTD, RapZ and GlmZ was confirmed by size‐exclusion chromatography (SEC) and mass‐photometry (Appendix Fig S2). Following extensive optimisation, cryoEM datasets were collected for the RNase E‐NTD:RapZ:GlmZ complex on grids coated with graphene oxide (Figs EV1 and EV2). Initial particle classification and map refinement revealed two main subclasses; one featuring the binary complex comprising one copy of GlmZ and one copy of the RapZ tetramer (Fig EV3), and a second class consisting of a ternary complex of RNase E‐NTD:RapZ:GlmZ. Refinement of the ternary complex map revealed compositional heterogeneity; the subclasses have either one or two RapZ:GlmZ associated with the RNase E‐NTD tetramer (Fig EV4). The ‘fundamental unit’, comprising one RNase E‐NTD dimer, one RapZ tetramer and one GlmZ RNA (~ 314 kDa) was refined through masking and particle subtraction (Fig EV4), as will be discussed further below.

### The RapZ:GlmZ binary complex and RNA structure recognition by the phosphofructokinase‐like domain

A map of the binary complex was obtained at 4.4 Å resolution based on Fourier shell correlation (Fig EV3; Table EV1). This map provides an envelope that accommodates well a crystal structure of the RapZ tetramer and confirms the distinctive quaternary structure observed earlier (Gonzalez *et al*, 2017). Remaining density allowed for the building of the first two stem loops of GlmZ RNA (SLI and SLII) (Fig 2A and B). The RNA spans over the surface of the RapZ tetramer, forming interactions that are complementary to the distinctive quaternary structure of the protein (Fig 2B). SLI and SLII make similar contacts to their corresponding protomers in the RapZ tetramer, involving in many instances the same amino acid positions.

**Figure 3:**
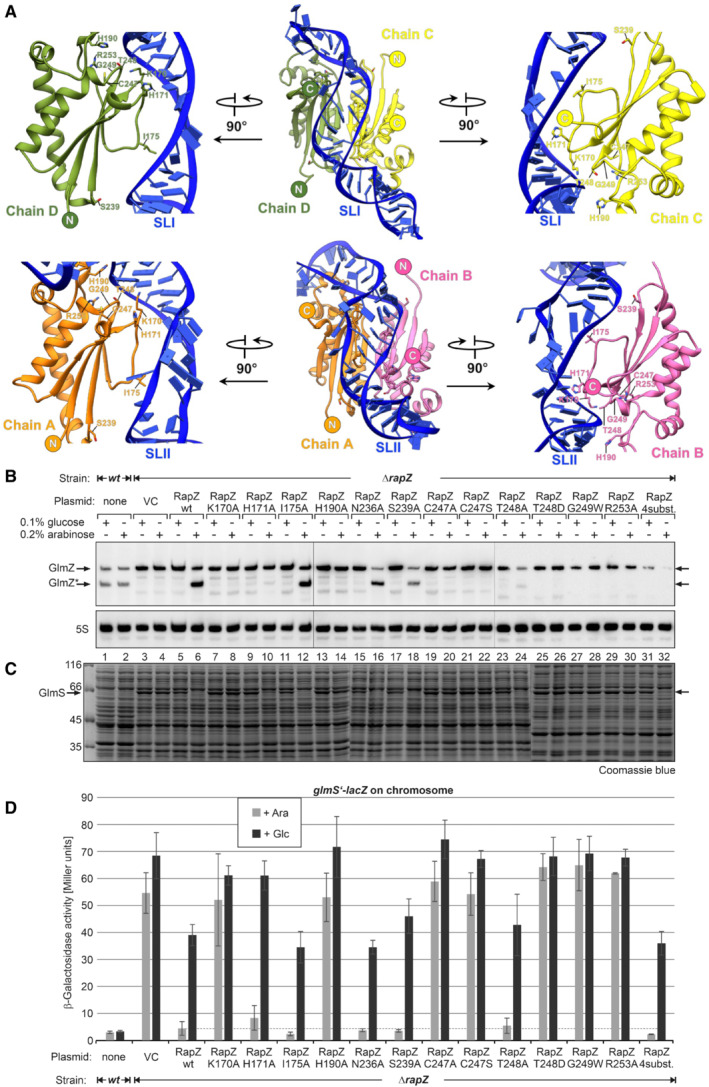
RapZ‐GlmZ interactions support the regulatory activity of RapZ *in vivo* Interaction of the RapZ‐CTD dimers with SLI (top panels) and SLII (bottom panels) of GlmZ. The indicated residues were substituted in RapZ and resulting variants were tested for regulatory activity *in vivo*. The *rapZ* mutants were expressed from plasmids under the control of the *P*_*BAD*_ promoter (induction with arabinose, repression by glucose) and tested for the ability to complement a strain lacking the chromosomal *rapZ* gene in (B–D).Northern blotting experiment detecting GlmZ (top panel) and 5S rRNA (bottom panel; loading control) to assess the degree of GlmZ cleavage triggered by the RapZ variants. Processed GlmZ is indicated with an asterisk.GlmS protein (MW = 66.9 kDa) levels in the same cultures as revealed by SDS–PAGE of total protein extracts followed by Coomassie blue staining.β‐Galactosidase activities produced by the same cultures. Mean values and standard deviations of replicates are shown. Cells carry a *glmS′‐lacZ* fusion on the chromosome, which requires base‐pairing with sRNA GlmZ for high expression. Strains Z8 (*wild type*, lanes/columns 1, 2) and Z28 (*ΔrapZ*, all other lanes/columns) were used. Strain Z28 carried plasmid pBAD33 (lanes/columns 3, 4; empty vector control = VC) or derivatives encoding the indicated RapZ variants (cf. Appendix Table S2 for corresponding plasmids). Source data are available online for this figure.

The RNA stem loops are primarily contacted by the CTD dimers of RapZ; SLI interacts with the CTD dimer formed by chains C and D, whilst SLII interacts with the dimer formed by chains A and B (Fig 2B and C). For both stem loops, the path of the RNA across the CTD dimer has approximately two‐fold symmetry, and the conformation of the duplex changes from A‐form to a highly underwound helix near the dyad symmetry axis (roughly between residue K170 of chains A and B, respectively, Fig 2E). Several basic residues (K170, R184, K194, R196, R211, R238, K251) form patterns of positive charges on the CTD surface providing a favourable electrostatic match to the path of the sRNA (Fig 2E). The phosphate backbones of each of the two stem loops of GlmZ align with corresponding putative ligand‐binding pockets formed by the CTDs of chains A/B and C/D, respectively. These pockets are composed of residues H190, T248, G249, H252 and R253 with C247 being in close vicinity, respectively (Figs 2E and 3A). Sulphate and malonate ions were previously seen at these positions in crystal structures of *apo*‐RapZ (Gonzalez *et al*, 2017). The pocket has shape and electrostatic complementarity to the path of one strand of the A‐form RNA helix involving interactions with nucleotides A125 and A127 in SLII of GlmZ (Fig 2D, lower panel). The groove shape of the A‐form helix is recognised by an exposed loop comprising RapZ residues 190–197. The sulphate‐binding pocket has been proposed to also be the site for the binding of glucosamine‐6‐phosphate (Gonzalez *et al*, 2017), which is supported by recent studies showing the binding of the metabolite to the CTD of RapZ (Khan *et al*, 2020). The Walker A and B motifs located in the N‐terminal domain of RapZ are not positioned to contact the RNA substrate, instead they appear to run along the opposite diagonal of the RapZ tetramer to the RNA (Fig 2B).

The RNase E cleavage site in GlmZ, which resides within the single‐stranded region located between SLII and SLIII, is not resolved in our cryoEM maps and is likely disordered. Thus, while interactions of GlmZ with RapZ present the GlmZ cleavage site to RNase E, it does not appear to preorganise the substrate for enzyme attack. The binary complex is also likely to resemble the postcleavage product, where only the SLI and SLII are bound by RapZ. Thus, processed GlmZ may interact with RapZ through contacts made by SLI and SLII as observed for full‐length GlmZ. This explains earlier observations that the accumulation of processed GlmZ counteracts cleavage through competition for RapZ, thereby providing feedback regulation (Durica‐Mitic & Görke, 2019).

### RapZ:GlmZ contacts in the binary complex are crucial for regulatory activity *in vivo*

The residues of all four sulphate/malonate‐binding pockets are observed in close vicinity to the phosphate backbone of GlmZ. The pockets of chains C and D contact the strands composing the A‐form helix of SLI and the pockets of chains A/B contact SLII of GlmZ (Figs 2E and 3A). Hence, residues of these pockets should be key for the proper orientation of the sRNA on the RapZ tetramer and its correct presentation to RNase E for cleavage. If so, their mutation is expected to interfere with the cleavage of GlmZ by RNase E in the living cell, resulting in the synthesis of GlmS at high levels (Kalamorz *et al*, 2007; Gonzalez *et al*, 2017). To test this, we changed four residues of the sulphate/malonate‐binding pocket in RapZ (H190, T248, G249, R253) with disruptive substitutions. Additionally, we substituted nearby located residues K170, H171, I175, N236, S239 and C247, which might contribute to correct sRNA binding (Fig 3A). The *rapZ* mutants were expressed from plasmids under the control of the arabinose‐inducible *P*_*BAD*_ promoter and analysed for complementation of an *E. coli* strain lacking the chromosomal *rapZ* copy. Western blot analysis confirmed synthesis of the RapZ variants upon induction with arabinose (Appendix Fig S3). Northern blot analysis confirmed that cleavage of GlmZ by RNase E is abolished in the *ΔrapZ* strain carrying the empty vector (VC, Fig 3B). The failure to degrade full‐length GlmZ strongly activates *glmS′* translation, as monitored by an ectopic *glmS′‐lacZ* reporter fusion (Fig 3D), leading to accumulation of GlmS protein visible even in stained total protein extracts (Fig 3C, lanes 3–4). The presence of wild‐type RapZ, either encoded endogenously or expressed from a plasmid, promotes the processing of GlmZ and suppresses *glmS* expression as expected from previous work (Fig 3C and D, lanes 1–2, 6; Durica‐Mitic *et al*, 2020).

Importantly, the substitutions H190A, G249W and R253A—all located in the sulphate/malonate pocket—abolished the ability of RapZ to promote GlmZ cleavage and repress *glmS* (Fig 3B–D). RapZ also lost activity when T248 was replaced with an Asp but still decreased GlmZ levels when this residue was substituted with Ala, supporting our notion that electrostatic complementarity is important for interaction with the negatively charged RNA backbone. This is further supported by the inactivity of the K170A variant (Fig 3B–D), likely reflecting the loss of direct interaction with the nearby located RNA backbone (Figs 2E and 3A). Finally, Ala and Ser substitutions of C247, which is close to the sulphate/malonate pocket (Figs 2E and 3A) abolish RapZ activity. This observation was unexpected for the C247S variant, and it may be the ‐SH group fulfils a specialised catalytic function. From the remaining variants, the Ala substitutions of I175, N236 and S239 had no effect, and the substitution of H171 lowered the repression of *glmS* to some degree (Fig 3B–D).

To address whether the impact on GlmZ processing observed for the RapZ variants is due to a lack of RNA‐binding ability, we performed pull‐down experiments. Here, we carried out StrepTactin affinity chromatography using the Strep‐tagged RapZ variants as bait proteins and analysed the resulting samples for co‐eluting GlmZ RNA via Northern blotting. While wild‐type RapZ is clearly able to bind and co‐elute GlmZ, all tested variants failed to do so (Appendix Fig S4c), suggesting a loss of binding ability. Collectively, the results show that K170, C247 and the residues of the sulphate/malonate pocket are essential for RapZ to promote cleavage of GlmZ *in vivo* and have also a considerable impact on the binding capacity to the sRNA. These observations agree with the roles of these residues for presenting GlmZ correctly to RNase E, as predicted by the structure of the binary complex (Fig 2).

### Architecture of the RNaseE‐NTD:RapZ:GlmZ complex

Extensive rounds of iterative 2D and 3D classifications revealed the presence of multiple states of the RNase E‐NTD:RapZ:GlmZ complex, all containing a single RNase E‐NTD tetramer but with varying occupancies of associated RapZ tetramers (Appendix Fig S5). However, an RNase E‐NTD tetramer bound to one RapZ:GlmZ binary complex was common to all states, and we focussed on this fundamental unit to generate a structural model of the RNase E‐NTD:RapZ:GlmZ ternary complex (Appendix Fig S6).

**Figure 4:**
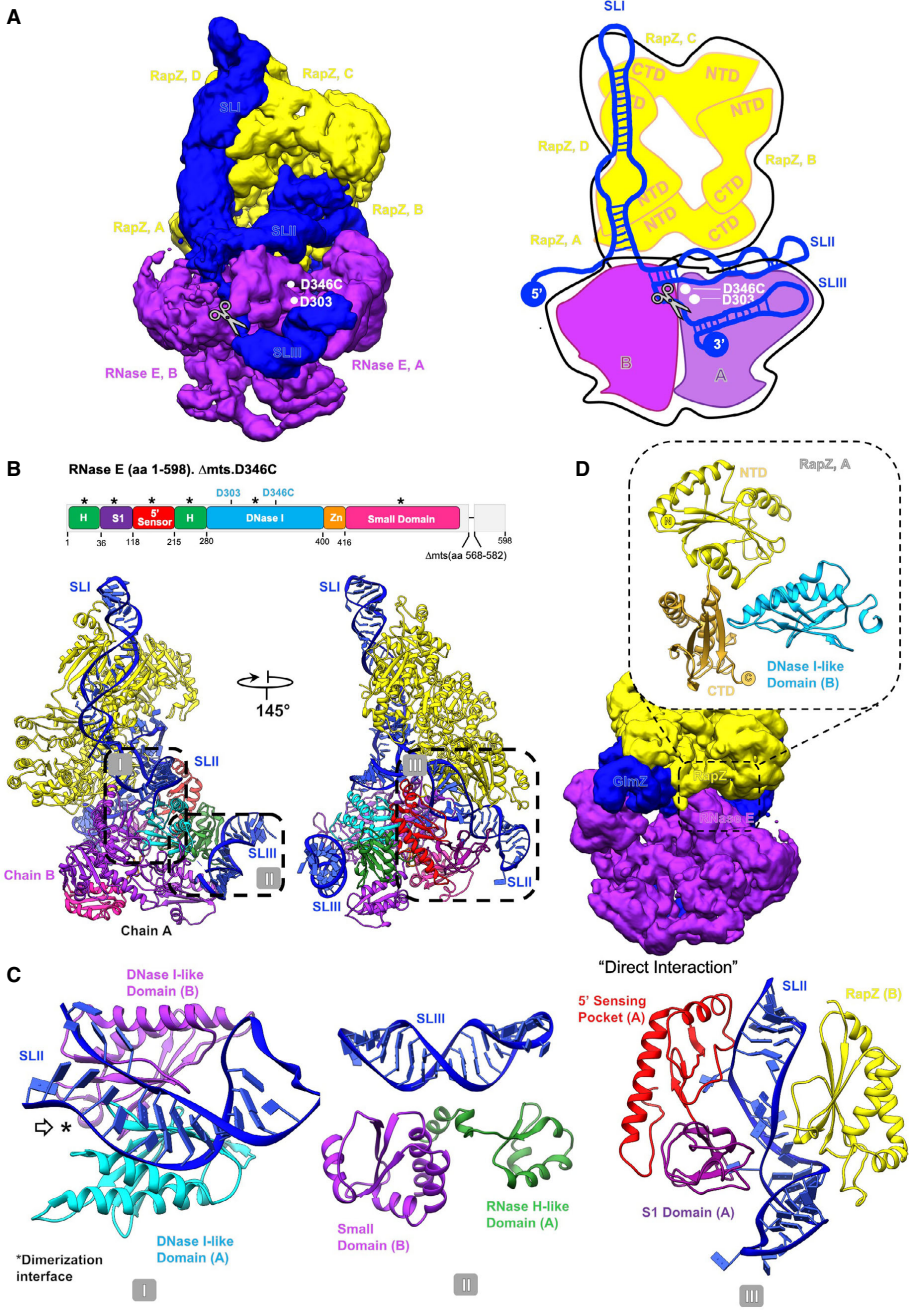
The RNaseE‐NTD:RapZ:GlmZ ternary complex CryoEM map of the ternary complex, representing only the fundamental unit (depicted schematically in the cartoon in the right panel); cf. with Appendix Fig S6, which also provides a perspective of the fundamental unit with respect to the full complex.Model of the fundamental unit with the individual RNase E‐NTD domains coloured according to the domain topology scheme. ‘Asterisks’ on top of the schematic highlight the RNase E domains that interact with the binary RapZ:GlmZ complex. Boxes indicated with roman numerals I, II and III indicate regions shown in the expanded view in (C).Detail views of the interactions between the RNase E‐NTD and the sRNA: (C, left) Interaction of the RNase E DNase I‐like domain with GlmZ‐SLII, mediated via the dimerization interface of two DNase I domains (white arrow and asterisk indicate the dimerization interface) from two nearby RNase E protomers. (C, middle) Interaction of the GlmZ‐SLIII duplex with the RNase H‐like domain of the cleaving protomer and the small domain of the nearby noncleaving protomer. (C, right) Interaction between the 5′‐sensing pocket and S1 domain of the noncleaving protomer and GlmZ‐SLII; this interaction facilitates interaction with the CTD of one of the RapZ protomers (chain B) using GlmZ as a bridge, further promoting the formation of the overall complex.Illustration of the direct interaction of RNase E with RapZ through a confined contact region.

From our cryoEM map, the quaternary organisation of the complex can be defined with confidence, as well as the general path of the RNA over the surface of the two proteins (Figs 4A and EV2). The binary complex of RapZ:GlmZ and the crystal structure of the tetrameric NTD of RNase E both fit well into the experimental map with small domain movements. Thus, the interaction of RapZ:GlmZ with RNase E does not involve conformational rearrangement of either RapZ or GlmZ. Previously published crystal and cryoEM structures of RNase E‐NTD have revealed many degrees of freedom at both the quaternary and tertiary levels, enabling flexible accommodation of substrates (Callaghan *et al*, 2005; Koslover *et al*, 2008; Bandyra *et al*, 2012, 2018; Dendooven *et al*, 2022). It may therefore not be surprising that in the ternary complex structure the 5′‐sensor/S1 domains of the RNase E‐NTD have adopted an opened conformation that can accommodate the binary complex—a configuration not previously observed but which may account for the capacity of RNase E to be guided to preferred cleavage sites by RNA structure (Appendix Fig S7).

### Recognition of RNA by RNase E catalytic domain ternary complex

The ternary complex assembly appears to be stabilised predominantly through mutual binding of the RNase E‐NTD and RapZ to GlmZ RNA (Fig 4). In particular, SLII of GlmZ, which was reported to be crucial for RNase E‐mediated cleavage (Göpel *et al*, 2016), appears to be a shared binding site for both RNase E and RapZ (Fig 4B–D). To gain insight into these interactions, we used the adenylate cyclase‐based bacterial two‐hybrid assay (BACTH; Karimova *et al*, 1998). We reasoned that the substitution of residues in RapZ that is required to bind SLII in a correct manner, should also affect the mutual contacts made by RNase E and thereby RapZ‐RNase E interaction as measurable by BACTH in the *E. coli* cell (Durica‐Mitic *et al*, 2020). Indeed, several substitutions including those located within the sulphate/malonate pocket decreased interaction fidelity significantly, with the H190A substitution yielding the strongest effect (Appendix Fig S4). However, the removal of individual RapZ/GlmZ contacts is not sufficient to disrupt the ternary complex completely, and a significant impact on the BACTH signal is seen only with the combination of several substitutions (see Appendix Fig 4). These results support the idea that RapZ must present GlmZ correctly to facilitate mutual contacts by RNase E. On the other hand, RNase E‐NTD variants carrying multiple amino acid exchanges (R141A/R142A/R169A, R357A/R364A and K106A/R109A) in residues that are proposed to be in direct contact with GlmZ, show no decrease in interaction in BACTH assays (Appendix Fig S4b), suggesting that this region makes weak contributions to the assembly formation or that remaining interactions compensate.

The predicted structure of SLIII fits well into the density present on the surface of the RNase E small domain and RNase H fold (Fig 4C; Appendix Fig S11). This binding site agrees with the earlier findings from an X‐ray crystallographic structure of the RNase E‐NTD in complex with the sRNA RprA, which indicated stem loop engagement on that surface (Bandyra *et al*, 2018). The small domain of RNase E is a divergent member of the KH domain family (Pereira & Lupas, 2018) and has a distinctive mode in RNA binding (Bandyra *et al*, 2018).

In a subset of particles, density continues from the base of the GlmZ‐SLIII structure in the direction of the active site in the DNase I sub‐domain of RNase E. The location of the RNA in the active site is consistent with the position of the cleavage site in the single‐stranded region between SLII and SLIII in GlmZ (Göpel *et al*, 2016; Figs 2 and 4). It also agrees with the polarity of the phosphate backbone in the active site required to achieve the necessary geometry for cleavage (Callaghan *et al*, 2005). The S1 domain of RNase E encages the RNA in the active site, in agreement with earlier crystallographic studies (Callaghan *et al*, 2005). The 5′ sensor‐S1 domain is comparatively disordered in relation to other regions within the map, and there is a gap in modelling the RNA in this region.

Continuing to follow along the RNA in the 3′ to 5′ direction, there is a clear density for SLII that engages the exposed surface of the DNase I domains from two nearby RNase E protomers (Fig 4C, left panel). This is the first observation of a duplex RNA interaction with this surface of RNase E; notably, the SLII seems to interact via the dimerization interface of two DNase I domains. The duplex RNA continues and makes interactions with the CTD of the RapZ chain A, where it changes direction and then continues over the surface of the CTD of the RapZ chain B (Fig 4A). The duplex region contacted by RapZ chain A is also contacted by the 5′ sensor domain/S1 domain of RNase E, also revealing for the first time the interactions of this domain with a duplex RNA (Fig 4C, right panel; Appendix Fig S7). The interactions of the SLII with RNase E are predicted to be the key determinant for the recognition of GlmZ as a substrate. Indeed, GlmY becomes a substrate for RapZ/RNase E *in vitro* if its SLII is replaced with that from GlmZ (Göpel *et al*, 2016). The base of SLII is near the active site, where the duplex region of SLI begins to continue over the surface of RapZ and interacts with the CTD of the RapZ chains C and D.

While the ternary complex of RNase E, GlmZ and RapZ is predominantly held together by protein‐RNA interactions, there are also a few protein–protein interactions. Notably, the DNase I domain of RNase E chain B directly interacts with chain A of the RapZ tetramer (Fig 4D). The model reveals a small direct contact surface between RapZ and RNase E, formed by nestling of the DNase I domain between the N‐ and C‐terminal domains of a proximal RapZ protomer. Here, RNase E segment 311–316 interacts with the surface of RapZ that includes residues Q273, N271, Y240 and T161. This complex interaction surface is consistent with results from mutagenesis screening performed in combination with bacterial two‐hybrid assays (Durica‐Mitic *et al*, 2020). The latter analysis showed that the removal of a few residues from the RapZ C‐terminus abolished interaction with RNase E and indicates a critical role of residue L279 for direct interaction with RNase E. These observations are rationalised by the cryoEM model indicating that the RapZ C‐terminus including residue L279 may support the correct presentation of the strand that carries the contacting residues Q273 and N271.

BACTH assays addressing a RapZ variant carrying Alanine substitutions in the residues Q273, N271, Y240 and T161 (also labelled RapZ 4 substitutions: RapZ 4 subst.) show a 2‐fold decrease in interaction with RNase E (Appendix Fig S4a). This suggests that residues on the RapZ surface, which are proposed to be in direct protein–protein contact with RNase E, contribute to the complex formation and/or stabilisation. Already, previous data show weakened *in vitro* interaction of the catalytic domain of RNase E with RapZ in the absence of RNA (Durica‐Mitic *et al*, 2020). Complementation analysis of this RapZ variant shows that it behaves comparably to RapZ wild type in the *glmS‐lacZ* reporter assay (Fig 3D, lanes 31–32). Interestingly, processing by the RapZ variant carrying these 4 substitutions is reminiscent of RapZ variant T248A (Fig 3B and C, compare lanes 23–24 to 31–32), suggesting that these four exchanges have an impact on GlmZ* stability.

## Discussion

The orderly biogenesis of the bacterial cell envelope during growth and stress response is supported by a responsive network that helps to match the demand for the required building blocks. One step in the network is the activation of GlmS by GlmZ, and loss of repression by removal of the seed pairing region from the regulatory RNA. In Gram‐negative Enterobacteria, the adaptor protein RapZ facilitates the removal of the seed by RNase E. The structural data presented here reveal that RapZ presents the sRNA in a manner that aligns its single‐stranded region comprising the cleavage site into the RNase E active centre and that portions of GlmZ near the intersection of SLI and SLII interact with RNase E to preorganise the channel that provides access to the active site (Fig 4A).

**Figure 5:**
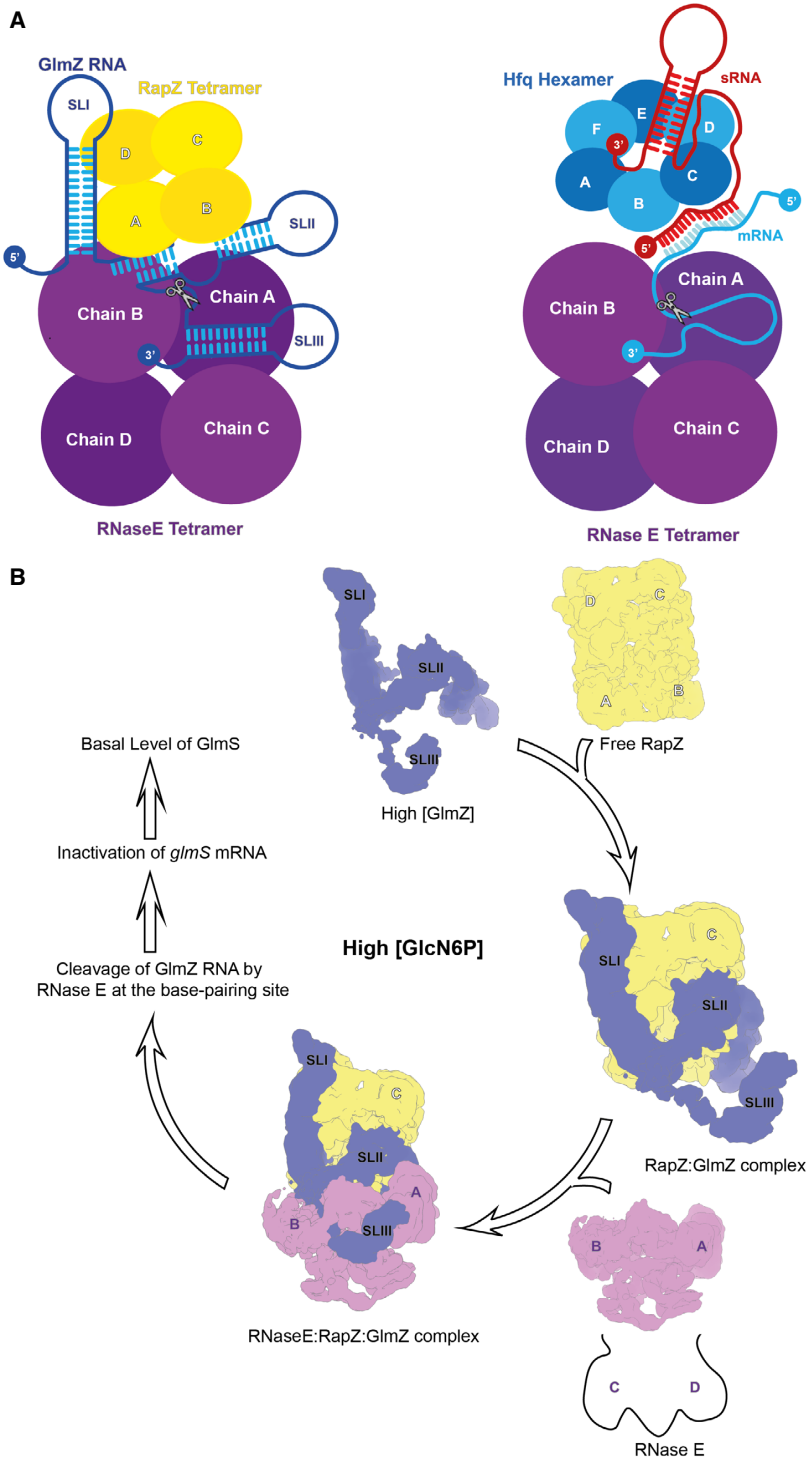
Proposed models for the presentation of RNA‐duplexes by RNA chaperones to RNase E The organisation of the RNaseE‐NTD:RapZ:GlmZ complex (left) suggests an analogous mode for the presentation of complex double‐stranded RNA substrates by other RNA‐chaperones such as Hfq (right). A hypothetical encounter complex is shown illustrating how Hfq may deliver base‐paired small RNAs (red) and RNA targets (blue) to RNase E for cleavage, potentially with 5' end group sensing (Bandyra *et al*, 2012). An mRNA is indicated in the cartoon, but targets could also include noncoding RNAs such as GcvB. The c‐terminal portion of RNase E outside of the catalytic domain is likely to facilitate the capture and presentation of the RNA‐chaperone complexes (Ali & Gowrishankar, 2020).Model illustrating the mechanism of GlmZ cleavage according to findings from current work. As a prerequisite for cleavage, GlmZ must be bound by the tetrameric adapter protein RapZ. Binding occurs through the recognition of SLI and SLII in GlmZ by the CTD dimers of RapZ, whereas SLIII has no role. This mode of recognition explains earlier observations that the processed variants of GlmY and GlmZ carrying SLI and SLII only, compete successfully with full‐length GlmZ for binding RapZ (Göpel *et al*, 2013; Durica‐Mitic & Görke, 2019). Correct presentation of GlmZ by RapZ allows the joining of RNase E to form the ternary encounter complex, which is stabilised through mutual interaction of the proteins with SLII of GlmZ, binding of the GlmZ‐SLIII to the RNase H‐like/small domain surface on RNase E and direct RapZ‐RNase E contacts. This assembly positions the single‐stranded region of GlmZ in the RNase E active site, allowing for cleavage. The resulting GlmZ molecule lacks the base‐pairing site and is not capable to activate *glmS* translation.

We observe that RapZ interacts with SLI and SLII of GlmZ, mainly through a dimer of its C‐terminal domain (CTD). Each CTD dimer within the RapZ tetramer binds one stem loop, which is achieved through complementarity in shape and electrostatic charge to the phosphodiester backbone of the sRNA (Figs 2 and 3A). This arrangement also accounts for the observation that a CTD dimer on its own is sufficient to bind the sRNAs with an affinity comparable to that of the full‐length protein (Gonzalez *et al*, 2017; Durica‐Mitic *et al*, 2020). However, the CTD dimer is not sufficient to mediate cleavage of GlmZ by RNase E, and the structure of the RapZ tetramer accounts for this observation. Both the N‐ and C‐terminal domains are required to form the quaternary structure that is needed to bind both RNA stem loops and present the single‐stranded cleavage site appropriately to RNase E. The kinase‐like N‐terminal domain of RapZ (NTD) makes only a few interactions with the RNA, and the path of the RNA does not encounter the Walker A or B motifs (Fig 2B). Perhaps this domain could act as an allosteric switch, whereby the binding of a ligand, yet unknown, triggers quaternary structural changes that affect RapZ functions. Interestingly, YvcJ, the homologue of RapZ in *Bacillus subtilis*, was recently shown to bind UDP‐GlcNAc and a mutation in the Walker A motif interferes with binding (Foulquier *et al*, 2020). Preliminary data indicate that RapZ is likewise capable to bind UDP‐GlcNAc.

Several positively charged residues in the CTD provide electrostatic complementarity to the path of the sRNA (Fig 2E) and residues from putative metabolite pockets make contact with both stem‐loops of GlmZ (Figs 2 and 3A). The conservation of the residues contacting the RNA and putative ligand implicates their functional importance (Appendix Fig S8). Substitution of these residues in RapZ abrogates cleavage of GlmZ in the cell (Fig 3B), confirming their importance for presenting GlmZ correctly to RNase E. Some of the mutations decrease RapZ/RNase E interaction fidelity concomitantly (Appendix Fig S4a), suggesting that correct binding of the sRNA by RapZ is a prerequisite for subsequent recognition by RNase E to form the ternary cleavage complex. However, none of the tested RNase E variants that are predicted to weaken interaction with SLII of GlmZ show a significant difference in BACTH assays for interaction with RapZ when compared to RNase E wild type (Appendix Fig S4b), suggesting that the direct interaction surface of RNase E and GlmZ may not be critical for the ternary assembly. The far C‐terminal tail of RapZ is enriched with basic residues and is known to also contribute to RNA binding (Göpel *et al*, 2013; Durica‐Mitic *et al*, 2020). The cryoEM maps of the binary RapZ:GlmZ complex indicate that this region comprising residues 281–284 is generally disordered. Nonetheless, in all four RapZ subunits the far C‐terminus is near GlmZ, and likely forms distributed interactions with the phosphodiester backbone. In conclusion, the structural features revealed here perfectly rationalise the findings of earlier studies indicating that the RapZ‐CTD binds the sRNA, while the tetrameric structure is indispensable (Gonzalez *et al*, 2017; Durica‐Mitic *et al*, 2020) because it engages two CTD dimers to ensure binding of both SLs, which allows GlmZ to be presented to RNase E.

Inside the cell, cleavage of GlmZ by RNase E is counteracted by the homologous decoy sRNA GlmY, which accumulates when GlcN6P synthesis is required (Fig 1; Göpel *et al*, 2013; Khan *et al*, 2020). Based on the predicted fold, GlmY is likely to form a complex with RapZ that is structurally similar to the RapZ:GlmZ binary complex. Notably, the species accumulating *in vivo* refers to a processed GlmY variant consisting only of SLI and SLII (Reichenbach *et al*, 2008; Urban & Vogel, 2008). SLIII does not contribute to the recognition of the sRNA by RapZ (Fig 2), which explains why short GlmY is nonetheless capable to bind and sequester RapZ efficiently. The model also explains why the postcleavage product, *i.e*. GlmZ lacking SLIII, can still inhibit GlmZ cleavage in a negative feedback loop when accumulating above a threshold (Durica‐Mitic & Görke, 2019). RapZ could in principle bind many RNA species in the cell, but analysis of RNA copurifying with RapZ in pull‐down experiments identified only GlmY and GlmZ as substrates under the conditions employed (Göpel *et al*, 2013). This high specificity likely arises from the requirement for binding two distinct RNA stem loops through a unique pattern of complementary shape and charge in RapZ (Fig 2).

*In vivo*, the adapter function of RapZ is restricted to presenting GlmZ to RNase E for cleavage, at least under the growth conditions analysed so far (Durica‐Mitic *et al*, 2020). This specificity is not simply a consequence of the RNA‐binding specificity of RapZ. For instance, although full‐length GlmY is readily bound by RapZ, it is not cleaved in the GlmY:RapZ binary complex by RNase E in cleavage assays *in vitro* (Göpel *et al*, 2016). However, GlmY becomes cleavable when its SLII is swapped for its counterpart from GlmZ, suggesting that this structure is decisive for recognition by RNase E. SLII of GlmZ contains unique features such as the lateral bulge in the SLII (Fig 2A) that is part of an underwound helix contacted by both RapZ and RNase E in the encounter complex (Figs 2D and 4B). The somewhat different SLII of GlmY is likely unable to mediate this mutual binding to RapZ and to the 5′ sensor‐S1 domain of RNase E. Additionally, the single‐stranded region following SLII requires alignment in correct polarity with the catalytic site of RNase E to get cleaved (Fig 4A and B). This task could be facilitated by the downstream terminator stem loop structure (i.e. SLIII) that engages the RNase H/KH‐like small domain in RNase E. This recognition may be guided by RNA secondary structure and less by sequence, considering that RNase E binds the phosphodiester backbone of an A‐form helix segment in SLIII (Fig 4C, middle panel). Previous structural studies on a distinct sRNA/RNase E complex also indicated stem loop engagement on that surface (Bandyra *et al*, 2018) and molecular biology studies showed that SLIII of GlmZ can be replaced with noncognate terminators without affecting cleavage (Göpel *et al*, 2016). Therefore, it appears reasonable that the RNase H/KH‐like small domain provides a general RNA docking site that could also play a role in the formation of encounter complexes with other RNA chaperones (see below). Finally, the mode of recognition of GlmZ by RapZ and RNase E suggests key features to engineer cleavage sites that could be used to trigger *in vivo* genesis of defined RNA species from precursors (Göpel *et al*, 2016).

Interestingly, the phosphate groups of the RNA backbone occupy positions in RapZ that were previously observed to bind sulphate or malonate ions in the crystal structure of apo‐RapZ, suggesting that this pocket could be the binding site for a charged metabolite such as GlcN6P (Figs 2E and 3A; (Gonzalez *et al*, 2017)). GlcN6P is known to bind to the RapZ‐CTD at a yet uncharacterized site, thereby interfering with sRNA binding and stimulation of QseE/QseF activity (Khan *et al*, 2020). Strikingly, the structure of the binary complex predicts mutually exclusive access of the metabolite and the sRNA to the sulphate/malonate pocket, reinforcing the idea that GlcN6P binds here. Our crystal structure of the CTD in the presence of GlcN6P indicates density in this pocket that is likely a chemically transformed product of the metabolite. Such activity might account for nuanced roles of RapZ in metabolite sensing (Khan & Görke, 2020).

It has been noted earlier that the fold of the C‐terminal domain of RapZ closely resembles the fructose‐6‐phosphate binding domain of the metabolic enzyme phosphofructokinase (Gonzalez *et al*, 2017, Fig 2B). Metabolic enzymes have been identified in moonlighting functions as RNA‐binding proteins with roles in post‐transcriptional regulation (Beckmann *et al*, 2015; Marondedze *et al*, 2016). Only recently, the glycolytic enzyme enolase was shown to be directly regulated by RNA in mammalian cells (Huppertz *et al*, 2022). Our observations hint as to how these enzymes might recruit RNA species through the reallocation of metabolite‐binding sites or be repurposed during evolution for this role, adding to the discussion of possible relationships between metabolic enzymes and RNA‐binding proteins (Hentze *et al*, 2018; Balcerak *et al*, 2019; Corley *et al*, 2020).

For the ternary RNaseE‐NTD:RapZ:GlmZ complex, the predominant stoichiometry we observed was one RNase E‐NTD tetramer, two RapZ tetramers and two GlmZ molecules. However, the binding of the RapZ:GlmZ binary assemblies to the RNase E‐NTD can occur in *cis* or *trans* conformations (with the binary complexes engaging on the same or opposite faces of the RNase E‐NTD) (Appendix Fig S5). These higher‐order complexes may result from the saturation of possible binding sites and are not anticipated to be required mechanistically. However, they do illustrate the flexibility of the RNase E tetramer and the potential for the ribonuclease to undergo conformational adaptation to accommodate larger substrates through multiple contacts mediated by distant sites. This mode of binding could play a role in the capacity of RNase E to migrate on long RNA substrates (Richards & Belasco, 2019, 2021).

The structures studied here may also provide a clue as to the mechanism for other effector complexes known to engage RNase E. For instance, the regulatory protein CsrD, which is required for specific turnover of the sRNAs CsrB and CsrC by RNase E (Vakulskas *et al*, 2016; Potts *et al*, 2018), may present those RNAs for structure‐based recognition in analogy to the RapZ/GlmZ complex studied here. A similar scenario could also apply to recently identified RNA‐binding proteins in nonmodel bacteria such as CcaF1, which modulates the degradation of selected transcripts by RNase E in *Rhodobacter sphaeroides* (Grützner *et al*, 2021), or to the emerging class of KH proteins that may act as sRNA chaperones in Gram‐positive bacteria (Olejniczak *et al*, 2021) and might present RNA to other classes of ribonuclease such as RNase Y. We hypothesise that the global RNA‐chaperone Hfq may form encounter complexes analogous to RapZ to facilitate degradation of base‐paired sRNA/target RNAs by RNase E (Fig 5A, right panel). In this model, the sRNA/target duplex would be recognised by both, Hfq and RNase E, reminiscent of the role of GlmZ‐SLII in the current encounter complex. The sRNA 3′ poly (U) end may stay bound on Hfq while the newly identified duplex binding site on RNase E could bind the structured target RNA to accommodate the cleavable site in the catalytic domain (Fig 2B). The new duplex binding region in RNase E may even have general implications for how binding of other structured RNA substrates may occur. For instance, the processing of polycistronic tRNAs may involve capture complexes in which a duplex region of the tRNA is engaged on RNase E to generate a defined cleavage site (Kime *et al*, 2014). The 3′ located tRNA may be docked onto the RNase H/small domain surface while engaging the preceding tRNA copy on the exposed S1/5′sensor domain. The results presented here provide insight into the mechanism of substrate preferences for RNase E and into the role of RNA chaperones to guide the activity of this key enzyme of RNA metabolism.

## Materials and Methods

### Construction of rapZ and RNase E mutants

Single codon exchanges were introduced into *rapZ* using the combined chain reaction (CCR) method (Bi & Stambrook, 1998), and resulting mutants were placed on plasmid pBAD33 under the control of the arabinose‐inducible *P*_*BAD*_ promoter. Briefly, 5′‐phosphorylated oligonucleotides carrying the desired nucleotide exchanges were incorporated into *rapZ* by thermostable ampligase (Biozym Scientific GmbH) during amplification by PCR using the forward/reverse primers BG1049/BG397. DNA fragments were digested and inserted between SacI/XbaI restriction sites on plasmid pBAD33. Plasmid constructs for the bacterial adenylate cyclase‐based two‐hybrid assay (BACTH) were obtained by CCR reactions using the forward/reverse primers BG637/BG639. DNA fragments were cloned between the XbaI‐KpnI sites on plasmid pKT25 to generate corresponding *T25‐rapZ* fusion genes. Plasmids used in ligand fishing experiments using StrepTactin affinity chromatography were constructed by CCR and cloning of resulting mutant *rapZ* genes into pBGG237 (Luttmann *et al*, 2012) between NheI‐XbaI sites using the forward/reverse primers BG1015/BG397. In case multiple codon exchanges were required, MMRs (multiple mutation reactions; Hames *et al*, 2005) were performed using multiple 5′‐phosphorylated oligonucleotides for mutagenesis. Alternatively, a parental plasmid carrying a subset of the desired exchanges was used as a template for an additional round of CCR. In several cases, established *rapZ* mutants were moved between different vector backgrounds via subcloning. Plasmids and oligonucleotides used in this study are listed in Appendix Tables S1 and S2, respectively. Mutations were verified by DNA sequencing.

### Analysis of the regulatory activity of RapZ variants and their interaction with RNase E *in vivo*

Plasmid pBAD33 and its various derivatives encoding the RapZ variants were introduced into the *ΔrapZ* mutant strain Z28, respectively, and resulting transformants were grown overnight in LB containing 15 μg/ml chloramphenicol and supplemented either with 0.1% glucose or with 0.2% l‐arabinose. For comparison, the isogenic *wild‐type* strain Z8 (*rapZ* ^+^) was included, but chloramphenicol selection was omitted in this case. On the next day, cells were inoculated to an OD_600_ = 0.1 into fresh medium and grown until cultures reached an OD_600_ ~ 0.5–0.8. Subsequently, samples were harvested for isolation of total RNA, total protein and determination of β‐galactosidase activities, respectively. β‐Galactosidase activities were measured as described (Miller, 1972). Presented values represent the average of at least three measurements using at least two independently transformed cell lines. For sodium dodecyl sulphate‐polyacrylamide gel electrophoresis (SDS–PAGE) analysis, cells of each culture corresponding to 0.05 OD_600_ units were dissolved in SDS sample buffer and separated on 10% SDS polyacrylamide gels, which were subsequently stained with Coomassie brilliant blue R‐250 or subjected to Western blotting using a polyclonal RapZ antiserum (Durica‐Mitic & Görke, 2019). Extraction of total RNA and Northern blotting was performed as described previously (Durica‐Mitic *et al*, 2020), unless 2.5 μg total RNA of each sample was separated on denaturing gels containing 7 M urea, 8% acrylamide and 1× TBE. RNase E/RapZ interactions were analysed using the bacterial adenylate cyclase‐based two‐hybrid system (BACTH) according to an established protocol (Durica‐Mitic *et al*, 2020). Briefly, transformants of *E. coli* reporter strain BTH101 carrying the desired combinations of pUT18C‐ and pKT25‐type plasmids were grown overnight to early stationary phase in LB (100 μg/ml ampicillin, 30 μg/ml kanamycin), 1 mM isopropyl‐β‐thiogalactopyranoside (IPTG) and the β‐galactosidase activities were determined.

### Evaluating RapZ interactions with GlmZ using StrepTactin affinity chromatography

Experiments addressing the interaction of Strep‐tagged RapZ variants with sRNA GlmZ were carried out as previously described (Göpel *et al*, 2013; Durica‐Mitic *et al*, 2020). Briefly, Strep‐tagged variants of RapZ were overproduced in strain Z903, which lacks endogenously encoded *rapZ*, and cells were grown and harvested as described previously (Durica‐Mitic *et al*, 2020). Cell lysis was carried out using a one‐shot cell disruptor (1.3 kbar, 1 pass, Constant Systems Ltd.). Eluates derived from affinity chromatography were analysed for protein content by SDS–PAGE/Coomassie blue staining and protein concentrations were measured using Bradford assay. One‐quarter of the eluates was used for RNA extraction as described previously (Göpel *et al*, 2013) and isolated RNAs (normalised to protein content of the elution fraction) were used for northern blotting to detect GlmZ and 5S RNAs.

### RNase E‐NTD expression and purification

Purification of RNase E encompassing residues 1 to 598 (NTD) without the membrane targeting sequences (∆568–582) and with the active site residue Asp^346^ mutated to cysteine (D346C), wild‐type RNase E‐NTD (aa 1–529) followed protocols for shorter versions of the catalytic domain (Callaghan *et al*, 2005; Bandyra *et al*, 2018). *Escherichia coli* strain BL21(DE3) was transfected with pET16 expression vector overproducing RNase E variants with an *N*‐terminal his_6_‐tag (kindly provided by A.J. Carpousis). Cultures were grown in 2xTY media supplemented with 100 μg/ml carbenicillin at 37°C, using dimpled flasks for optimal aeration, in an orbital shaker set at 160 rpm. The culture was induced between 0.5 and 0.6 OD_600_ by adding 1 mM IPTG and harvested after 3 h of incubation by centrifugation at 5,020 *g* and 4°C for 30 min. Cell pellets were stored as a suspension in nickel‐column buffer A (20 mM Tris pH 7.9, 500 mM NaCl, 5 mM imidazole, 1 mM MgCl_2_) at −80°C until further use. Once thawed, the cell‐culture suspension was supplemented with DNase I and EDTA‐free protease inhibitor cocktail tablet (Roche) and lysed by passing through an EmulsiFlex‐05 cell disruptor (Avestin) for 2–3 times at 10–15 kbar pressure. The lysate was clarified by centrifugation at 35,000 *g* for 30 min at 4°C and the supernatant was passed through a 0.45 μ membrane filter before loading onto a pre‐equilibrated HiTrap immobilised metal ion affinity (IMAC) column (HiTrap IMAC FF, GE Healthcare). The column was washed extensively with wash buffer (20 mM Tris pH 7.9, 500 mM NaCl, 100 mM imidazole, 1 mM MgCl_2_) and RNase E eluted by a gradient of elution buffer (20 mM Tris pH 7.9, 500 mM NaCl, 500 mM imidazole, 1 mM MgCl_2_). Protein quality was assessed by SDS–PAGE and fractions containing RNase E were pooled and loaded onto a butyl Sepharose column (GE Healthcare) equilibrated in high‐salt buffer (50 mM Tris pH 7.5, 50 mM NaCl, 25 mM KCl, 1 M (NH_4_)_2_SO_4_) to eliminate copurifying nucleic acids. A low‐salt buffer (50 mM Tris pH 7.5, 50 mM NaCl, 25 mM KCl, 5% v/v glycerol) was used for the elution. Based on purity as determined by SDS–PAGE, fractions were pooled, concentrated using a 50 kDa MWCO concentrator, and loaded onto a size‐exclusion column (Superdex 200 Increase 10/300, GE Healthcare) equilibrated in storage buffer (20 mM HEPES pH 7.0, 500 mM NaCl, 10 mM MgCl_2_, 0.5 mM TCEP, 0.5 mM ethylenediaminetetraacetic acid (EDTA), 5% v/v glycerol). The best fractions were flash‐frozen in liquid nitrogen and stored at −80°C until further use.

### RapZ expression and purification

Full‐length RapZ protein was expressed and purified by following a previously reported procedure (Gonzalez *et al*, 2017). Briefly, *Escherichia coli* Rosetta cells were transformed with plasmid pMCSG7 carrying the *gene* encoding full‐length RapZ with a Strep‐tag cleavable by TEV protease. Bacterial cultures were grown in LB media supplemented with 1% (w/v) glucose, 30 μg/ml chloramphenicol and 100 μg/ml carbenicillin at 37°C in dimpled flasks for optimal aeration and in an orbital shaker set at 220 rpm. The cultures were induced at OD_600_ ~ 0.8 by adding 1 mM IPTG, grown at 18°C for another 1 h and finally harvested by centrifugation at 5,020 *g* and 4°C for 30 min. Cell pellets were stored in lysis buffer (50 mM Tris pH 8.5, 500 mM KCl, 1 mM EDTA, 5 mM β‐mecaptoethanol) at −80°C until further use. Once thawed, the cell‐culture suspension was supplemented with DNase I and EDTA‐free protease inhibitor cocktail tablet (Roche) and cells were lysed by passing through an EmulsiFlex‐05 cell disruptor (Avestin) for 2–3 times at 10–15 kbar pressure. The lysate was clarified by centrifugation at 35,000 *g* for 30 min at 4°C and the supernatant was passed through a 0.45 μm membrane filter before loading onto a pre‐equilibrated Strep Trap HP column (GE Healthcare) equilibrated previously with Strep Trap buffer A (50 mM Tris pH 8.5, 500 mM KCl, 1 mM EDTA, 5 mM β‐mecaptoethanol). The column was washed extensively with buffer A before the protein was eluted with buffer B (50 mM Tris pH 8.5, 500 mM KCl, 1 mM EDTA, 5 mM β‐mercaptoethanol and 2.5 mM desthiobiotin). Protein quality was judged by SDS–PAGE and fractions containing RapZ were pooled. A final purification step was carried out by size‐exclusion chromatography using a Superdex 200 Increase 10/300 (GE Healthcare) column equilibrated with storage buffer (50 mM Tris pH 7.5, 100 mM NaCl, 50 mM KCl, 2 mM dithiothreitol). The fractions enriched in RapZ, as judged by SDS–PAGE, were flash‐frozen in liquid nitrogen and stored at −80°C until further use.

### RNA preparation by *in vitro* transcription

RNAs were produced by *in vitro* transcription. Templates were prepared by polymerase chain reaction (see Appendix Table S2 for oligonucleotides) and GlmZ and GlmZ‐Pro RNA were generated using T7 RNA polymerase at 37°C, followed by treating the reaction mixture with TURBO DNase for 15–20 min at 37°C to eliminate the DNA template. Synthesised GlmZ RNAs were purified on 6% 7.5 M urea polyacrylamide gel (National Diagnostics). The bands were visualised under a portable UV lamp at 254 nm wavelength and excised. Finally, the RNA was purified from the excised gel by overnight electroelution at 4°C and 100 V (EluTrap, Whatman). For all RNAs, purity was checked by 8% urea‐PAGE gel electrophoresis and SYBR gold RNA dye (Thermo Fisher) was used to visualise RNA (Appendix Fig S2d).

### CryoEM grid preparation

#### RNaseE‐NTD:RapZ:GlmZ complex

Purified RNase E‐NTD, full‐length RapZ and full‐length GlmZ RNA were incubated in a ratio of 5:25:10 μM in reaction buffer (25 mM HEPES pH 7.5, 300 KCl and 1 mM MgCl_2_) supplemented with 0.01% glutaraldehyde at RT for 20 min followed by at 30°C for 10 min. The cross‐linking reaction was quenched by adding 50 mM Tris pH 7.5. Samples were run on to a Superose 6 size‐exclusion column equilibrated with the reaction buffer. Fractions showing evidence for protein elution (judged by SDS–PAGE) with high 260/280 ratio were pooled, concentrated to 1 mg/ml and applied to Quantifoil Cu 300 1.2/1.3 grids coated with graphene oxide (GO) (Russo & Passmore, 2014; Palovcak *et al*, 2018). Briefly, grids were glow‐discharged on the darker carbon side in a PELCO easiGLOW glow discharge unit equipped with an oil pump (TED PELLA Inc., USA) using the following conditions: 15 mA, 0.28 mBar, 2 min. GO solution (2 mg/ml dispersion in water, Sigma‐Aldrich product code: 763705) was 10x diluted using ultrapure water (ddH_2_O) and centrifuged at 300 *g* for 30 s to remove insoluble GO flakes. The supernatant was further diluted another 10× to make a 0.02 mg/ml GO solution. On the glow‐discharged side of the Quantifoil grids, 1 μl of 0.02 mg/ml GO solution was applied and waited until the water evaporated. GO‐coated grids were left at room temperature for at least 12–16 h before use to prepare EM specimens.

### CryoEM data acquisition and processing

CryoEM data were collected on a Titan Krios G3 in the Department of Biochemistry, University of Cambridge, with parameters given in Table EV1. 2,445 movies were collected in accurate hole‐centring mode using EPU software (Thermo Fisher). CTF correction, motion correction and particle picking were performed using Warp (Tegunov & Cramer, 2019). 286,172 particles picked by boxnet2 masked neural network model in Warp were imported to CryoSPARC (Punjani *et al*, 2017) for all subsequent processing. These particles were initially subjected to two‐dimensional (2D) classification and 125,949 particles selected from these classes were used to generate initial *ab initio* 3D volumes representing RNaseE‐NTD:RapZ assemblies. The remaining 160,223 particles were also used to generate several ab initio 3D volumes to represent particles that do not contain RNaseE‐NTD:RapZ. Particles corresponding to different classes were selected and optimised through multiple iterative rounds of heterogeneous refinement as implemented in CryoSPARC. This process initially used the entire population of picked particles and all initial 3D volumes, and through the iterative process, particles not representing RapZ assemblies were discarded from the set used for reconstructions. Particles representing the ternary complex of RNaseE‐NTD:RapZ:GlmZ were subjected to particle subtraction to remove the variable part of the molecule (within a user‐defined masked area). The best models were then further refined using homogenous refinement and finally nonuniform refinement in CryoSPARC. The classification process is summarised schematically in the Fig EV1. The final reconstructions obtained had overall resolutions (Table EV1), which were calculated by Fourier shell correlation at 0.143 cut‐off (Figs EV3 and EV4).

### Structure refinement and model building

Crystal structures of full‐length RapZ (PDB:5O5O) (Gonzalez *et al*, 2017) and RNase E‐NTD (residues 1–510, PDBs: 2C0B, 6G63) (Callaghan *et al*, 2005; Bandyra *et al*, 2018) were initially placed into the cryoEM map. Further model building in COOT (Emsley & Cowtan, 2004) was followed by iterative cycles of refinement and density improvement in PHENIX (Afonine *et al*, 2018; Terwilliger *et al*, 2020). The RNA stem loop structures were predicted using the RNAfold web server (Lorenz *et al*, 2011) and subsequently subjected to the SimRNA server to generate 3D structures (Boniecki *et al*, 2016; Magnus *et al*, 2016). The structures were used as references to generate restraints for using PROSMART, and the models were docked into the cryoEM map and manually adjusted before local refinement.

## Author contributions

**Md. Saiful Islam:** Conceptualization; data curation; formal analysis; validation; investigation; visualization; methodology; writing—original draft; writing—review and editing. **Steven W Hardwick:** Data curation; formal analysis; visualization; methodology; writing—original draft; writing—review and editing. **Laura Quell:** Data curation; formal analysis; investigation; visualization; methodology; writing—original draft; writing—review and editing. **Svetlana Durica‐Mitic:** Data curation; formal analysis; methodology; writing—review and editing. **Dimitri Y Chirgadze:** Data curation; validation; methodology. **Boris Görke:** Conceptualization; formal analysis; supervision; funding acquisition; validation; investigation; methodology; writing—original draft; project administration; writing—review and editing. **Ben F Luisi:** Conceptualization; data curation; formal analysis; supervision; funding acquisition; validation; investigation; visualization; methodology; writing—original draft; project administration; writing—review and editing.

## Disclosure and competing interests statement

The authors declare that they have no conflict of interest.

## Supporting information



Appendix

Expanded View Figures PDF

Table EV1

Source Data for Appendix

PDF+

Source Data for Figure 3

## Data Availability

The models and maps have been deposited with the PDB and EMDB, with PDB entry 8B0I (https://www.rcsb.org/structure/8B0I) and corresponding EMDB entry ID EMD‐15784 (http://www.ebi.ac.uk/pdbe/entry/EMD‐15784); PDB entry ID 8B0J (https://www.rcsb.org/structure/8B0J) and corresponding EMDB entry ID EMD‐15785 (http://www.ebi.ac.uk/pdbe/entry/EMD‐15785).
