# Supplementary material for: Structure of a bacterial ribonucleoprotein complex central to the control of cell envelope biogenesis
Source: EMBO J. 2022 Dec 12;42(2):e112574. doi: 10.15252/embj.2022112574 (PMC9841335; doi:10.15252/embj.2022112574)
Supplement: Supplementary file 1 — Appendix [file EMBJ-42-e112574-s002.pdf]

Appendix

Structure of a bacterial ribonucleoprotein complex central to the control of cell envelope biogenesis

Md. Saiful Islam[1], Steven W. Hardwick[1], Laura Quell[2], Svetlana Durica-Mitic[2], Dimitri Y. Chirgadze[1], Boris Görke[2], Ben F. Luisi[1]

[1]Department of Biochemistry, University of Cambridge, Cambridge, CB2 1GA, UK

[2]Department of Microbiology, Immunobiology and Genetics, Max Perutz Labs, University of Vienna, Vienna Biocenter (VBC), Dr. Bohrgasse 9, 1030 Vienna, Austria

correspondence boris.goerke@univie.ac.at, bfl20@cam.ac.uk

TABLE OF CONTENTS

Table S1. Strains and plasmids used in this study	Page 2
Table S2. Oligonucleotides used in this study	Page 4
Figure S1. Metal-dependent nuclease activity of RNase E D346C	Page 5
Figure S2. Gel filtration and mass photometry profiles of the RNase E-NTD:RapZ:GlmZ complex	Page 6
Figure S3. Western blot analysis verifying synthesis of plasmid-encoded RapZ variants	Page 7
Figure S4. Analysis of protein-protein and protein-RNA interactions	Page 8
Figure S5. Heterogeneity of RNase E-NTD:RapZ:GlmZ ternary complexes	Page 9
Figure S6. Fundamental unit of RNase E-NTD:RapZ:GlmZ complex	Page 10
Figure S7. Conformational changes in the catalytic domain of RNase E	Page 12
Figure S8. Sequence alignment of RapZ homologues	Page 14

Appendix Table S1. Strains and plasmids used in this study.

Name	Relevant structure/genotype	Reference/construction[a]
E. coli K-12 strains:		
BTH101	*F⁻ cya-99 araD139 galE15 galK16 rpsL1 (Str^R) hsdR2 mcrA1 mcrB1*	(Karimova *et al,* 1998)
Z8	CSH50 *Δ*(*pho-bgl*)201 *Δ*(*lac-pro*) *ara thi ΔattB::*[*aadA, glmS'-lacZ*]	(Kalamorz *et al,* 2007)
Z28	as Z8, but *ΔrapZ*	(Kalamorz *et al,* 2007)
Z903	CSH50 *Δ*(*pho-bgl*)201 *Δ*(*lac-pro*) *ara thi, but ΔrapZ rne598-FLAG-cat*	(Gonzalez *et al,* 2017)
Plasmids:		
pBAD33	P_{Ara}, MCS 2, *cat*, ori p15A	(Guzman *et al,* 1995)
pBGG61	*rapZ* (-17 to +855) under P_{Ara}-control in pBAD33	(Göpel *et al*, 2013)
pBGG164	*strep-rapZ* under P_{tac} control, *lacI^q, bla*, ori ColEI	(Lüttmann *et al,* 2012)
pBGG348	encodes T25-RapZ in pKT25	(Göpel *et al,* 2013)
pBGG443	as pBGG61, but encodes RapZ with Lys170Ala substitution	this work, using [P]-oligo BG1235
pBGG449	encodes T25-RapZ-Ser239Ala in pKT25	this work, using [P]-oligo BG1350
pBGG453	as pBGG61, but encodes RapZ with Ser239Ala substitution	this work, using [P]-oligo BG1350
pBGG457	encodes T25-RapZ-Arg253Ala in pKT25	this work, using [P]-oligo BG1369
pBGG461	as pBGG61, but encodes RapZ with Arg253Ala substitution	this work, using [P]-oligo BG1369
pKT25	P_{lac}::*cyaA* [1-224] (T25), MCS, *neo*, *ori* p15A	(Karimova *et al*, 1998)
pLQ18	encodes T25-RapZ-His171Ala in pKT25	this work, using [P]-oligo BG2071
pLQ19	encodes T25-RapZ-Ile175Ala in pKT25	this work, using [P]-oligo BG2072
pLQ20	encodes T25-RapZ-Cys247Ser in pKT25	this work, using [P]-oligo BG1731
pLQ21	encodes T25-RapZ-Thr248Asp in pKT25	this work, using [P]-oligo BG2073
pLQ22	as pBGG61, but encodes RapZ with His171Ala substitution	this work, using [P]-oligo BG2071
pLQ23	as pBGG61, but encodes RapZ with Ile175Ala substitution	this work, using [P]-oligo BG2072
pLQ24	as pBGG61, but encodes RapZ with Cys247Ser substitution	this work, using [P]-oligo BG1731
pLQ25	as pBGG61, but encodes RapZ with Thr248Asp substitution	this work, using [P]-oligo BG2073
pLQ33	as pBGG61, but encodes RapZ with Asn236Ala substitution	this work, using [P]-oligo BG2115
pLQ34	encodes T25-RapZ-Asn236Ala in pKT25	this work, using [P]-oligo BG2115
pLQ44	as pBGG348, but encodes RapZ with Asn271Ala, Gln273Ala and Thr161Ala substitution	this work, using [P]-oligo BG2132 and BG2136
pLQ49	as pBGG348, but encodes RapZ with Asn271Ala, Gln273Ala, Tyr240Ala and Thr161Ala substitution	this work, using [P]-oligo BG2135, fragment template: pLQ44
pLQ51	as pBGG164, but encodes RapZ with Asn271Ala, Gln273Ala, Tyr240Ala and Thr161Ala substitution	this work, fragment template: pLQ49
pLQ53	as pYG97, but encodes T18-RNase E (aa 1-597) with	this work, using [P]-oligo

	ArgR141Ala, Arg142Ala and Arg169Ala substitution	BG2126 and BG2070
pLQ54	as pYG97, but encodes T18-RNase E (aa 1-597) with Arg357 Ala and Arg364Ala substitution	this work, using [P]-oligo BG2125
pLQ55	as pYG97, but encodes T18-RNase E (aa 1-597) with Lys106Ala and Arg109Ala substitution	this work, using [P]-oligo BG2116
pLQ56	as pLQ51, but encodes RapZ with additional His190Ala and Arg253Ala substitution	this work, using [P]-oligo BG1590 and BG1369
pLQ57	as pLQ51, but encodes RapZ with additional Arg253Ala substitution	this work, using [P]-oligo BG1369
pLQ58	as pBGG61, but encodes RapZ with Asn271Ala, Gln273Ala, Tyr240Ala, His190Ala and Thr161Ala substitution	this work, using [P]-oligo BGBG1590
pLQ59	as pBGG61, but encodes RapZ with Asn271Ala, Gln273Ala, Tyr240Ala and Thr161Ala substitution	this work, fragment template: pLQ49
pMCSG7	encodes RapZ with a Strep-tag cleavable by TEV protease	Gonzalez *et al*, 2017
pMN11	as pBGG164, but encodes RapZ with Lys170Ala substitution	this work, fragment template: pBGG443
pSD35	as pBGG164, but encodes RapZ with Arg253Ala substitution	this work, fragment template: pBGG457
pSD107	as pBGG61, but encodes RapZ with His190Ala substitution	this work, using [P]-oligo BG1590
pSD108	as pBGG61, but encodes RapZ with Thr248Ala substitution	this work, using [P]-oligo BG1591
pSD109	as pBGG61, but encodes RapZ with Gly249Trp substitution	this work, using [P]-oligo BG1592
pSD121	encodes T25-RapZ-His190Ala in pKT25	this work, using [P]-oligo BG1590
pSD122	encodes T25-RapZ-Thr248Ala in pKT25	this work, using [P]-oligo BG1591
pSD123	encodes T25-RapZ-Gly249Trp in pKT25	this work, using [P]-oligo BG1592
pSD125	as pBGG164, but encodes RapZ with Gly249Trp substitution	this work, fragment template: pSD109
pSD126	as pBGG164, but encodes RapZ with Thr248Ala substitution	this work, fragment template: pSD108
pSD130	as pBGG61, but encodes RapZ with Cys247Ala substitution	this work, using [P]-oligo BG1480
pSD134	as pBGG164, but encodes RapZ with His190Ala substitution	this work, fragment template: pSD121
pSD139	encodes T25-RapZ-Cys247Ala in pKT25	this work, using [P]-oligo BG1480
pSD169	as pBGG164, but encodes RapZ with Cys247Ala substitution	this work, fragment template: pSD130
pUT18C	P_{lac}::*cyaA* [*225-399*] (T18), MCS, *bla*, *ori* ColEI	(Karimova *et al*, 1998)
pYG97	encodes T18-RNase E (aa 1-597) fusion in pUT18C	(Göpel *et al*, 2013)

[a] [P]-oligo: 5' phosphorylated oligonucleotide for introduction of corresponding codon exchange into *rapZ.*

Appendix Table S2. Oligonucleotides used in this study.

Primer	Sequence	Res. Sites	Position
BG397	TGGCTGCAGTCTAGATTATCATGGTTTACGTTTTTCCAGCG	PstI, XbaI	*rapZ* +855 to +833
BG444	CTAATACGACTCACTATAGGGAGAGTAGATGCTCATTCCATCTCTTATG		*glmZ* +1 to +25
BG445	AAAAAAACGCCTGCTCTTATTACGGAGC		*glmZ* +207 to +180
BG637	GCGTCTAGAGATGGTACTGATGATCGTCAGCG	XbaI	*rapZ* +1 to +22
BG639	CGCGGTACCTCATGGTTTACGTTTTTCCAGCG	KpnI	*rapZ* +855 to +835
BG1049	GGCGAGCTCGTGAGGAGAAACAGTACATGGTACTGATGATCGTCAGCG	SacI	*rapZ* -17 to +22
BG1235	[P]-GAGTCTTTCGGCTTC**GC**ACACGGTATCCCTA		*rapZ* +493 to +523
BG1350	[P]-GAAACCAACAACCGT**GC**CTACTTGACGGTCG		*rapZ* +700 to +730
BG1369	[P]-GGCGGGAAGCAC**GC**TTCGGTGTATATTGCAG		*rapZ* +745 to +775
BG1480	[P]-CGGTCGCCATTGGT**GC**TACCGGCGGGAAG		*rapZ* +725 to +753
BG1590	[P]-CTTGCCGAACCCG**GC**CTGGGATCCGAAAC		*rapZ* +555 to +583
BG1591	[P]-GTCGCCATTGGTTGT**G**CCGGCGGGAAGC		*rapZ* +727 to +754
BG1592	[P]-GCCATTGGTTGTACC**T**G**G**GGGAAGCACCGTTC		*rapZ* +730 to +761
BG1731	[P]-GGTCGCCATTGGTT**C**TACCGGCGGGAAG		*rapZ* +726 to +753
BG2070	[P]-TGGGGCTTATCGTG**GCG**ACCGCTGGCGTCG		*rne* +491 to +520
BG2071	[P]-GTCTTTCGGCTTCAAA**GC**CGGTATCCCTATCG		*rapZ* +495 to +526
BG2072	[P]-CAAACACGGTATCCCT**GC**CGATGCAGATTACG		*rapZ* +507 to +538
BG2073	[P]-GTCGCCATTGGTTGT**GA**CGGCGGGAAGCAC		*rapZ* +727 to +756
BG2115	[P]-CTATGCTGGAAACCAAC**GC**CCGTAGCTACTTGACG		*rapZ* +692 to +726
BG2116	[P]-GTCATTGTTCAGATCGAT**GC**AGAAGAG**GC**CGGCAACAAAGGCGC		*rne* +298 to +341
BG2125	[P]-GTACGCCACCAG**GCT**GCGGTAGAAAACCGTCTG**GCT**GAAGCGGTGCGTC		*rne* +1057 to +1105
BG2126	[P]-GTGGCATTTCT**GCGGCT**ATCGAAGGCGAC		*Rne* +410 to +438
BG2132	[P]-CGCTCGCGCGGTAAA**GC**CGTC**GC**GTCACGCCATCGTAC		*rapZ* +796 to +833
BG2135	[P]-AACCAACAACCGTAGC**GC**CTTGACGGTCGCCATT		*rapZ* +702 to +735
BG2136	[P]-CGTGAACGCGAACTG**GC**CATGGTCTTTGAGTCTTT		*rapZ* +466 to +500
GlmZ forward	GAAATTAATACGACTCACTATAGTAGATGCTCATTCCATCTCTTATGTTC		
GlmZ rev	AAAAAAACGCCTGCTCTTATTACG		
GlmZ-Pro forward	GAAATTAATACGACTCACTATAGTAGATGCTCATTCCATCTCTTATGTTC		
GlmZ-Pro rev	CAACAAGTGGGTGCTTCACTC		

Restriction sites are underlined for the BG-primers; [P] denotes 5′-phosphorylation of oligonucleotide. Deviations from *wild-type* sequence are in bold. Position refers to the first nucleotide of the respective gene. The GlmZ primers were used to prepare PCR products for *in vitro* transcription.

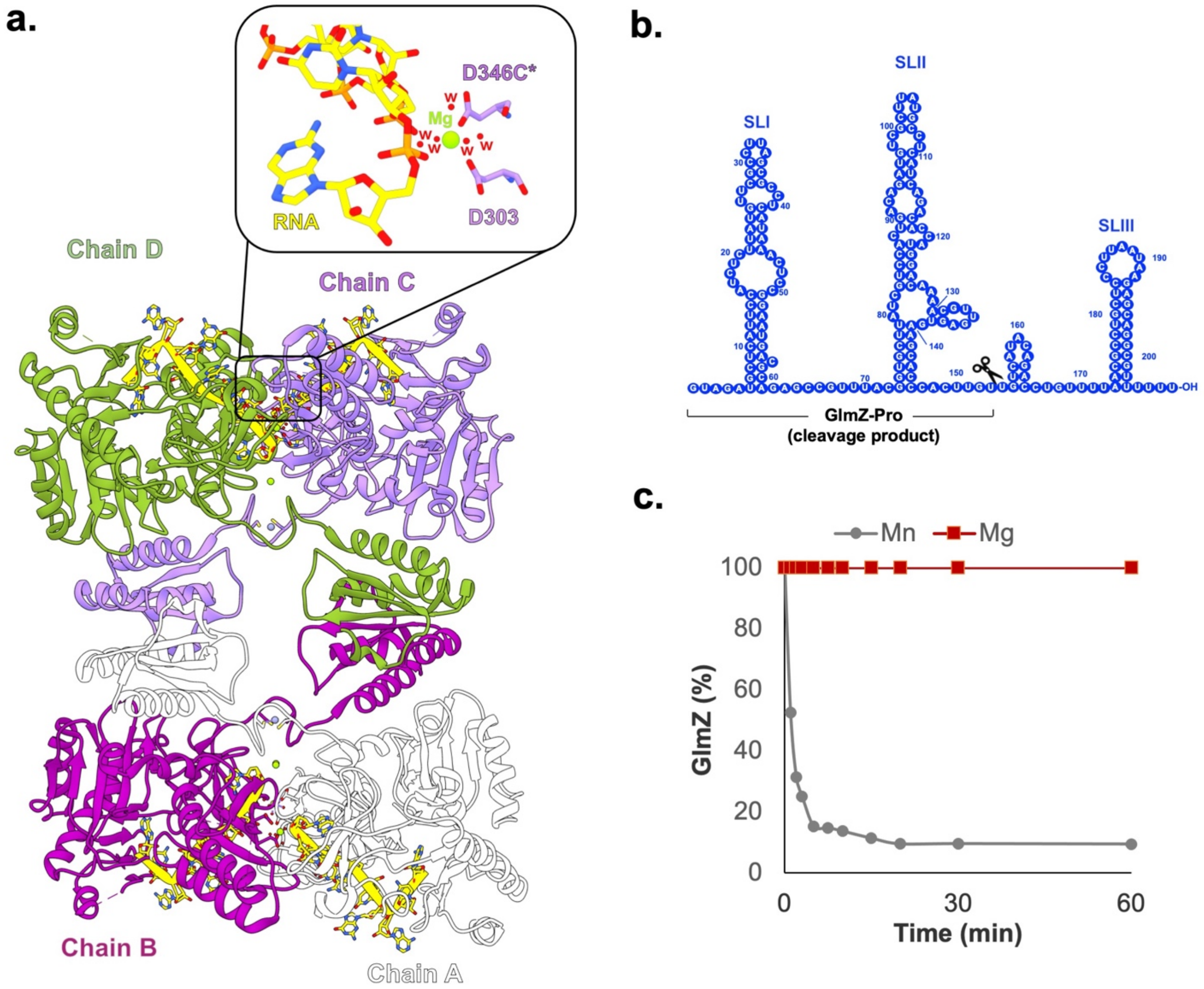

Appendix Figure S1. Metal-dependent nuclease activity of RNase E D346C. (a) Cartoon presentation of a crystal structure of RNase E-NTD in complex with RNA (PDB: 2C4R) (Callaghan *et al,* 2005). The RNase E variant used in our study carries a substitution of residue Asp346 with Cys (D346C*). The RNA is shown in yellow stick format, and the active site residues of the cleaving protomer are coloured light purple, magnesium ion as a green sphere and water molecules (w) as red spheres. (b) GlmZ RNA with a scissor showing the cleavage site (between SLII and SLIII) where RNase E cleaves, forming processed form of GlmZ (GlmZ-Pro). (c) Activity assays confirm that the D346C mutant is inactive with Mg^{2+} but highly active in the presence of Mn^{2+}. Ribonuclease cleavage of GlmZ (625 nM) by RNase E-NTD D346C (125 nM) in the presence of RapZ (250 nM) was carried out at 30º C in reaction buffer: 25 mM Tris-HCl pH 7.5, 50 mM NaCl, 50 mM KCl, 10 mM $MgCl_2$ or $MnCl_2$,1 mM DTT, 0.5 U/µL RNase OUT (Bandyra *et al,* 2018). Samples were quenched by adding proteinase K in 100 mM Tris-HCl pH 7.5, 150 mM NaCl, 12.5 mM EDTA, 1% SDS, followed by incubation at 50º C for 30 minutes. RNA samples were then mixed with loading dye (Thermo Fisher), heated at 95º C for 2 minutes and loaded onto 8% urea-PAGE gel. The gels were stained by SYBR® Gold (ThermoFisher) and reaction products were visualized under UV transilluminator (GeneSnap, Syngene). To quantify, intensity of the reaction products was calculated using GeneTools (Syngene) against known amounts of GlmZ (Bandyra *et al,* 2018).

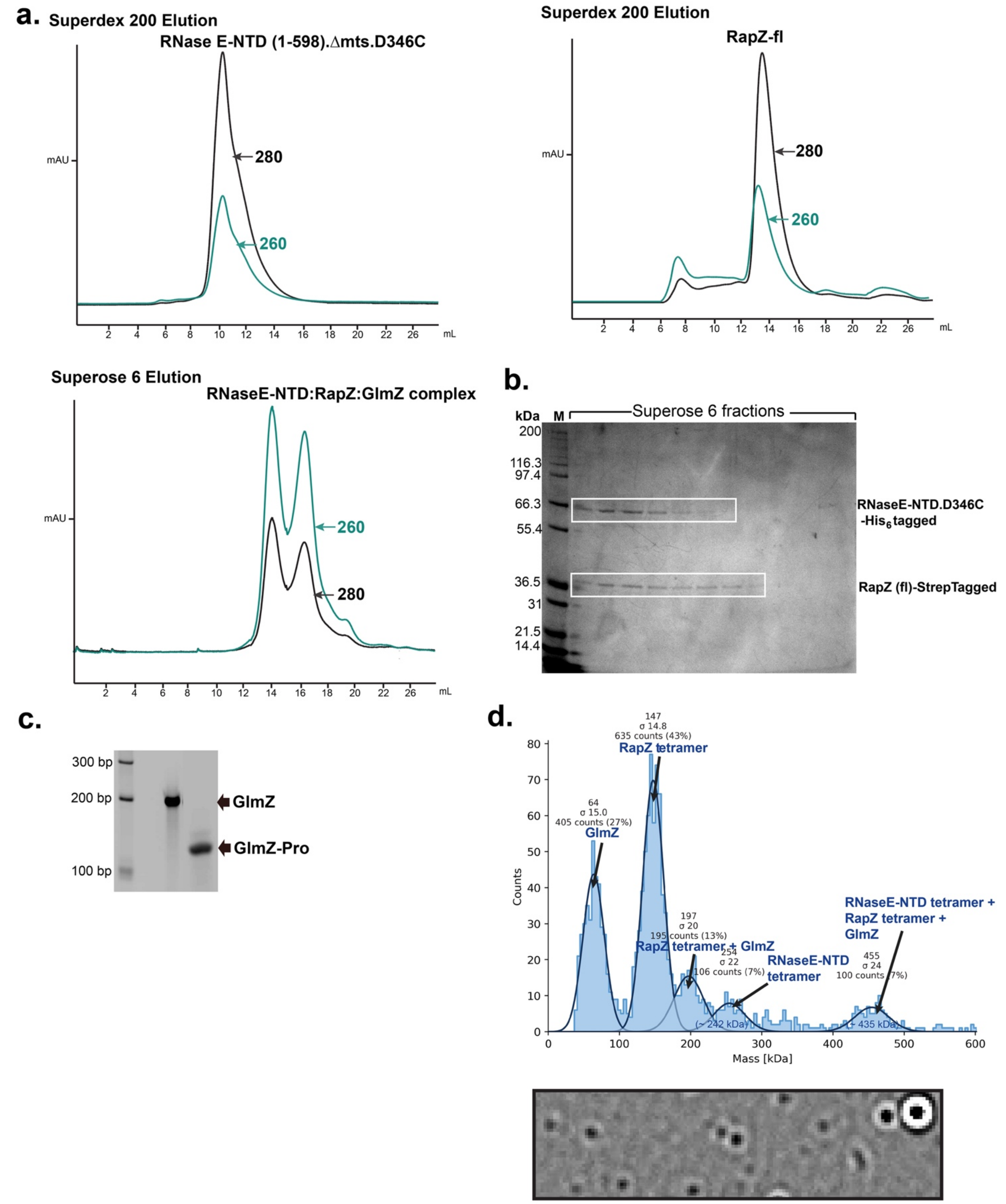

Appendix Figure S2. Gel filtration and mass photometry profiles of the RNase E-NTD:RapZ:GlmZ complex**.** (a) Size-exclusion chromatograms showing a high 260/280 nm absorption ratio for the RNase E-NTD:RapZ:GlmZ complex compared to RNase E-NTD or RapZ. (b) SDS-PAGE analysis confirming presence of RNase E NTD and RapZ in the Superose 6 elution fractions. (c) Denaturing RNA gel separating *in vitro* transcribed full-length GlmZ and processed GlmZ (GlmZ-pro) for size comparison. (d) Histogram fitted with gaussian functions for the RNaseE-NTD:RapZ:GlmZ complex showing a heterogenous mixture (Mass Photometer Refeyn One, Refeyn Ltd., UK) (Young *et al,* 2018); inset is a snapshot from the small movie of data collection showing protein scattering signals result in contrast (black spot surrounded by white ring). The buffer used was 25 mM HEPES pH 7.5, 300 KCl, 1 mM $MgCl_2$, 1mM dithiothreitol. RNase E NTD (1-

598).Δmts.D346C = RNase E residues 1 to 598 without the membrane targeting site (mts) and with active site mutation of Asp346 to Cys, RapZ-fl = full-length RapZ, GlmZ = full-length GlmZ (see Figure S1b), GlmZ-Pro = processed GlmZ (see Figure S1b), bp = base pairs.

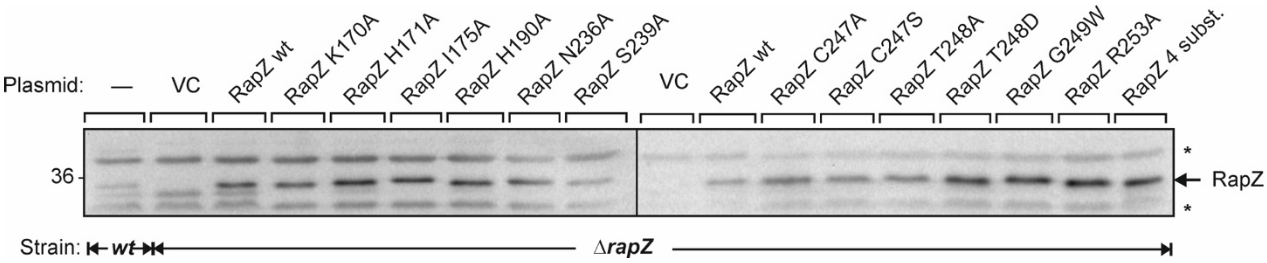

Appendix Figure S3. Western blot analysis verifying synthesis of plasmid-encoded RapZ variants. Total protein extracts of the arabinose-induced cultures tested in Figure 3b-c were separated by SDS-PAGE and gels were subsequently blotted. RapZ variants (indicated by arrow) were detected using a polyclonal antiserum against RapZ as described previously (Durica-Mitic *et al*, 2019). Non-specifically detected proteins are indicated with asterisks.

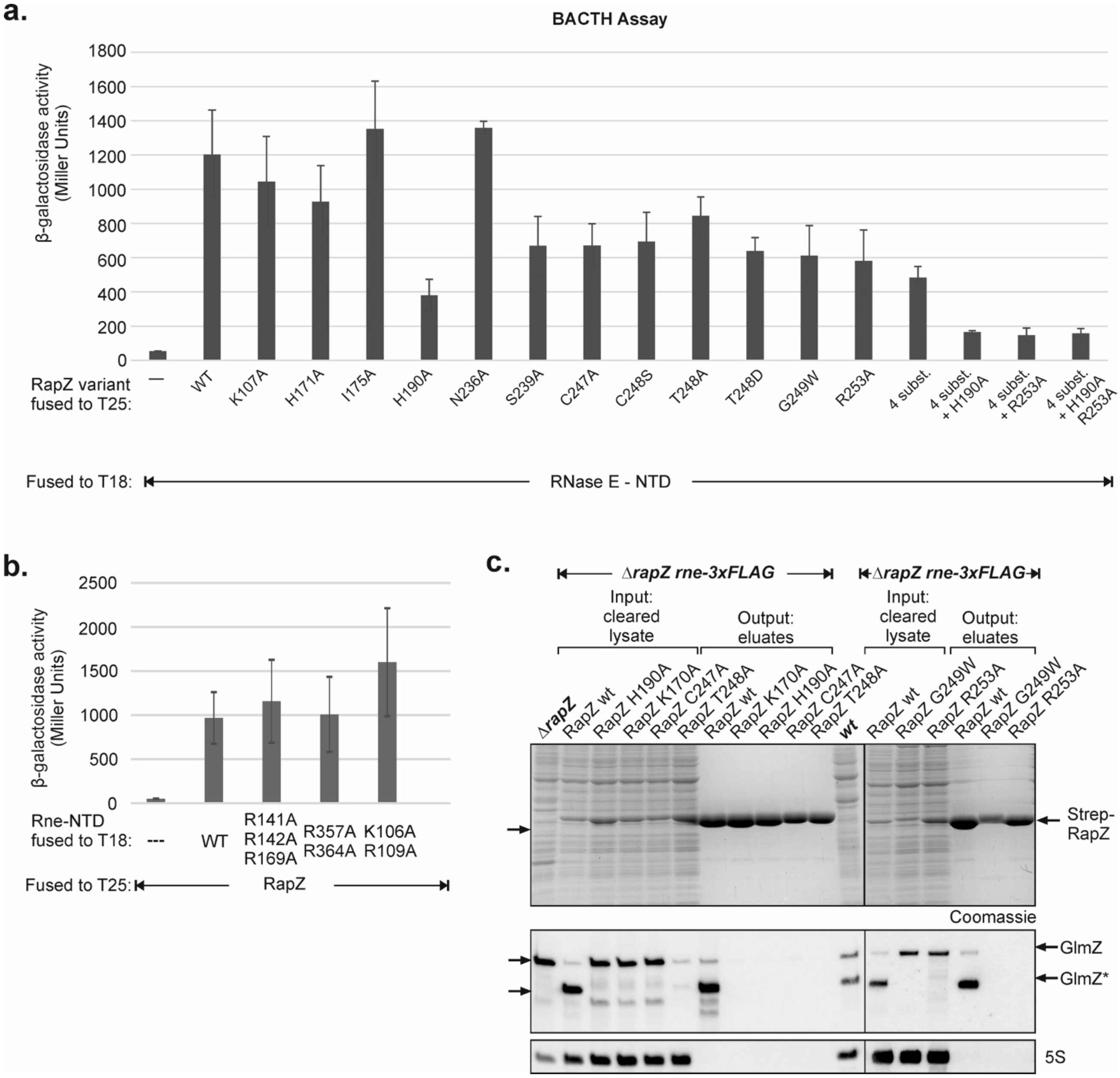

Appendix Figure S4. Analysis of protein-protein and protein-RNA interactions. (a,b) Bacterial adenylate cyclase-based two-hybrid (BACTH) assays to assess (a) interaction performance of RapZ variants with the RNase E-NTD and (b) RNase E-NTD variants with RapZ. BACTH measures efficiency of restoration of cAMP synthesis through interaction of proteins that are fused to split adenylate cyclase T18- and T25-fragments. Synthesis of endogenous β-galactosidase is measured as regulatory output. Reporter strain BTH101 lacks *cyaA* on the chromosome but carried two compatible plasmids encoding the T25- and T18-fragments either alone (pKT25/pUT18C vector control; column 1) or as fusion proteins with the indicated RapZ variant and the RNase E-NTD, respectively (cf. Table S2 for plasmids). Cells were grown to stationary phase in presence of 1 mM IPTG to allow for expression of the fusion genes from their plasmid-encoded *lac* promoters and for de-repression of the chromosomal *lac* operon. Average β-galactosidase activities including standard deviations are shown, which derive from at least three measurements and two independently transformed cell lines. (c) Pull-down using Strep-tagged RapZ wild-type and variants as bait-protein in a StrepTactin affinity chromatography. Cleared lysates (input) and eluates (output) derived from the affinity chromatography were separated by SDS-PAGE followed by Coomassie blue staining for analysis of protein content. Additional aliquots were used for RNA extraction to assess co-eluting GlmZ and GlmZ*. Loaded RNA was normalized to protein content in the elution fractions.

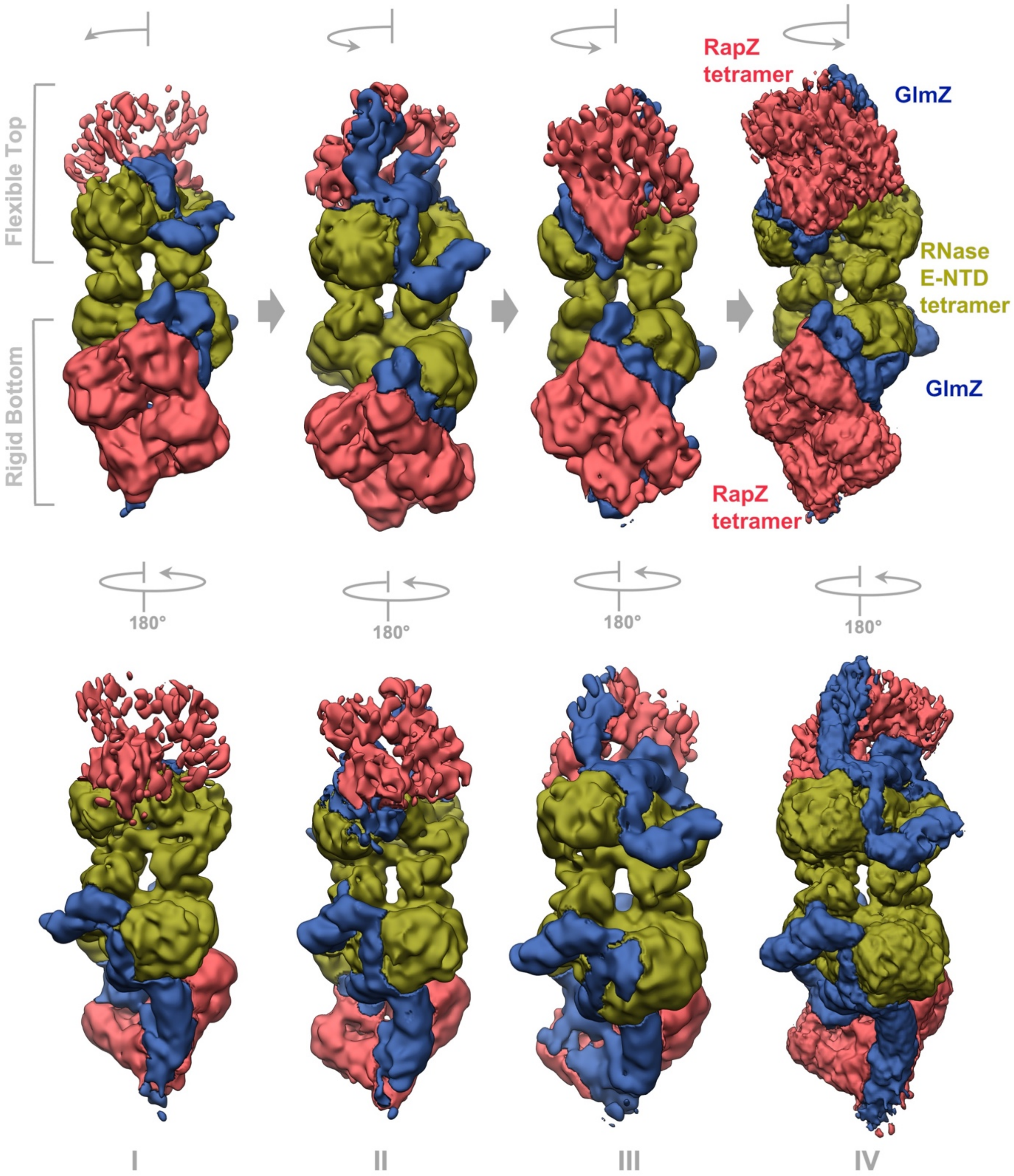

Appendix Figure S5. Heterogeneity of RNase E-NTD:RapZ:GlmZ ternary complex. Four assemblies (I-IV) of RNaseE-NTD:RapZ:GlmZ ternary complex revealing partial occupancy of RapZ:GlmZ binding to one of the RNase E-NTD dimers. Comparing classes II with III and IV, there is a *cis-/trans*-type binding of two RapZ tetramers with respect to each other on a RNaseE-NTD tetramer where one RapZ tetramer (bottom) is rigidly bound compared to the other which swivels around the apex of RNase E-NTD tetramer.

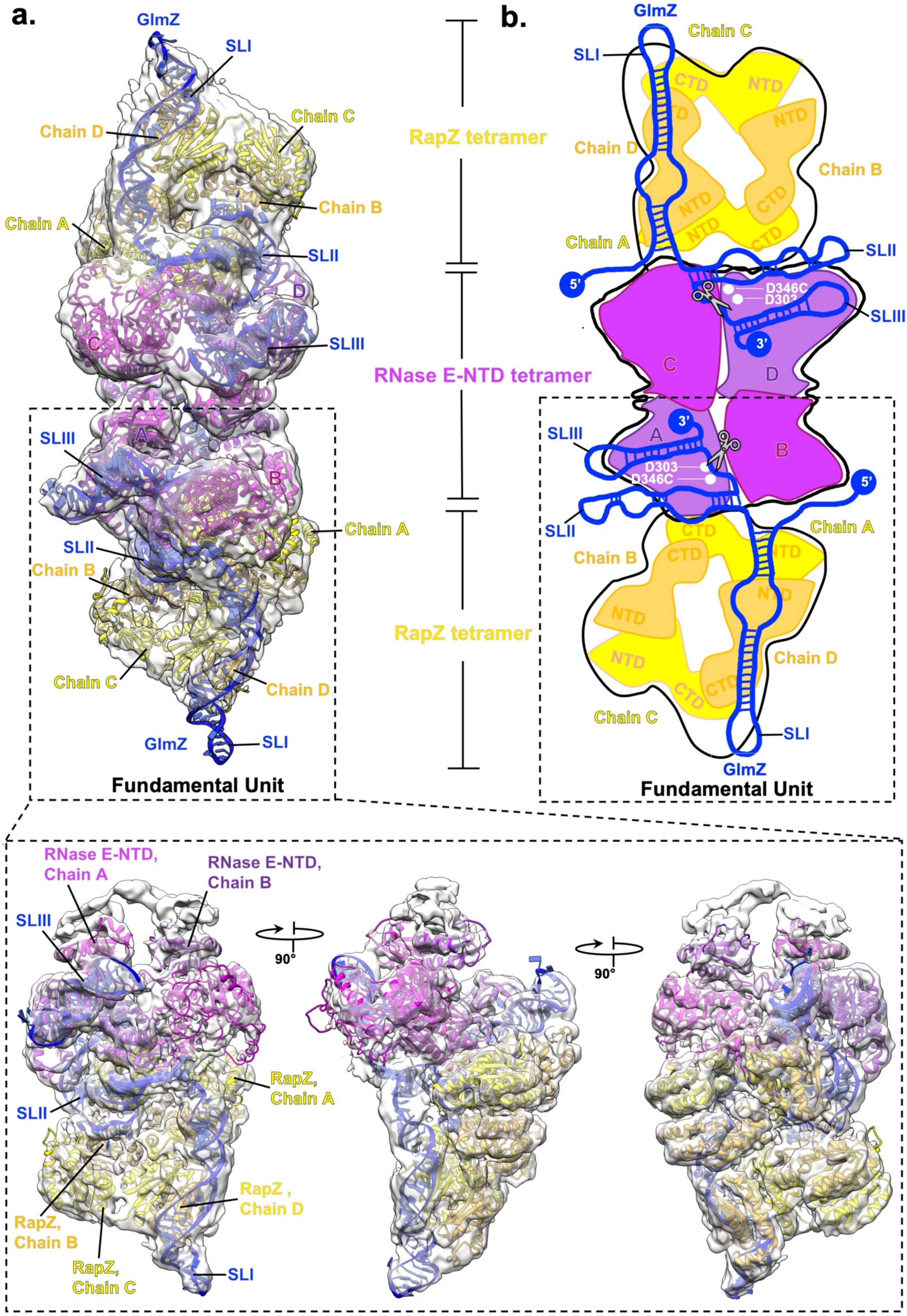
a.
GlmZ
SLI
Chain D
Chain C
Chain B
Chain A
SLII
D
C
SLIII
A
B
Chain A
SLII
Chain B
Chain C
Chain D
GlmZ
SLI
Fundamental Unit
b.
RapZ tetramer
RNase E-NTD tetramer
RapZ tetramer
GlmZ
Chain C
SLI
CTD
NTD
Chain D
Chain B
Chain A
SLII
5'
D346C
D303
SLIII
3'
C
D
3'
A
B
D303
D346C
5'
SLII
Chain A
Chain B
Chain D
Chain C
GlmZ
Fundamental Unit
RNase E-NTD, Chain A
RNase E-NTD, Chain B
SLIII
90°
90°
SLII
RapZ, Chain A
RapZ , Chain D
RapZ, Chain B
RapZ, Chain C
SLI

Appendix Figure S6. Fundamental unit of RNase E-NTD:RapZ:GlmZ complex. (a) A rigid body fit of the model of the RNase E-NTD:RapZ:GlmZ complex into the cryoEM map (white transparent) and (b) a cartoon representation of the model. A black box (dotted line) around the model and the cartoon indicates the fundamental unit of the complex; insets show orientation of the fundamental unit from three different angles. A scissor points the cleavage site of GlmZ RNA for the cleaving protomer of RNase E.

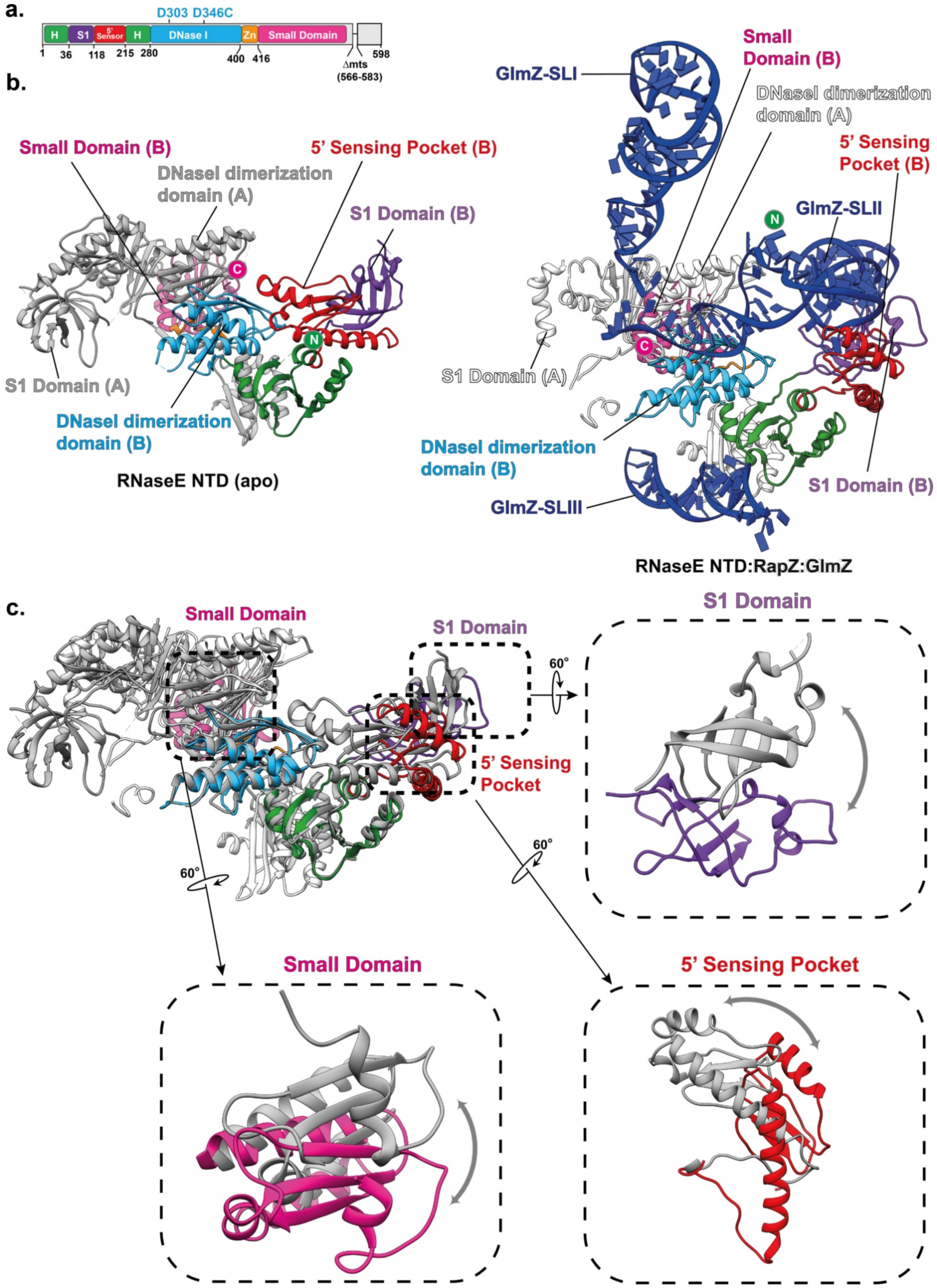

Appendix Figure S7. Conformational changes in the catalytic domain of RNase E-NTD. GlmZ-induced movement of S1, DNaseI-like, and 5'sensing domains of both cleaving and non-cleaving protomers of RNase E-NTD. (a) Domain topology of RNase E-NTD. (b) Left: apo-form of RNase E-NTD (one protomer shown as

grey and the other coloured as in scheme (a)) with 5'sensors/S1 domains in closed conformation (PDB: 2VMK) (Koslover *et al*, 2008); right: open conformation of 5'sensors/S1 domains of RNase E-NTD (one protomer shown as white and the other coloured as in scheme (a)) upon interaction with GlmZ. (c) Two structures presented in (b) were superimposed, but for clarity GlmZ was omitted from the RNaseE-NTD:RapZ:GlmZ complex structure. The dotted boxes highlight the movement of S1 domain (grey, apo-RNaseE-NTD; purple, RNaseE-NTD:RapZ:GlmZ complex), small domain (grey, apo-RNaseE-NTD; magenta, RNaseE-NTD:RapZ:GlmZ complex), and 5' sensing pocket (grey, apo-RNaseE-NTD; red, RNaseE-NTD:RapZ:GlmZ complex); arrows indicate the directionality of movements of individual domains as compared with RNase E-NTD apo-form (PDB: 2VMK).

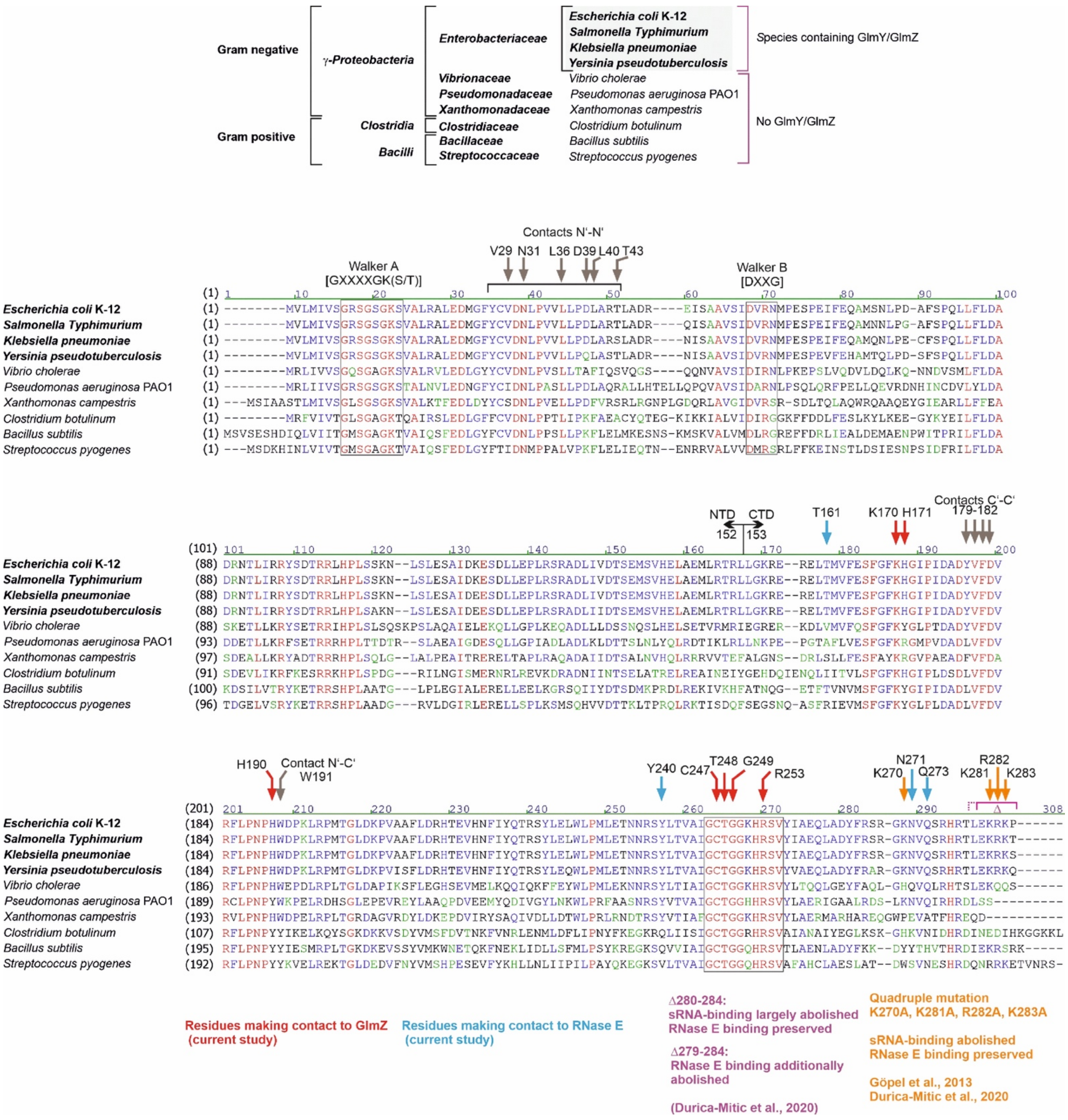

Appendix Figure S8. Sequence alignment of RapZ homologues. The sequences are highlighted for residues making contact to GlmZ (red arrows) and RNase E (blue arrows). Additional residues at the far C-terminus, which according to previous studies also contribute to binding of RNA or RNase E are indicated by orange arrows (Göpel *et al*, 2013) and purple brackets (Durica-Mitic *et al*, 2020), respectively. Occurrence of GlmY and GlmZ sRNAs in the various species is indicated at the top.

REFERENCES

Bandyra KJ, Wandzik JM, Luisi BF (2018) Substrate Recognition and Autoinhibition in the Central Ribonuclease RNase E. *Mol Cell* 72(2):275-285

Callaghan AJ, Marcaida MJ, Stead JA, McDowell KJ, Scott WG, Luisi BF (2005) Structure of Escherichia coli RNase E catalytic domain and implications for RNA turnover. *Nature* 437(7062):1187–1191

Durica-Mitic S, Göpel Y, Amman F, Görke B (2020) Adaptor protein RapZ activates endoribonuclease RNase E by protein-protein interaction to cleave a small regulatory RNA. *RNA* 26:1198-1215

Gonzalez GM, Durica-Mitic S, Hardwick SW, Moncrieffe MC, Marcus R, Neumann P, Ficner R, Görke B, Luisi BF (2017) Structural insights into RapZ-mediated regulation of bacterial amino-sugar metabolism. *Nucleic Acids Res* 45(18):10845–10860.

Göpel Y, Papenfort K, Reichenbach B, Vogel J, Görke B (2013) Targeted decay of a regulatory small RNA by an adaptor protein for RNase E and counteraction by an anti-adaptor RNA. *Genes Dev* 27:552–564.

Guzman LM, Belin D, Carson MJ, Beckwith J (1995) Tight regulation, modulation, and high-level expression by vectors containing the arabinose P_{BAD} promoter. *J Bacteriol* 177: 4121-4130

Kalamorz F, Reichenbach B, März W, Rak B, Görke B (2007) Feedback control of glucosamine-6-phosphate synthase GlmS expression depends on the small RNA GlmZ and involves the novel protein YhbJ in *Escherichia coli*. *Mol Microbiol* 65: 1518-1533

Karimova G, Pidoux J, Ullmann A, Ladant D (1998) A bacterial two-hybrid system based on a reconstituted signale transduction pathway. *Proc Natl Acad Sci USA* 95: 5752-5756.

Koslover DJ, Callaghan AJ, Marcaida MJ, Garman EF, Martick M, Scott WG, Luisi BF (2008) The Crystal Structure of the Escherichia coli RNase E Apoprotein and a Mechanism for RNA Degradation. *Structure* 16(8): 1238–1244

Lorenz R, Bernhart SH, Siederdissen CHZ, Tafer H, Flamm C, Stadler PF, Hofacker IL (2011) ViennaRNA Package 2.0. *Algorithms Mol Biol* 6(1): 26.

Lüttmann D, Göpel Y, Görke B (2012) The phosphotransferase protein EII^{Ntr} modulates the phosphate starvation response through interaction with histidine kinase PhoR in *Escherichia coli*. *Mol Microbiol* 86(1): 96-110

Young G, Hundt N, Cole D, Fineberg A, Andrecka J, Tyler A, Olerinyova A, Ansari A, Marklund E, Collier MP *et al.* (2018) Quantitative mass imaging of single biological macromolecules. *Science* 360(6387): 423-427.
