# Supplementary material for: Structure of a bacterial ribonucleoprotein complex central to the control of cell envelope biogenesis
Source: EMBO J. 2022 Dec 12;42(2):e112574. doi: 10.15252/embj.2022112574 (PMC9841335; doi:10.15252/embj.2022112574)
Supplement: Supplementary file 2 — Expanded View Figures PDF [file EMBJ-42-e112574-s007.pdf]

Expanded View Figures

Figure EV1. Workflow for cryoEM data collection and processing.

From the subset of particles giving good volumes, the heterogenous refinement separated the particles into two main groups, corresponding to binary and ternary complexes. Initial grid preparation with Quantifoil and Ultrafoil grids showed aggregation or visible particles (not shown). Better results were obtained with graphene oxide-covered grids.

© 2022 The Authors

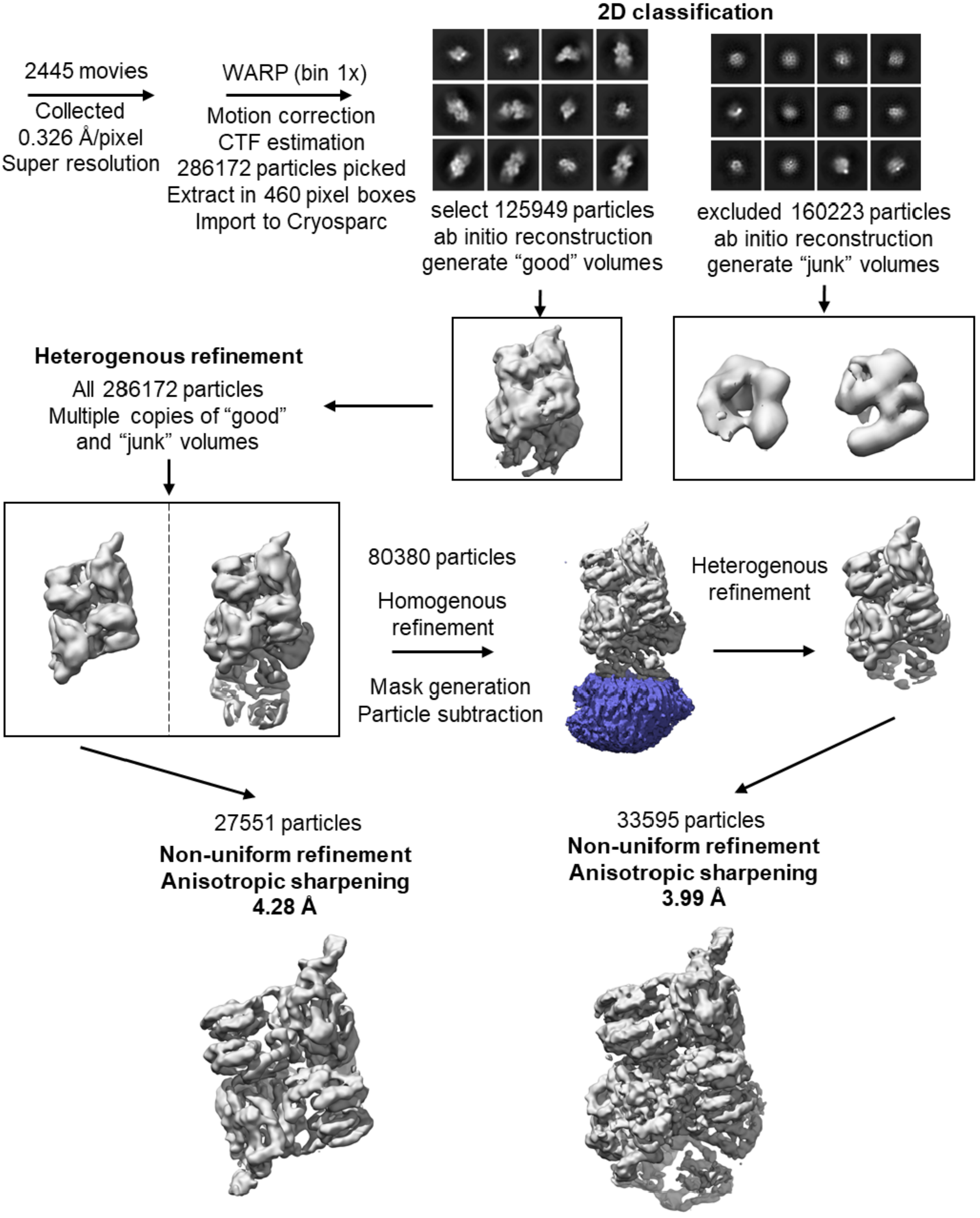

Figure EV1.

 © 2022 The Authors

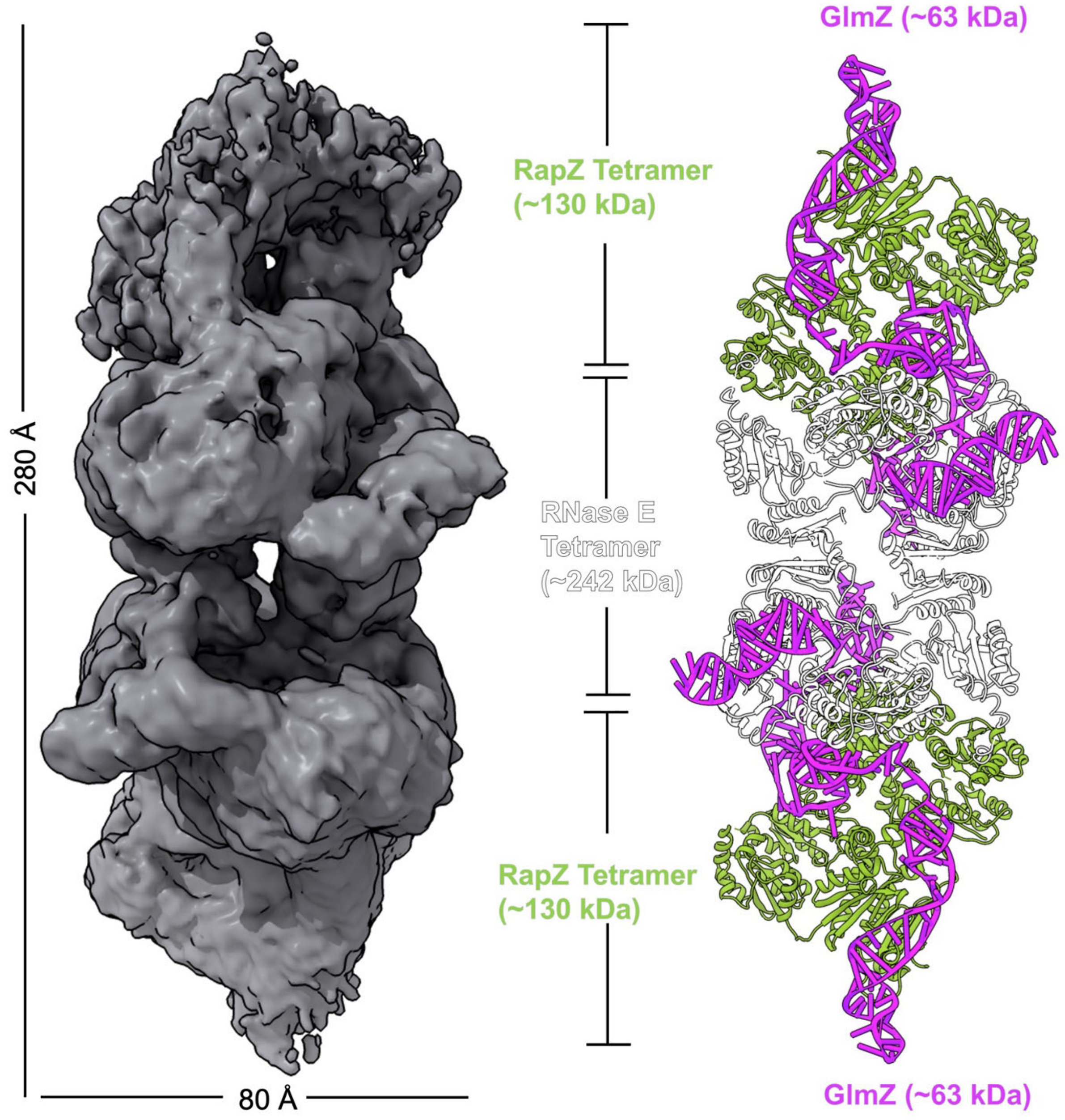

Figure EV2. CryoEM model of the ternary RNase E-NTD:RapZ:GlmZ complex.

The panel (left) shows the cryoEM map, which is in grey. The model (right) of RNase E-NTD:RapZ:GlmZ complex comprising one copy of RNase E-NTD tetramer (white), two copies of RapZ tetramer (olive) and two copies of GlmZ RNA (light purple). Previously reported crystal structures of RapZ (PDB: 5O5O) and RNase E-NTD (PDB:6G63) and a model of GlmZ RNA generated by ViennaRNA Package 2.0 (Lorenz *et al*, 2011) were used to build the model of the RNase E-NTD:RapZ:GlmZ complex.

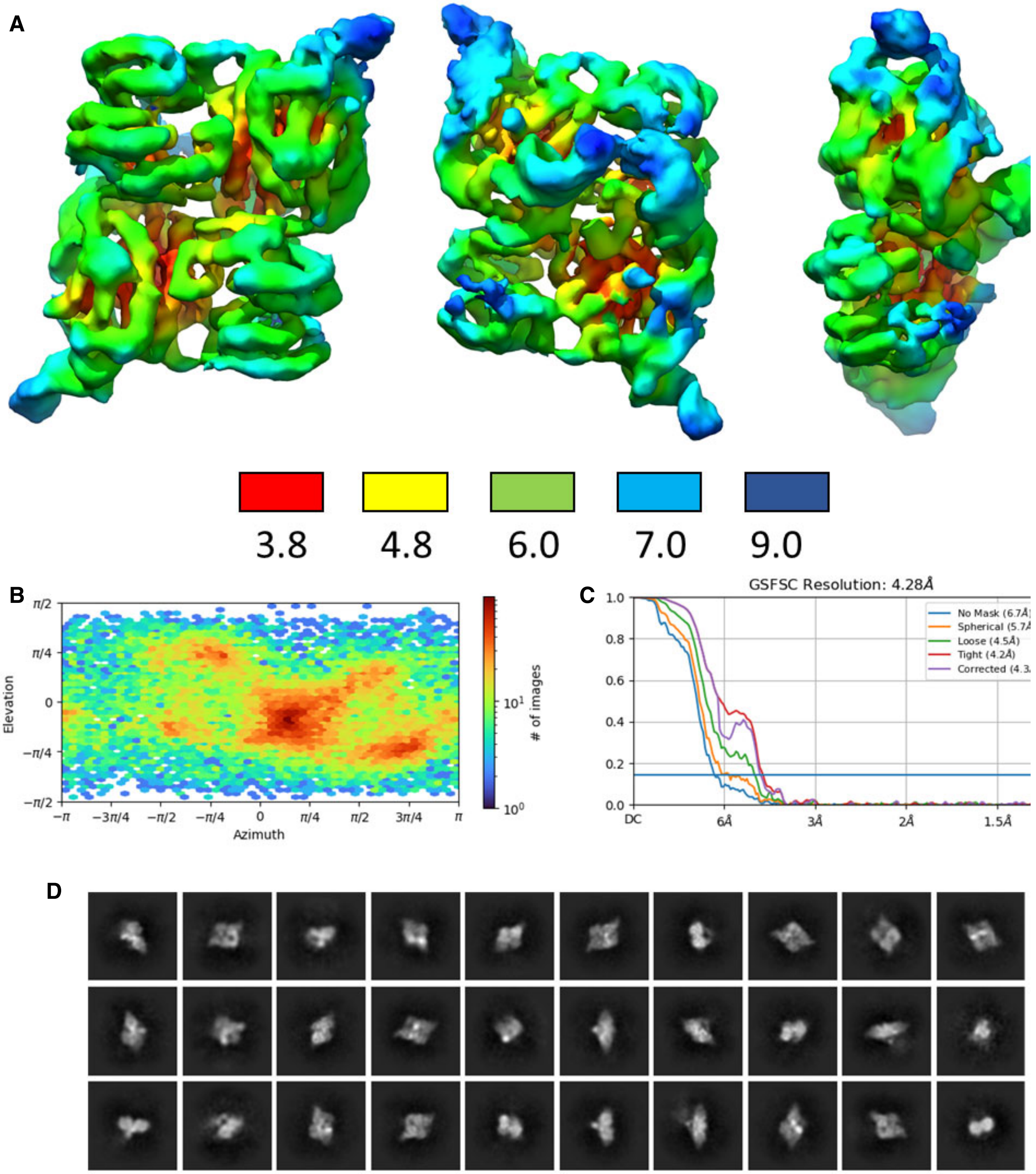

Figure EV3. Summary of cryoEM analysis of the RapZ:GlmZ binary complex.

A Three views of the local resolution map of the binary complex calculated in cryoSPARC and coloured by resolution in angstroms according to the key shown below the maps.

B Angular distribution calculated in cryoSPARC for particle projections contributing to the final map shown as a heat map. The colour coding for the heat map is shown in the bar to the right.

C Fourier shell correlation (FSC) resolution curves as calculated by cryoSPARC with different masks.

D 2D class images from particles contributing to the final binary RapZ:GlmZ complex cryoEM map.

© 2022 The Authors

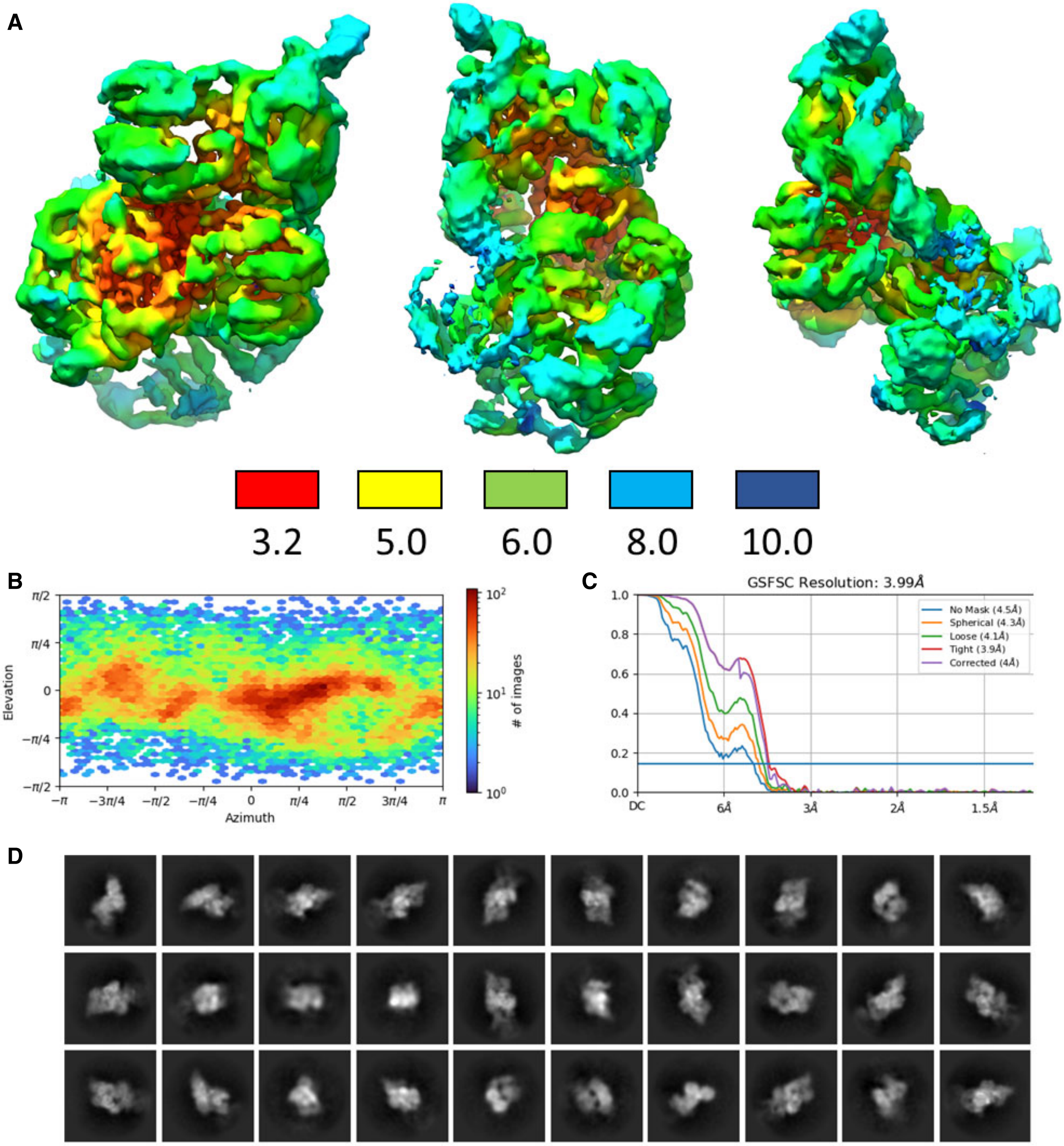

Figure EV4. Summary of cryoEM analysis of the RNase E-NTD:RapZ:GlmZ ternary complex.

A Local resolution map of the ternary complex calculated in cryoSPARC and coloured by resolution in angstroms according to the key below.
B Angular distribution calculated in cryoSPARC for particle projections contributing to the final map shown as a heat map.
C FSC resolution curves as calculated by cryoSPARC with different masks.
D 2D class images from particles contributing to the final cryoEM map of ternary complex RNaseE-NTD:RapZ:GlmZ.
