# Supplementary material for: Structure of a bacterial ribonucleoprotein complex central to the control of cell envelope biogenesis
Source: EMBO J. 2022 Dec 12;42(2):e112574. doi: 10.15252/embj.2022112574 (PMC9841335; doi:10.15252/embj.2022112574)
Supplement: Supplementary file 3 — Table EV1 [file EMBJ-42-e112574-s001.docx]

**Expanded view Table EV1. Cryo EM data collection and refinement statistics**

|  | Ternary | Binary |
| --- | --- | --- |
| **Data collection and processing** |  |  |
| Microscope  Detector | Titan Krios G3  Gatan K3 | Titan Krios G3  Gatan K3 |
| Magnification | 130,000 | 130,000 |
| Energy filter slit width (eV) | 20 | 20 |
| Voltage (kV) | 300 | 300 |
| Flux on detector (e/pix/sec) | 15.20 | 15.20 |
| Electron exposure on sample (e–/Å^2^) | 46.84 | 46.84 |
| Target defocus range (μm) | 1.2-2.6 | 1.2-2.6 |
| Calibrated pixel size (Å) | 0.652 | 0.652 |
| Symmetry imposed | C1 | C1 |
| Extraction box size (pixels) | 460 | 460 |
| Initial particle images (no.) | 286,172 | 286,172 |
| Final particle images (no.) | 33,595 | 27,551 |
| **Refinement** |  |  |
| Map resolution at FSC=0.143 (Å)* | 3.99 | 4.28 |
| Model composition |  |  |
| Non-hydrogen atoms | 19392 | 10848 |
| Protein residues | 2145 | 1126 |
| Nucleotides | 174 | 122 |
| B factor (Å^2^) |  |  |
| Protein | 239.82 | 250.07 |
| RNA | 281.69 | 256.37 |
| Bonds (RMSD) |  |  |
| Lengths (Å) | 0.013(33) | 0.014(17) |
| Angles (°) | 2.977(689) | 2.955(454) |
| Validation |  |  |
| Molprobity score | 2.45 | 2.57 |
| Clashscore | 19 | 20 |
| Poor rotamers (%) | 0.0 | 0.4 |
| Ramachandran plot |  |  |
| Favored (%) | 88.65 | 91.94 |
| Allowed (%) | 11.35 | 7.96 |
| Disallowed (%) | 0.0 | 0.1 |
