# Supplementary material for: Structure of a bacterial ribonucleoprotein complex central to the control of cell envelope biogenesis
Source: EMBO J. 2022 Dec 12;42(2):e112574. doi: 10.15252/embj.2022112574 (PMC9841335; doi:10.15252/embj.2022112574)
Supplement: Supplementary file 7 [file EMBJ-42-e112574-s005.pdf]

Structure of a bacterial ribonucleoprotein complex central to the control of cell envelope biogenesis

Md. Saiful Islam, Steven W. Hardwick, Laura Quell, Svetlana Durica-Mitic, Dimitri Y. Chirgadze, Boris Görke, Ben F. Luisi
DOI: 10.15252/embj.2022112574

Corresponding authors: Boris Görke (boris.goerke@univie.ac.at), Ben F. Luisi (bfl20@cam.ac.uk)

Review Timeline:

Transfer from Review Commons:	10th Sep 22
Editorial Decision:	3rd Oct 22
Revision Received:	16th Oct 22
Editorial Decision:	25th Oct 22
Revision Received:	26th Oct 22
Accepted:	28th Oct 22

Editor: Kelly Anderson

Transaction Report:

This manuscript was transferred to The EMBO Journal following peer review at Review Commons.

(Note: With the exception of the correction of typographical or spelling errors that could be a source of ambiguity, letters and reports are not edited. Depending on transfer agreements, referee reports obtained elsewhere may or may not be included in this compilation. Referee reports are anonymous unless the Referee chooses to sign their reports.)

Review #1

1. Evidence, reproducibility and clarity:

Evidence, reproducibility and clarity (Required)

Summary:

GlmS, the glucosamine-6-phosphate synthetase in E. coli and related bacteria, is essential, required for synthesis of both peptidoglycan and LPS. It is regulated at various levels, including positive regulation of GlmS translation by the Hfq-binding sRNA GlmZ. GlmZ activation of translation is regulated, indirectly, by the levels of GlcN6P, the product of GlmS. The components of the sensing and regulatory cascade have previously been defined, via genetics, biochemical and molecular biology studies. GlmZ is cleaved by Rnase E, becoming inactive, when GlcN6P levels are high, dependent upon the binding of GlmZ to RapZ. RapZ has been found to directly sense GlcN6P levels; another regulatory RNA, GlmY, also binds RapZ in the absence of GlcN6P, protecting GlmZ from RapZ-mediated processing.
The authors of this manuscript performed cryoEM to study the structure of two important complexes in this sensing cascade, RapZ/GlmZ and RapZ/GlmZ/RNase E-NTD, with the aim of clarifying how the RNA binding protein RapZ causes the cleavage of sRNA GlmZ by RNaseE. Some of the predictions for critical residues in the RapZ/GlmZ binary complex structure were investigated by mutagenesis RapZ to define essential resiudes for GlmZ cleavage; the results are consistent with the structure.

Major comments:

- Are the key conclusions convincing?
1. Given that this is basically a structural paper, the major questions would be whether the cryoEM reconstructions are accurate (appear to be consistent with general expectations) and whether there is clear evidence to support the physiological relevance of the structure. The tests of function are of two sorts:
- a) Effect of RapZ mutants in Fig. 3b-d. These tests show loss of RapZ function with various alleles, likely consistent with model (but as noted below, very difficult for the reader to identify on the structures in 3a). The implication is that these will interfere with GlmZ binding. Possibly direct tests of a couple of these mutants for GlmZ binding (or pull down of GlmZ from in vivo expressed protein) would further support the model. I note that the text says T248A was unaffected in cleavage, but seems to be much reduced in Fig. 3b, even if fusion activity is good.
- b) The ternary complex was tested primarily by the BACTH assay of some RapZ mutants (Fig. S11), that show a reduced interaction. This is not a particularly convincing assay for a number of reasons: 1) the effects are relatively modest (2x down, in an assay that is probably not very linear with interaction, 2) some with reduced interaction (S239A, T248A) had good activity (at least all those with full interaction seem to be functional); 3) Ternary complex suggests that RapZ mediates this interaction; this could be tested by deleting glmZ (and maybe glmY as well) from this BACTH strain. 4) the authors suggest that there are also important protein-protein interactions, based on some observed interactions, and support this with similarly difficult to interpret BACTH data from a previous paper for Rnase E-RapZ

interaction. Here, too, that is not the most compelling data (is this interaction RNA-independent?).
- Should the authors qualify some of their claims as preliminary or speculative, or remove them altogether?
Possibly the importance of RNAse E-RapZ direct interaction, without further proof that this actually is needed for function.
- Would additional experiments be essential to support the claims of the paper? Request additional experiments only where necessary for the paper as it is, and do not ask authors to open new lines of experimentation.
As noted above, further tests of RapZ mutants for RNA binding would be useful; if this has been done previously, needs to be presented.
Are there Rnase E residues that would be predicted by the model to be critical for the RapZ or GlmZ interaction but are not otherwise needed for activity? Would these disrupt either the BACTH results or activity in vivo?
- Are the suggested experiments realistic in terms of time and resources? It would help if you could add an estimated cost and time investment for substantial experiments.
Yes, they are. They are generally extrapolations from what is already in the paper or in previous studies by these groups.
- Are the data and the methods presented in such a way that they can be reproduced?
Yes.
- Are the experiments adequately replicated and statistical analysis adequate?
Yes.

Minor comments:

- Specific experimental issues that are easily addressable.
None noted.
- Are prior studies referenced appropriately?
Yes, they are. However, the paper could more clearly outline what is already known at the level of interactions of the molecules under study here.
- Are the text and figures clear and accurate?
1. In a number of places, the text and figure order/numbers are not correct. See Fig. S1 (p. 4), S2 (legends vs. figure panels).
2. Better labeling in many figures is needed. Clarify what is shown in Fig. S2d, and make the labels readable. Label the particle types in S3. Use schematics more (as in Fig. 4 and S8) to make it easier for reader to follow structure (for Fig. 2, for instance). It is very difficult to discern RapZ tetramer here. Fig. 3a, it is very difficult to see the residue numbers on the structures. Clearly identify the fructokinase-like domains. Label lanes in Fig. 3b, c, d. Indicate active site for RNase E. in Fig. 4, in schematic at least.
- Do you have suggestions that would help the authors improve the presentation of their data and conclusions?
Overall, clarify and highlight better how the structures here fit with what is already known about important sequences/regions of RapZ, GlmZ, and Rnase E, maybe color-coding parts of GlmZ shown to be important for RapZ recognition, etc.
Page 12, the second last row. Text after 'In this model...' can be simplified or removed because it is just a hypothesis.

2. Significance:

Significance (Required)

- Describe the nature and significance of the advance (e.g. conceptual, technical, clinical) for the field.

Rnase E is a major essential endonuclease in bacteria such as E. coli. How accessory proteins lead to its recognition and cleavage of regulatory RNAs such as GlmZ is not well understood at the structural level, and these structures provide important insight into that process. In addition, the GlmZ/RapZ regulatory circuit plays an important role in bacterial growth and pathogenesis, and understanding it at this level of detail will certainly open up possibilities for targeting this process in the future.
- Place the work in the context of the existing literature (provide references, where appropriate).

The components that go into the current structures have been studied previously, with publications on RapZ structure, analysis of critical regions within the GlmZ RNA, and demonstration of the domain of Rnase E involved in interactions with RapZ (Durica-Mitic et al, 2020; Khan et al, 2020, Gonzalez et al, 2017, among others), exactly how these fit together has not been known. Other RNA binding proteins that affect degradation have been reported, but are not fully understood, and ways in which the ribonuclease binds complex RNAs is not fully understood either.
- State what audience might be interested in and influenced by the reported findings.

This work should be of broad interested to the field of RNA-based regulation and RNA degradation, with particular interest for those working on these processes in bacteria.
- Define your field of expertise with a few keywords to help the authors contextualize your point of view. Indicate if there are any parts of the paper that you do not have sufficient expertise to evaluate.

Our expertise is in RNA-based regulation and microbial genetics; we are not able to critically evaluate the cryoEM analysis itself.

3. How much time do you estimate the authors will need to complete the suggested revisions:

Estimated time to Complete Revisions (Required)

(Decision Recommendation)

Between 1 and 3 months

4. Review Commons values the work of reviewers and encourages them to get credit for their work. Select 'Yes' below to register your reviewing activity at Publons; note that the content of your review will not be visible on Publons.

Reviewer Publons

Yes

Review #2

1. Evidence, reproducibility and clarity:

Evidence, reproducibility and clarity (Required)

Summary:

Islam et al present their characterization of the E. coli RapZ-GlmZ-RNase E ternary complex in this manuscript under review. In E. coli, the RNA binding protein RapZ facilitates cleavage of GlmZ sRNA by RNase E when intracellular concentrations of GlcN-6P are high; when GlcN-6P levels are low RapZ is titrated by GlmY sRNA and GlmZ sRNA promotes an increase in the translation and stability of the mRNA encoding GlcN-6P synthase, GlmS. Via Cryo-EM, the authors of this manuscript solve the structure of the binary RapZ:GlmZ (Fig. 2) and ternary RapZ:GlmZ:RNase Y (Fig. 4) complexes. Based on the apparent RapZ-sRNA binding sites in the solved structure of the binary complex, the authors make substitutions in residues suspected to be involved in RNA binding and measure the impact of these substitutions on cleavage of GlmZ and GlmZ-mediated activation of GlmS expression (Fig. 3). The authors find that some of the residues predicted to be involved in RNA binding based on their structural studies are also important for the cleavage of GlmZ, presumably by RNase E. Finally, the authors show via bacterial two-hybrid assays that some residues of RapZ necessary for GlmZ cleavage are also important for its interaction with RNase E (Fig. S11). I would suggest that the authors measure co-immunoprecipitation of GlmZ with tagged-RapZ with or without substitutions in the proposed RNA binding residues to resolve this issue. Alternatively, EMSAs could be performed.

Major comments:

Overall, the structural studies our impressive and provide considerable insight into the recognition of substrates by RapZ and RNase E. Given the dearth of solved structures of RNAs with their cognate RNA binding proteins, these results are very significant.

A limitation in this work is the lack of experiments directly testing whether or not the residues of RapZ that appear to be important for its interaction with the GlmZ sRNA in the authors' Cryo-EM structures actually have a significant role in RNA binding. In lieu of measuring GlmZ binding by RapZ, the authors measure GlmZ cleavage in strains expressing RapZ or particular variants harboring substitutions in residues that appear to play a role in sRNA binding (Fig. 3b); however, it is impossible for the authors to determine whether impairment of GlmZ cleavage by RNase E in their assays is due to lack of GlmZ binding to RapZ, extraordinarily tight binding of GlmZ to RapZ, changes in the orientation of GlmZ bound to RapZ, or conformational changes in RapZ that lead to disruption of direct RapZ-RNase E contacts. The lack of this empirical data supporting their structural studies becomes more salient as the authors attempt to test whether RapZ binding of GlmZ is important for its

interaction with RNase E via a bacterial two-hybrid assay. Since the authors have not directly examined the importance of particular RapZ residues on GlmZ binding, the authors' interpretation of their results from these assays is very speculative.

The authors state on page 7 that "the interaction of RapZ:GlmZ with RNase E does not involve conformational rearrangement of either RapZ or GlmZ". However, the arrangement of SLII relative to SLI appears different between the RapZ:GlmZ and RapZ:GlmZ:RNase E structures presented. Additionally, SLII appears entirely bound by RapZ in the binary complex (Fig. 2b), whereas in the structure of the ternary complex, SLII appears less associated with RapZ (Fig. S4b). A supplementary figure showing side-by-side the structure of GlmZ bound to RapZ solved in the presence or absence of RNase E may make clear whether any differences that exist in the conformation of RapZ and GlmZ between the binary and ternary complex structures.

Minor comments:

Figure S1 legend. Change "inactivate" to "inactive" or "inactivated"

Figure S2 legend. The description for "(d)" is for S2c and the text for "(c)" refers to the image in S2d.

Figure legend S5a and S9a. If resolution in the key is in angstroms, then it should be indicated.

Figure 1. The model appears to indicate that the apo-form of RapZ binds GlmZ and GlmY, whereas the GlcN-6P bound form does not. Moreover, in the discussion, the authors indicate that GlcN-6P interferes with GlmZ binding to RapZ. How does RapZ bind and cleave GlmZ when GlcN-6P levels are high, if GlcN-6P interferes with GlmZ binding? It would be useful for the authors to address this conundrum in their discussion.

Fig. S3B and C. While panels in Fig. S3B and C seemed well aligned, numbering of lanes would provide additional clarity.

Many bacterial species including Bacillus subtilis, Streptococcus pyogenes, and Clostridium botulinum have RapZ homologs that bear a tyrosine instead of a histidine residue at the position corresponding to H190 in E. coli RapZ. Would you expect this change to reduce GlmZ binding by RapZ or lead to change in RNA specificity based on your structural data? This may be useful to discuss in the manuscript.

Fig. S10. It is confusing to me that the yellow chain in the structure of RNase E is labeled as the DNase I-domain in the apo structure, whereas in the structure with RprA or GlmZ bound, this colored region is labeled as the 5' sensing domain.

On page 12, the authors appear to indicate that their structural studies of the RapZ-GlmZ-RNase E ternary complex could be informative with regards to how KH domain proteins in Gram-positive bacteria could present their substrates to RNase E. First of all, these bacteria lack RNase E and instead encode an evolutionarily distinct endoribonuclease (RNase Y). Secondly, I think that it is overreaching to state that these structural studies will inform us on how KH domain proteins such as KhpA/KhpB, which may or may not have a chaperoning function akin to Hfq in Gram-positive bacteria, present substrates to RNase Y. Regardless, if

this statement is to remain, the authors should make clear that is RNase Y and not RNase E that they are referring to.

2. Significance:

Significance (Required)

In my opinion, the significance of this work is in the achievement of high-resolution structures of the complexes of the RNA binding protein RapZ and the endoribonuclease RNase Y with RNA substrate bound. There are very few structures solved of RNA binding proteins or RNases with their cognate substrates. This is likely due to the difficult in obtaining high resolution data for the bound RNA that may have a large degree of flexibility or many alternative conformations. More structures like this are needed to advance our understanding of RNA-protein interactions.

I believe that these findings would not only be of great interest to those that study small regulatory RNAs, such as myself, but also others more generally interested in RNA binding proteins, RNases, or protein-RNA interactions.

Field of expertise: small regulatory RNAs, RNA chaperones, RNases

Referees cross-commenting

1. I agree with Reviewer #1 that the results of the bacterial two-hybrid assay would be more informative, if the authors tested the impact of deletion of glmZ on the ability of the wild type and mutant RapZ proteins to interact with RNase Y by this assay.
2. As both reviewer #1 and I indicated, I think that it would be useful for the authors to directly assess the effect of key substitutions in RapZ on GlmY binding by a more direct measure of interaction, e.g., CoIP or EMSA.
3. I do think that it would be nice at some point for the authors to actually provide evidence that GlcN6P binds to the site that they predict as reviewer 3 suggested but this may be be beyond the scope of this manuscript and may be better addressed in another manuscript in which the authors solve the structure of RapZ with GlcN6P bound. In the meantime, the authors could limit their speculation.

3. How much time do you estimate the authors will need to complete the suggested revisions:

Estimated time to Complete Revisions (Required)

(Decision Recommendation)

Between 1 and 3 months

4. Review Commons values the work of reviewers and encourages them to get credit for their work. Select 'Yes' below to register your reviewing activity at Publons; note

that the content of your review will not be visible on Publons.

Reviewer Publons

Yes

Review #3

1. Evidence, reproducibility and clarity:

Evidence, reproducibility and clarity (Required)

Summary:

The biogenesis of the bacterial cell envelope relies on glucosamine-6-phosphate (GlcN6P), which is mediated by GlmZ and the sRNA-binding protein RapZ. GlmZ stimulates translation of the GlcN6P synthetase. When the levels of the GlcN6P are sufficiently high, RapZ will presents GlmZ to the endoribonuclease RNase E for cleavage and thereby silencing synthesis of the GlcN6P synthetase. However, how RapZ recruit RNase E to GlmZ for degradation is still unsolved. This paper reports the cryoEM structure of the binary complex of RapZ: GlmZ and the ternary complex of the RNase E catalytic domain (RNase E-NTD), RapZ and GlmZ. RapZ interacts with SLI and SLII of GlmZ through complementarity in shape and electrostatic charge to the phosphodiester backbone of the sRNA and presents the sRNA by alignning its SSR comprising the cleavage site into the RNase E active center. This paper suggests a general RNase E recognition pathway for complex substrates, which will help to understand the mechanisms that other RNA chaperones such as Hfq might work in an analogous assembly to present base-paired sRNA/mRNA pairs for cleavage. In total, this is an excellent work. I will support the publication of it until these following points are presented.

Major comments:

1. It was mentioned on Page 5 that "Sulphate and malonate ions were previously seen at these positions in crystal structures of apo RapZ" and pn Page 11 that " Interestingly, the phosphate groups of the RNA backbone occupy positions in RapZ that were previously observed to bind sulphate or malonate ions in the crystal structure of apo-RapZ, suggesting that this pocket could be the binding site for a charged metabolite such as GlcN6P". Is there any following experiments to investigate it further? If possible, I suggest the author to confirm that weather RapZ has the binding activity with GlcN6P or not.
2. "The kinase-like N-terminal domain of RapZ (NTD) makes only a few interactions with the RNA, and the path of the RNA does not encounter the Walker A or B motifs (Figure 2b). It is possible that this domain could act as an allosteric switch, whereby the binding of an as yet unknown ligand triggers quaternary structural changes that affect RapZ functions." Is there any more structural information supporting it? If the domain act as an allosteric switch, is it possible to make some deletion or substitution to test it?

3. Is there any results to compare the binding affinity of GlmY and GlmZ with RapZ?

Minor comments:

1. Page 8, is it "stabilised" or "stabilized", please check it.
2. The legends for Figure S2 c and d are reversed.
3. It was suggested to show the RNA molecules in Figure S1a.

2. Significance:

Significance (Required)

This paper suggests a general RNase E recognition pathway for complex substrates, which will help to understand the mechanisms that other RNA chaperones such as Hfq might work in an analogous assembly to present base-paired sRNA/mRNA pairs for cleavage.
In total, this is an excellent work.

3. How much time do you estimate the authors will need to complete the suggested revisions:

Estimated time to Complete Revisions (Required)

(Decision Recommendation)

Less than 1 month

4. Review Commons values the work of reviewers and encourages them to get credit for their work. Select 'Yes' below to register your reviewing activity at Publons; note that the content of your review will not be visible on Publons.

Reviewer Publons

Yes

July 30, 2022

RE: Review Commons Refereed Preprint #RC-2022-01224

Reviewer #1 (Evidence, reproducibility and clarity (Required)):

Summary:
GlmS, the glucosamine-6-phosphate synthetase in E. coli and related bacteria, is essential, required for synthesis of both peptidoglycan and LPS. It is regulated at various levels, including positive regulation of GlmS translation by the Hfq-binding sRNA GlmZ. GlmZ activation of translation is regulated, indirectly, by the levels of GlcN6P, the product of GlmS. The components of the sensing and regulatory cascade have previously been defined, via genetics, biochemical and molecular biology studies. GlmZ is cleaved by Rnase E, becoming inactive, when GlcN6P levels are high, dependent upon the binding of GlmZ to RapZ. RapZ has been found to directly sense GlcN6P levels; another regulatory RNA, GlmY, also binds RapZ in the absence of GlcN6P, protecting GlmZ from RapZ-mediated processing.
The authors of this manuscript performed cryoEM to study the structure of two important complexes in this sensing cascade, RapZ/GlmZ and RapZ/GlmZ/RNase E-NTD, with the aim of clarifying how the RNA binding protein RapZ causes the cleavage of sRNA GlmZ by RNaseE. Some of the predictions for critical residues in the RapZ/GlmZ binary complex structure were investigated by mutagenesis RapZ to define essential resiudes for GlmZ cleavage; the results are consistent with the structure.

Major comments:

- Are the key conclusions convincing?
1) Given that this is basically a structural paper, the major questions would be whether the cryoEM reconstructions are accurate (appear to be consistent with general expectations) and whether there is clear evidence to support the physiological relevance of the structure. The tests of function are of two sorts:
a) Effect of RapZ mutants in Fig. 3b-d. These tests show loss of RapZ function with various alleles, likely consistent with model (but as noted below, very difficult for the reader to identify on the structures in 3a). The implication is that these will interfere with GlmZ binding. Possibly direct tests of a couple of these mutants for GlmZ binding (or pull down of GlmZ from in vivo expressed protein) would further support the model. I note that the text says T248A was unaffected in cleavage, but seems to be much reduced in Fig. 3b, even if fusion activity is good.

Our reply. We have made further tests of the mutations for GlmZ binding. Using electrophoretic mobility shift assays, we observe reduced GlmZ binding affinities for RapZ mutants K170A, H190A, C247A, T248A (figure below). We also tested the activity of RapZ variant with 4 substitutions at the proposed RapZ/NTD interface (right lanes in figure below).

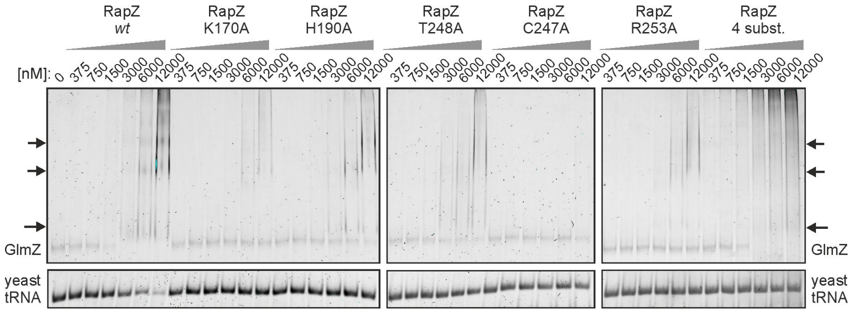

We followed the recommendation of the reviewer and performed co-purification experiments ("pull-down") using StrepTactin affinity chromatography and Strep-tagged RapZ variants as baits. Eluates were assessed for RapZ protein content and co-eluting GlmZ and processed GlmZ* sRNAs using Northern blotting. These new results, which have been incorporated in Fig. S7c, show that all tested RapZ variants except for the wild-type protein are not capable to pull-down GlmZ or GlmZ* in cell extracts. This includes the RapZ-T248A variant, which as noted by the referee is nonetheless still capable to decrease full-length GlmZ to some extent, albeit processed GlmZ* is hardly detectable (Fig. 3b, lanes 23, 24). To address this issue further, we purified the RapZ-T248A variant and some additional variants for comparison and performed EMSA. Globally, the EMSAs confirm the co-purification experiments, i.e. they demonstrate strongly reduced GlmZ binding activity for most tested RapZ variants, but also show that the RapZ-T248 variant kept some residual binding activity. This may explain the weak signal for processed GlmZ in the Northern blot (Fig. 3b) as processed GlmZ* likely binds to RapZ for stabilization. Similar effects were previously seen for the $\text{RapZ}_{\text{quad}}$ and the $\text{RapZ}_{\text{1-279}}$ variants in Durica-Mitic et al. 2020 (Fig. 5). Accordingly, we also changed our wording concerning the RapZ-T248A variant in the text. We have not incorporated the EMSA figure into the updated manuscript.

b) The ternary complex was tested primarily by the BACTH assay of some RapZ mutants (Fig. S11), that show a reduced interaction. This is not a particularly convincing assay for a number of reasons: 1) the effects are relatively modest (2x down, in an assay that is probably not very linear with interaction, 2) some with reduced interaction (S239A, T248A) had good activity (at least all those with full interaction seem to be functional); 3) Ternary complex suggests that RapZ mediates this interaction; this could be tested by deleting glmZ (and maybe glmY as well) from this BACTH strain. 4) the authors suggest that there are also important protein-protein interactions, based on some observed interactions, and support this with similarly difficult to interpret BACTH data from a previous paper for Rnase E-RapZ interaction. Here, too, that is not the most compelling data (is this interaction RNA-independent?).

Our reply: Previous work already indicated that formation of the ternary complex involves multiple interactions – direct protein-protein contacts but also indirect interactions mediated by sRNA GlmZ. For instance, *in vitro* pull-down signals (RapZ = prey; RNase E = bait) become reduced but not abolished when RNA-free protein preparations are used (Durica-Mitic et al., 2020; Fig. 2E). BACTH signals are reduced 2-fold when using RNase E and RapZ variants that are strongly impaired in their

RNA-binding capabilities, respectively (Durica-Mitic et al., 2020; Fig. 2C). As the BACTH assays and *in vitro* pull-down approaches yield similar trends, we suggest that BACTH experiments represent a useful approach to clarify the questions under study.

Point b1: To demonstrate that removal of multiple interactions is required to disrupt the ternary complex, we combined substitutions of residues making contact to the sRNA as well as residues directly contacting RNase E. According to the structure of the ternary complex presented here, residues T161, Y240, N271 and Q273 in RapZ are proposed to contact RNase E directly. Upon substitution of these four latter residues, resulting in the RapZ variant named RapZ-4 subst., the BACTH signal decreases two-fold – similar to what is observed for the RapZ variants that carry Ala substitutions of residues involved in sRNA-binding, such as H190 or R253. Importantly, when the latter two substitutions are introduced into the RapZ-4 subst. variant – either alone or in combination, the BACTH signal is reduced to almost back-ground levels. These results are in agreement with the features of the ternary complex proposed here and also with data obtained previously: They show that protein-protein and protein-RNA contacts must be concomitantly removed to disrupt the complex completely. We integrated the latter data as Fig. S7a in the revised manuscript and discuss the data at the appropriate positions in the text.

Point b2: In our opinion, the data reporting regulatory activity of the individual RapZ variants (Fig. 3 b-d) correlate well with the BACTH data (Fig. S7a): RapZ variants carrying substitutions of residues I175 and N236 retain regulatory activity and concomitantly a high RNase E interaction potential indistinguishable from the wild-type is observed. In contrast, RapZ variants carrying substitutions affecting sRNA-binding, i.e. H190A, C247A, C247S, T248D, G249W, R253A loose activity completely and concomitantly show a 2-fold decrease in the BACTH signal. The remaining BACTH signal is explained by the remaining (protein-protein) contacts as discussed above (point b1). Therefore, these variants are likely uncapable to present GlmZ in a correct manner to RNase E even though interaction is retained to some degree.

Only the RapZ mutants with exchanges H171A, S239A and T248A do not follow either of these two scenarios: albeit they exhibit reduced interaction with RNase E according to BACTH, they retain the ability to regulate the chromosomal *glmS'-lacZ* fusion, at least when produced from a plasmid (Fig. 3d). However, inspection of the GlmZ Northern blot signals (Fig. 3b) reveals that full-length GlmZ is decreased as expected, but that processed GlmZ* becomes either not visible or is much reduced when compared to wild-type RapZ. This explains by a reduced sRNA binding affinity, as pointed out above (point 1a), which also provides a rationale for the decreased BACTH signal.

Point b3: We agree that deletion of *glmZ* in the BACTH strain would be an ideal approach to dissect the contributions of protein-protein and sRNA-protein mediated interactions for formation of the ternary complex *in vivo*. Unfortunately, construction of the strain is not straight-forward. In our hands, the BACTH reporter strain BTH101 is not amenable to chromosomal manipulations by using engineered recombination tools such as the phage lambda-derived Red system. This may be explained by regulatory elements used by the λ Red system that depend on cAMP, which cannot be synthesized in this strain.

Point b4: We have addressed this query in the response to point b1.

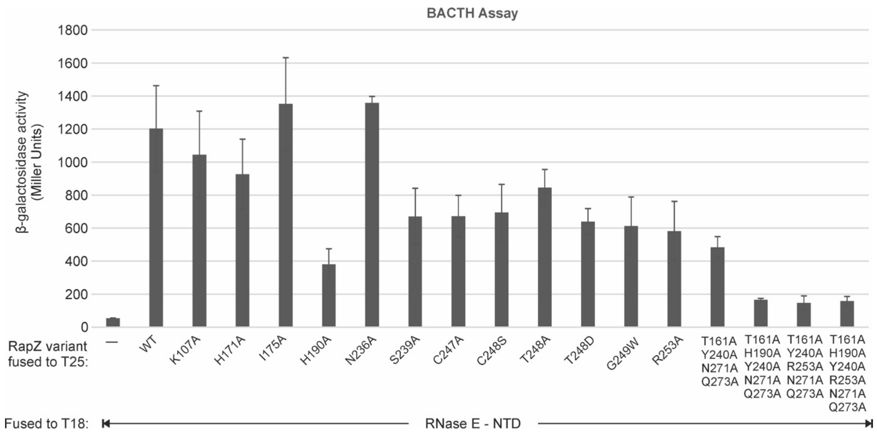

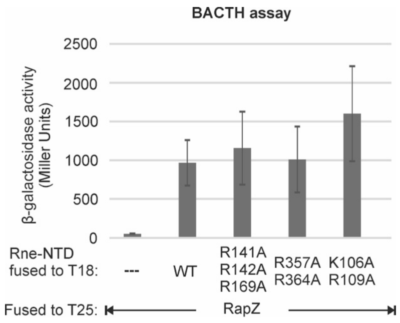

- Should the authors qualify some of their claims as preliminary or speculative, or remove them altogether?
Possibly the importance of RNAse E-RapZ direct interaction, without further proof that this actually is needed for function.

Our reply: We partially addressed this issue already in our response to point b1. Additionally, we also tested activity of the RapZ-4 subst variant that lacks the residues making direct contact to RNase E in our structure (Fig. 3b-d, last two lanes/columns). The results that are now described in the last paragraph of the results section show that this variant retains regulatory activity. Interestingly, the level of processed GlmZ* is strongly reduced in this case, similar to what is observed with the RapZ-S239A and RapZ-T248A variants discussed above. Therefore, these direct protein-protein contacts might have a role for GlmZ* decay in a manner that remains to be addressed.

- Would additional experiments be essential to support the claims of the paper? Request additional experiments only where necessary for the paper as it is, and do not ask authors to open new lines of experimentation.

As noted above, further tests of RapZ mutants for RNA binding would be useful; if this has been done previously, needs to be presented.

Our reply.

This has been addressed in the response above.

Are there Rnase E residues that would be predicted by the model to be critical for the RapZ or GlmZ interaction but are not otherwise needed for activity? Would these disrupt either the BACTH results or activity in vivo?

Our reply.

The RNase E residues that contact RapZ were mutated but had little impact on the BACTH signal, as mentioned above.

- Are the suggested experiments realistic in terms of time and resources? It would help if you could add an estimated cost and time investment for substantial experiments.
Yes, they are. They are generally extrapolations from what is already in the paper or in previous studies by these groups.
- Are the data and the methods presented in such a way that they can be reproduced?
Yes.
- Are the experiments adequately replicated and statistical analysis adequate?
Yes.

Minor comments:
- Specific experimental issues that are easily addressable.
None noted.
- Are prior studies referenced appropriately?
Yes, they are. However, the paper could more clearly outline what is already known at the level of interactions of the molecules under study here.

Our reply. We have changed the text to better introduce information from previous studies: interprotomer contacts, properties of the isolated RapZ domains, conclusions from the truncation analyses, requirements for interaction for RNase E and for sRNA-binding, stabilization of processed GlmZ through RapZ binding (Göpel et al., 2013; Gonzalez et al 2017; Durica-Mitic and Görke, 2019; Durica-Mitic et al., 2020).

- Are the text and figures clear and accurate?
1. In a number of places, the text and figure order/numbers are not correct. See Fig. S1 (p. 4), S2 (legends vs. figure panels).

Our reply. We have corrected these in the revised text.

2. Better labeling in many figures is needed. Clarify what is shown in Fig. S2d, and make the labels readable. Label the particle types in S3. Use schematics more (as in Fig. 4 and S8) to make it easier for reader to follow structure (for Fig. 2, for instance). It is very difficult to

discern RapZ tetramer here. Fig. 3a, it is very difficult to see the residue numbers on the structures. Clearly identify the fructokinase-like domains. Label lanes in Fig. 3b, c, d. Indicate active site for RNase E. in Fig. 4, in schematic at least.

Our reply. We have also corrected these in the revised text.

- Do you have suggestions that would help the authors improve the presentation of their data and conclusions?
Overall, clarify and highlight better how the structures here fit with what is already known about important sequences/regions of RapZ, GlmZ, and Rnase E, maybe color-coding parts of GlmZ shown to be important for RapZ recognition, etc.

Our reply. We have added a sequence alignment for RapZ in the supplementary materials section, indicating important residues (Fig. S12).

Page 12, the second last row. Text after 'In this model...' can be simplified or removed because it is just a hypothesis.

Our reply. We have simplified the text.

Our reply:

We believe that the discussion section should also give room for novel ideas and hypotheses. Therefore, we wish to keep the paragraph.

Reviewer #1 (Significance (Required)):

- Describe the nature and significance of the advance (e.g. conceptual, technical, clinical) for the field.
Rnase E is a major essential endonuclease in bacteria such as E. coli. How accessory proteins lead to its recognition and cleavage of regulatory RNAs such as GlmZ is not well understood at the structural level, and these structures provide important insight into that process. In addition, the GlmZ/RapZ regulatory circuit plays an important role in bacterial growth and pathogenesis, and understanding it at this level of detail will certainly open up possibilities for targeting this process in the future.

- Place the work in the context of the existing literature (provide references, where appropriate).
The components that go into the current structures have been studied previously, with publications on RapZ structure, analysis of critical regions within the GlmZ RNA, and demonstration of the domain of Rnase E involved in interactions with RapZ (Durica-Mitic et al, 2020; Khan et al, 2020, Gonzalez et al, 2017, among others), exactly how these fit together has not been known. Other RNA binding proteins that affect degradation have been reported, but are not fully understood, and ways in which the ribonuclease binds complex RNAs is not fully understood either.
- State what audience might be interested in and influenced by the reported findings.
This work should be of broad interested to the field of RNA-based regulation and RNA degradation, with particular interest for those working on these processes in bacteria.

- Define your field of expertise with a few keywords to help the authors contextualize your point of view. Indicate if there are any parts of the paper that you do not have sufficient expertise to evaluate.
Our expertise is in RNA-based regulation and microbial genetics; we are not able to critically evaluate the cryoEM analysis itself.

Reviewer #2 (Evidence, reproducibility and clarity (Required)):

Summary:

Islam et al present their characterization of the E. coli RapZ-GlmZ-RNase E ternary complex in this manuscript under review. In E. coli, the RNA binding protein RapZ facilitates cleavage of GlmZ sRNA by RNase E when intracellular concentrations of GlcN-6P are high; when GlcN-6P levels are low RapZ is titrated by GlmY sRNA and GlmZ sRNA promotes an increase in the translation and stability of the mRNA encoding GlcN-6P synthase, GlmS. Via Cryo-EM, the authors of this manuscript solve the structure of the binary RapZ:GlmZ (Fig. 2) and ternary RapZ:GlmZ:RNase Y (Fig. 4) complexes. Based on the apparent RapZ-sRNA binding sites in the solved structure of the binary complex, the authors make substitutions in residues suspected to be involved in RNA binding and measure the impact of these substitutions on cleavage of GlmZ and GlmZ-mediated activation of GlmS expression (Fig. 3). The authors find that some of the residues predicted to be involved in RNA binding based on their structural studies are also important for the cleavage of GlmZ, presumably by RNase E. Finally, the authors show via bacterial two-hybrid assays that some residues of RapZ necessary for GlmZ cleavage are also important for its interaction with RNase E (Fig. S11). I would suggest that the authors measure co-immunoprecipitation of GlmZ with tagged-RapZ with or without substitutions in the proposed RNA binding residues to resolve this issue. Alternatively, EMSAs could be performed.

Our reply. Please see the response above to reviewer 1. We have included results from EMSAs with selected RapZ mutants and for multiple mutations in the BACTH analysis.

Major comments:

Overall, the structural studies our impressive and provide considerable insight into the recognition of substrates by RapZ and RNase E. Given the dearth of solved structures of RNAs with their cognate RNA binding proteins, these results are very significant.

A limitation in this work is the lack of experiments directly testing whether or not the residues of RapZ that appear to be important for its interaction with the GlmZ sRNA in the authors' Cryo-EM structures actually have a significant role in RNA binding. In lieu of measuring GlmZ binding by RapZ, the authors measure GlmZ cleavage in strains expressing RapZ or particular variants harboring substitutions in residues that appear to play a role in sRNA binding (Fig. 3b); however, it is impossible for the authors to determine whether impairment of GlmZ cleavage by RNase E in their assays is due to lack of GlmZ binding to RapZ, extraordinarily tight binding of GlmZ to RapZ, changes in the orientation of GlmZ bound to RapZ, or conformational changes in RapZ that lead to disruption of direct RapZ-RNase E contacts. The lack of this empirical data supporting their structural studies becomes

more salient as the authors attempt to test whether RapZ binding of GlmZ is important for its interaction with RNase E via a bacterial two-hybrid assay. Since the authors have not directly examined the importance of particular RapZ residues on GlmZ binding, the authors' interpretation of their results from these assays is very speculative.

Our reply: Reviewer 1 raised a similar point to which we replied above. The role of candidate residues in RapZ for binding GlmZ has been addressed by more direct assays (Pull-down/EMSA).

The authors state on page 7 that "the interaction of RapZ:GlmZ with RNase E does not involve conformational rearrangement of either RapZ or GlmZ". However, the arrangement of SLII relative to SLI appears different between the RapZ:GlmZ and RapZ:GlmZ:RNase E structures presented. Additionally, SLII appears entirely bound by RapZ in the binary complex (Fig. 2b), whereas in the structure of the ternary complex, SLII appears less associated with RapZ (Fig. S4b). A supplementary figure showing side-by-side the structure of GlmZ bound to RapZ solved in the presence or absence of RNase E may make clear whether any differences that exist in the conformation of RapZ and GlmZ between the binary and ternary complex structures.

Our reply: In the revised manuscript, we have included a supplementary figure showing side-by-side comparisons of the structures.

Minor comments:
Figure S1 legend. Change "inactivate" to "inactive" or "inactivated"

Figure S2 legend. The description for "(d)" is for S2c and the text for "(c)" refers to the image in S2d.

Figure legend S5a and S9a. If resolution in the key is in angstroms, then it should be indicated.

Our reply: We have now corrected the above points in the revised text.

Figure 1. The model appears to indicate that the apo-form of RapZ binds GlmZ and GlmY, whereas the GlcN-6P bound form does not. Moreover, in the discussion, the authors indicate that GlcN-6P interferes with GlmZ binding to RapZ. How does RapZ bind and cleave GlmZ when GlcN-6P levels are high, if GlcN-6P interferes with GlmZ binding? It would be useful for the authors to address this conundrum in their discussion.

Our reply. We thank the reviewer for pointing out this paradox. Our unpublished work indicates that RapZ may have phosphatase activity for GlcN6P, and we added a comment to this in the discussion section.

Fig. S3B and C. While panels in Fig. S3B and C seemed well aligned, numbering of lanes would provide additional clarity.

We will provide lane numbers, accordingly.

Many bacterial species including Bacillus subtilis, Streptococcus pyogenes, and Clostridium botulinum have RapZ homologs that bear a tyrosine instead of a histidine residue at the position corresponding to H190 in E. coli RapZ. Would you expect this change to reduce GlmZ binding by RapZ or lead to change in RNA specificity based on your structural data? This may be useful to discuss in the manuscript.

We believe that the is more behind this question. Likely, the referee (by inspecting a RapZ sequence alignment) realized that almost all residues proposed to be involved in binding GlmZ are also conserved in RapZ homologs in Gram-positive bacteria, unless His190 and His171, which are replaced by tyrosines in some of these species. However, no RNA-binding activity has been reported for the Gram-positive RapZ homologs. If true, the question arises what is making the difference here? In principle, this could be due to the lacking histidine residues, which are replaced by tyrosines in Gram-positive RapZs. Alternatively, we consider that the positively charged residues at the far C-terminus (K270, K281, R282, K283), which were identified previously to be required for sRNA binding (Göpel et al., 2013; Durica-Mitic et al., 2020), and which could not be resolved in the current structures, are additionally required to obtain RNA-binding activity.

Fig. S10. It is confusing to me that the yellow chain in the structure of RNase E is labeled as the DNase I-domain in the apo structure, whereas in the structure with RprA or GlmZ bound, this colored region is labeled as the 5' sensing domain.

We have changed the figure to make it clearer.

On page 12, the authors appear to indicate that their structural studies of the RapZ-GlmZ-RNase E ternary complex could be informative with regards to how KH domain proteins in Gram-positive bacteria could present their substrates to RNase E. First of all, these bacteria lack RNase E and instead encode an evolutionarily distinct endoribonuclease (RNase Y). Secondly, I think that it is overreaching to state that these structural studies will inform us on how KH domain proteins such as KhpA/KhpB, which may or may not have a chaperoning function akin to Hfq in Gram-positive bacteria, present substrates to RNase Y. Regardless, if this statement is to remain, the authors should make clear that is RNase Y and not RNase E that they are referring to.

We have changed the text to make clear that a different RNase is employed in this case.

Reviewer #2 (Significance (Required)):

In my opinion, the significance of this work is in the achievement of high-resolution structures of the complexes of the RNA binding protein RapZ and the endoribonuclease RNase Y with RNA substrate bound. There are very few structures solved of RNA binding proteins or RNases with their cognate substrates. This is likely due to the difficult in obtaining

high resolution data for the bound RNA that may have a large degree of flexibility or many alternative conformations. More structures like this are needed to advance our understanding of RNA-protein interactions.

I believe that these findings would not only be of great interest to those that study small regulatory RNAs, such as myself, but also others more generally interested in RNA binding proteins, RNases, or protein-RNA interactions.

Field of expertise: small regulatory RNAs, RNA chaperones, RNases

Referees cross-commenting

1. I agree with Reviewer #1 that the results of the bacterial two-hybrid assay would be more informative, if the authors tested the impact of deletion of glmZ on the ability of the wild type and mutant RapZ proteins to interact with RNase Y by this assay.

2. As both reviewer #1 and I indicated, I think that it would be useful for the authors to directly assess the effect of key substitutions in RapZ on GlmY binding by a more direct measure of interaction, e.g., CoIP or EMSA.

3. I do think that it would be nice at some point for the authors to actually provide evidence that GlcN6P binds to the site that they predict as reviewer 3 suggested but this may be be beyond the scope of this manuscript and may be better addressed in another manuscript in which the authors solve the structure of RapZ with GlcN6P bound. In the meantime, the authors could limit their speculation.

Reviewer #3 (Evidence, reproducibility and clarity (Required)):

Summary: The biogenesis of the bacterial cell envelope relies on glucosamine-6-phosphate (GlcN6P), which is mediated by GlmZ and the sRNA-binding protein RapZ. GlmZ stimulates translation of the GlcN6P synthetase. When the levels of the GlcN6P are sufficiently high, RapZ will presents GlmZ to the endoribonuclease RNase E for cleavage and thereby silencing synthesis of the GlcN6P synthetase. However, how RapZ recruit RNase E to GlmZ for degradation is still unsolved. This paper reports the cryoEM structure of the binary complex of RapZ: GlmZ and the ternary complex of the RNase E catalytic domain (RNase E-NTD), RapZ and GlmZ. RapZ interacts with SLI and SLII of GlmZ through complementarity in shape and electrostatic charge to the phosphodiester backbone of the sRNA and presents the sRNA by alignning its SSR comprising the cleavage site into the RNase E active center. This paper suggests a general RNase E recognition pathway for complex substrates, which will help to understand the mechanisms that other RNA chaperones such as Hfq might work in an analogous assembly to present base-paired sRNA/mRNA pairs for cleavage. In total, this is an excellent work. I will support the publication of it until these following points are presented.

Major comments:
1. It was mentioned on Page 5 that "Sulphate and malonate ions were previously seen at these positions in crystal structures of apo RapZ" and pn Page 11 that " Interestingly, the phosphate groups of the RNA backbone occupy positions in RapZ that were previously observed to bind sulphate or malonate ions in the crystal structure of apo-RapZ, suggesting that this pocket could be the binding site for a charged metabolite such as GlcN6P". Is there any following experiments to investigate it further? If possible, I suggest the author to confirm that weather RapZ has the binding activity with GlcN6P or not.

Binding of GlcN6P by the RapZ-CTD was demonstrated previously by SPR as well as by metabolomics of metabolites copurifying with RapZ (Khan et al., 2020), although evidence that the "sulphate/malonate binding sites" in RapZ also bind GlcN6P is still lacking. Crystallization of RapZ+GlcN6P is not straight forward as bound GlcN6P is apparently hydrolyzed over time.

2. "The kinase-like N-terminal domain of RapZ (NTD) makes only a few interactions with the RNA, and the path of the RNA does not encounter the Walker A or B motifs (Figure 2b). It is possible that this domain could act as an allosteric switch, whereby the binding of an as yet unknown ligand triggers quaternary structural changes that affect RapZ functions." Is there any more structural information supporting it? If the domain act as an allosteric switch, is it possible to make some deletion or substitution to test it?

The properties of the separated NTD and CTD of RapZ were assessed in previous work.

3. Is there any results to compare the binding affinity of GlmY and GlmZ with RapZ?

Affinities were determined previously using complimentary techniques:
Göpel et al., 2013/EMSA: K_D GlmY ~ 30 nM; K_D GlmZ ~ 75 nM
Gonzalez et al., 2017/biolayer interferometry: ~ 50 nM for both GlmY/GlmZ (full-length)

Minor comments:
1. Page 8, is it "stabilised" or "stabilized", please check it.

We have changed the spelling to "stabilized".

2. The legends for Figure S2 c and d are reversed.

This has now been corrected.

3. It was suggested to show the RNA molecules in Figure S1a.

We have changed the figure to include single-stranded RNA substrate.

Reviewer #3 (Significance (Required)):

This paper suggests a general RNase E recognition pathway for complex substrates, which

will help to understand the mechanisms that other RNA chaperones such as Hfq might work in an analogous assembly to present base-paired sRNA/mRNA pairs for cleavage.
In total, this is an excellent work.

1st Editorial Decision **3rd Oct 2022**

Dear Prof. Görke,

Congratulations on a great revision! Overall, the referees have been positive and support publication.

However, there remain several editorial items to address in a revised version, and please also add these to your final point-by-point response:

1. Please upload the author checklist (see attachment)

2. Please upload the main figures as individual, high-resolution figure files and remove them from the manuscript text, but the figure legends should stay in the manuscript text. Up to five EV figures (from the supplemental file) can also be uploaded as individual figure files and their legends should then be added to the manuscript, after the main figure legends.

3. Please limit the number of keywords to 5.

4. Please include a Data Availability statement according to the online EMBO author guide.

5. Please enter author contributions into EJP and use the free text boxes to describe any specific contributions.

6. Please review our new policy on conflict of interests on the EMBO author guide website and update the title of this section to: Disclosure and competing interests statement.

7. Please correct the format of references as per the author guidelines: In the reference list, citations should be listed in alphabetical order and then chronologically, with the authors' surnames and initials inverted; where there are more than 10 authors, 10 will be listed, followed by et al.

8. Please rename the appendix file to "Appendix" and the nomenclature to "Appendix Table S" etc. and "Appendix Figure S1" etc. in the appendix file and callouts in the manuscript. Please also add a table of contents with page numbers and remove the yellow highlights.

9. We encourage the publication of source data, particularly for electrophoretic gels and blots and graphs, with the aim of making primary data more accessible and transparent to the reader. It would be great if you could provide me with a PDF file per figure that contains the original, uncropped, and unprocessed scans of all or key gels used in the figures or for graphs, an Excel spreadsheet with the original data used to generate the graphs. The PDF files should be labeled with the appropriate figure/panel number and should have molecular weight markers; further annotation could be useful but is not essential. The PFD files will be published online with the article as supplementary "Source Data" files.

10. We include a synopsis of the paper (see http://emboj.embopress.org/). Please provide me with a general summary statement and 3-5 bullet points that capture the key findings of the paper.

11. We also need a summary figure for the synopsis. The size should be 550 wide by 200-440 high (pixels). You can also use something from the figures if that is easier.

12. We do not allow references to data that is not shown. Please removed "data not shown" from p.11 and p.13.

13. We require that all figures be referred to in the manuscript text and in their same order as the figures. Please include a figure callout for Fig. 2c, Fig. 4a, and Fig. 5b in the manuscript text.

14. Please upload the manuscript in .docx format rather than as a PDF.

Thank you for the opportunity to consider your work for publication. I look forward to your revision.

Yours sincerely,

Kelly M Anderson, PhD
Editor
The EMBO Journal
k.anderson@embojournal.org

Referee #1:

Summary:
In the revised manuscript submitted to EMBO Journal, Islam et al present their findings on the interactions that occur within the Escherichia coli RapZ-GlmZ-RNase E ternary complex as the sRNA substrate is delivered to this endoribonuclease for degradation. In E. coli, the RNA binding protein RapZ facilitates cleavage of GlmZ sRNA by RNase E when intracellular concentrations of GlcN-6P are high; when GlcN-6P levels are low RapZ is titrated by GlmY sRNA and GlmZ sRNA promotes an increase in the translation and stability of the mRNA encoding GlcN-6P synthase, GlmS. Via Cryo-EM, the authors of this manuscript solve the structure of the binary RapZ:GlmZ (Fig. 2) and ternary RapZ:GlmZ:RNase Y (Fig. 4) complexes. Based on the apparent RapZ-sRNA binding sites in the solved structure of the binary complex, the authors make substitutions in residues suspected to be involved in RNA binding and measure the impact of these substitutions on cleavage of GlmZ and GlmZ-mediated activation of GlmS expression (Fig. 3). For substitutions in key residues that disrupt GlmZ processing and function, the authors then evaluate the effects of those changes on GlmZ binding (Fig. S7c) and demonstrate a key role for residues K170, H190, C247, T248, G249, and R253 of RapZ in GlmZ binding and processing. Additionally, the authors provide evidence for direct interactions between RNase E and RapZ via residues Q273, N271, Y240, and T161 (Figs 4 and 7). Overall, the authors provide a detailed, impressive molecular and structural characterization of the interactions that occur between RapZ, GlmZ, and RNase E as RapZ presents its RNA cargo to this endonuclease for cleavage.

Significance:
In my opinion, the significance of this work is in the achievement of high-resolution structures of the complexes of the RNA binding protein RapZ and the endoribonuclease RNase Y with RNA substrate bound. There are very few structures solved of RNA binding proteins or RNases with their cognate substrates. This is likely due to the difficult in obtaining high resolution data for the bound RNA that may have a large degree of flexibility or many alternative conformations. More structures like this are needed to advance our understanding of RNA-protein interactions. I believe that these findings would not only be of great interest to those that study small regulatory RNAs, such as myself, but also others more generally interested in RNA binding proteins, RNases, or protein-RNA interactions.

Major comments:

None.

Minor comments:

None.

Referee #2:

As previously discussed, this is an important set of structures that provide new insight into how the RapZ protein presents GlmZ to RNase E. The revised version adds useful new information as well as clearer explanation of how this complicated pathway works.
Minor corrections or suggestions:

1) In order to make a BTH101 mutant derivative, P1 transduction from another strain can be carried out, growing the culture in cyclic AMP to suppress the growth defects of the BTH101 strain. This is not essential here, but if the glm deletion changed the outcome of the BACTH results, it would be striking.
2) Fig. S2A: labels say eution, not elution.

Referee #3:

All my questions have been addressed satisfactorily. I support publish of the paper.

The authors addressed the minor editorial issues.

1st Revision - Editorial Decision 25th Oct 2022

Dear Ben,

Thank you for addressing the majority of our editorial concerns. There do however remain just a couple of editorial issues to attend to before we can continue to move forward with your manuscript. Please address the following:

1. The summary figure for the synopsis needs resizing (550 wide by 200-400 high pixels). Please also change the font because we believe it will be difficult to read when the correct dimensions are used. If you are usure of how to resize, we can do this on our end, but please change the font.

2. We are still unable to find callouts for Fig. 2c. Please add a reference to this figure in the main manuscript text.

Thank you for the opportunity to consider your work for publication. I look forward to moving forward with your manuscript when these issues are complete.

Kind regards,

Kelly

Kelly M Anderson, PhD
Editor
The EMBO Journal
k.anderson@embojournal.org

The authors addressed the minor editorial issues.

2nd Revision - Editorial Decision **28th Oct 2022**

Dear Ben,

Congratulations on an excellent manuscript, I am pleased to inform you that your manuscript has been accepted for publication in the EMBO Journal. Thank you for attending to the final editorial changes. It has been a pleasure to work with you to get this to the acceptance stage.

I will begin the final checks on your manuscript before submitting to the publisher next week. Once at the publisher, it will take about 3 weeks for your manuscript to be published online. As a reminder, the entire review process, including referee concerns and your point-by-point response, will be available to readers.

I will be in touch throughout the final editorial process until publication. In the meantime, I hope you find time to celebrate!

Kind regards,
Kelly

Kelly M Anderson, PhD
Editor
The EMBO Journal
k.anderson@embojournal.org

EMBO Press Author Checklist

Corresponding Author Name: Ben Luisi	
Journal Submitted to: EMBO J	
Manuscript Number: EMBOJ-2022-111129R	

USEFUL LINKS FOR COMPLETING THIS FORM
The EMBO Journal - Author Guidelines
EMBO Reports - Author Guidelines
Molecular Systems Biology - Author Guidelines
EMBO Molecular Medicine - Author Guidelines

Reporting Checklist for Life Science Articles (updated January 2022)
This checklist is adapted from Materials Design Analysis Reporting (MDAR) Checklist for Authors. MDAR establishes a minimum set of requirements in transparent reporting in the life sciences (see Statement of Task: 10.31222/osf.io/9sm4x). Please follow the journal's guidelines in preparing your manuscript.
Please note that a copy of this checklist will be published alongside your article.

Abridged guidelines for figures
1. Data
The data shown in figures should satisfy the following conditions:
- the data were obtained and processed according to the field's best practice and are presented to reflect the results of the experiments in an accurate and unbiased manner.
- ideally, figure panels should include only measurements that are directly comparable to each other and obtained with the same assay.
- plots include clearly labeled error bars for independent experiments and sample sizes. Unless justified, error bars should not be shown for technical replicates.
- if n<5, the individual data points from each experiment should be plotted. Any statistical test employed should be justified.
- Source Data should be included to report the data underlying figures according to the guidelines set out in the authorship guidelines on Data Presentation.

2. Captions
Each figure caption should contain the following information, for each panel where they are relevant:
- a specification of the experimental system investigated (eg cell line, species name).
- the assay(s) and method(s) used to carry out the reported observations and measurements.
- an explicit mention of the biological and chemical entity(ies) that are being measured.
- an explicit mention of the biological and chemical entity(ies) that are altered/varied/perturbed in a controlled manner.
- the exact sample size (n) for each experimental group/condition, given as a number, not a range;
- a description of the sample collection allowing the reader to understand whether the samples represent technical or biological replicates (including how many animals, litters, cultures, etc.).
- a statement of how many times the experiment shown was independently replicated in the laboratory.
- definitions of statistical methods and measures:
  - common tests, such as t-test (please specify whether paired vs. unpaired), simple χ2 tests, Wilcoxon and Mann-Whitney tests, can be unambiguously identified by name only, but more complex techniques should be described in the methods section;
  - are tests one-sided or two-sided?
  - are there adjustments for multiple comparisons?
  - exact statistical test results, e.g., P values = x but not P values < x;
  - definition of 'center values' as median or average;
  - definition of error bars as s.d. or s.e.m.

Please complete ALL of the questions below.
Select "Not Applicable" only when the requested information is not relevant for your study.

Materials

Newly Created Materials	Information included in the manuscript?	In which section is the information available? (Reagents and Tools Table, Materials and Methods, Figures, Data Availability Section)
New materials and reagents need to be available; do any restrictions apply?	Not Applicable	

Antibodies	Information included in the manuscript?	In which section is the information available? (Reagents and Tools Table, Materials and Methods, Figures, Data Availability Section)
For **antibodies** provide the following information:<br>- Commercial antibodies: RRID (if possible) or supplier name, catalogue number and or/clone number<br>- Non-commercial: RRID or citation	Not Applicable	

DNA and RNA sequences	Information included in the manuscript?	In which section is the information available? (Reagents and Tools Table, Materials and Methods, Figures, Data Availability Section)
**Short novel DNA or RNA including primers, probes**: provide the sequences.	Yes	Materials and Methods

Cell materials	Information included in the manuscript?	In which section is the information available? (Reagents and Tools Table, Materials and Methods, Figures, Data Availability Section)
**Cell lines:** Provide species information, strain. Provide accession number in repository **OR** supplier name, catalog number, clone number, and/**OR** RRID.	Not Applicable	
**Primary cultures:** Provide species, strain, sex of origin, genetic modification status.	Not Applicable	
Report if the cell lines were recently **authenticated** (e.g., by STR profiling) and tested for mycoplasma contamination.	Not Applicable	

Experimental animals	Information included in the manuscript?	In which section is the information available? (Reagents and Tools Table, Materials and Methods, Figures, Data Availability Section)
**Laboratory animals or Model organisms:** Provide species, strain, sex, age, genetic modification status. Provide accession number in repository **OR** supplier name, catalog number, clone number, **OR** RRID.	Not Applicable	
**Animal observed in or captured from the field:** Provide species, sex, and age where possible.	Not Applicable	
Please detail **housing and husbandry conditions**.	Not Applicable	

Plants and microbes	Information included in the manuscript?	In which section is the information available? (Reagents and Tools Table, Materials and Methods, Figures, Data Availability Section)
**Plants:** provide species and strain, ecotype and cultivar where relevant, unique accession number if available, and source (including location for collected wild specimens).	Not Applicable	
**Microbes:** provide species and strain, unique accession number if available, and source.	Yes	Materials and Methods

Human research participants	Information included in the manuscript?	In which section is the information available? (Reagents and Tools Table, Materials and Methods, Figures, Data Availability Section)
If collected and within the bounds of privacy constraints report on age, sex and gender or ethnicity for all study participants.	Not Applicable	

Core facilities	Information included in the manuscript?	In which section is the information available? (Reagents and Tools Table, Materials and Methods, Figures, Data Availability Section)
If your work benefited from core facilities, was their service mentioned in the acknowledgments section?	Yes	ACKNOWLEDGEMENT

Design

**Study protocol**	**Information included in the manuscript?**	**In which section is the information available?** (Reagents and Tools Table, Materials and Methods, Figures, Data Availability Section)
If study protocol has been **pre-registered, provide DOI in the manuscript**. For clinical trials, provide the trial registration number **OR** cite DOI.	Not Applicable	
Report the **clinical trial registration number** (at ClinicalTrials.gov or equivalent), where applicable.	Not Applicable	

**Laboratory protocol**	**Information included in the manuscript?**	**In which section is the information available?** (Reagents and Tools Table, Materials and Methods, Figures, Data Availability Section)
Provide DOI OR other citation details if **external detailed step-by-step protocols** are available.	Not Applicable	

**Experimental study design and statistics**	**Information included in the manuscript?**	**In which section is the information available?** (Reagents and Tools Table, Materials and Methods, Figures, Data Availability Section)
Include a statement about **sample size** estimate even if no statistical methods were used.	Not Applicable	
Were any steps taken to minimize the effects of subjective bias when allocating animals/samples to treatment (e.g. **randomization procedure**)? If yes, have they been described?	Not Applicable	
Include a statement about **blinding** even if no blinding was done.	Not Applicable	
Describe **inclusion/exclusion criteria** if samples or animals were excluded from the analysis. Were the criteria pre-established?<br><br>If sample or data points were omitted from analysis, report if this was due to attrition or intentional exclusion and provide justification.	Not Applicable	
For every figure, are **statistical tests** justified as appropriate? Do the data meet the assumptions of the tests (e.g., normal distribution)? Describe any methods used to assess it. Is there an estimate of variation within each group of data? Is the variance similar between the groups that are being statistically compared?	Not Applicable	

**Sample definition and in-laboratory replication**	**Information included in the manuscript?**	**In which section is the information available?** (Reagents and Tools Table, Materials and Methods, Figures, Data Availability Section)
In the figure legends: state number of times the experiment was **replicated** in laboratory.	Yes	Figures
In the figure legends: define whether data describe **technical or biological replicates**.	Yes	Figures

Ethics

**Ethics**	**Information included in the manuscript?**	**In which section is the information available?** (Reagents and Tools Table, Materials and Methods, Figures, Data Availability Section)
Studies involving **human participants**: State details of **authority granting ethics approval** (IRB or equivalent committee(s), provide reference number for approval.	Not Applicable	
Studies involving **human participants**: Include a statement confirming that **informed consent** was obtained from all subjects and that the experiments conformed to the principles set out in the WMA Declaration of Helsinki and the Department of Health and Human Services Belmont Report.	Not Applicable	
Studies involving **human participants:** For publication of **patient photos**, include a statement confirming that consent to publish was obtained.	Not Applicable	
Studies involving experimental **animals**: State details of **authority granting ethics approva**l (IRB or equivalent committee(s), provide reference number for approval. Include a statement of compliance with ethical regulations.	Not Applicable	
Studies involving **specimen and field samples:** State if relevant **permits** obtained, provide details of authority approving study; if none were required, explain why.	Not Applicable	

**Dual Use Research of Concern (DURC)**	**Information included in the manuscript?**	**In which section is the information available?** (Reagents and Tools Table, Materials and Methods, Figures, Data Availability Section)
Could your study fall under dual use research restrictions? Please check biosecurity documents and list of **select agents and toxins** (CDC): https://www.selectagents.gov/sat/list.htm	Not Applicable	
If you used a select agent, is the security level of the lab appropriate and reported in the manuscript?	Not Applicable	
If a study is subject to dual use research of concern regulations, is the name of the **authority granting approval and reference number** for the regulatory approval provided in the manuscript?	Not Applicable	

Reporting

The MDAR framework recommends adoption of discipline-specific guidelines, established and endorsed through community initiatives. Journals have their own policy about requiring specific guidelines and recommendations to complement MDAR.

**Adherence to community standards**	**Information included in the manuscript?**	**In which section is the information available?** (Reagents and Tools Table, Materials and Methods, Figures, Data Availability Section)
State if relevant guidelines or checklists (e.g., **ICMJE, MIBBI, ARRIVE, PRISMA**) have been followed or provided.	Not Applicable	
For **tumor marker prognostic studies,** we recommend that you follow the **REMARK** reporting guidelines (see link list at top right). See author guidelines, under 'Reporting Guidelines'. Please confirm you have followed these guidelines.	Not Applicable	
For **phase II and III randomized controlled trials**, please refer to the **CONSORT** flow diagram (see link list at top right) and submit the CONSORT checklist (see link list at top right) with your submission. See author guidelines, under 'Reporting Guidelines'. Please confirm you have submitted this list.	Not Applicable	

Data Availability

**Data availability**	**Information included in the manuscript?**	**In which section is the information available?** (Reagents and Tools Table, Materials and Methods, Figures, Data Availability Section)
Have **primary datasets** been deposited according to the journal's guidelines (see 'Data Deposition' section) and the respective accession numbers provided in the Data Availability Section?	Yes	Data Availability Section: PDB accession codes 8B0I, 8B0J. EMD accession codes EMD-15784, EMD-15785
Were **human clinical and genomic datasets** deposited in a public access-controlled repository in accordance to ethical obligations to the patients and to the applicable consent agreement?	Not Applicable	
Are **computational models** that are central and integral to a study available without restrictions in a machine-readable form? Were the relevant accession numbers or links provided?	Not Applicable	
If publicly available data were reused, provide the respective **data citations in the reference list**.	Not Applicable	
