# Supplementary material for: Structure of a bacterial ribonucleoprotein complex central to the control of cell envelope biogenesis
Source: EMBO J. 2022 Dec 12;42(2):e112574. doi: 10.15252/embj.2022112574 (PMC9841335; doi:10.15252/embj.2022112574)

Fig. S7c: parts of blots and gels shown are indicated by black panels

Coomassie stained ge

Coomassie stained gel

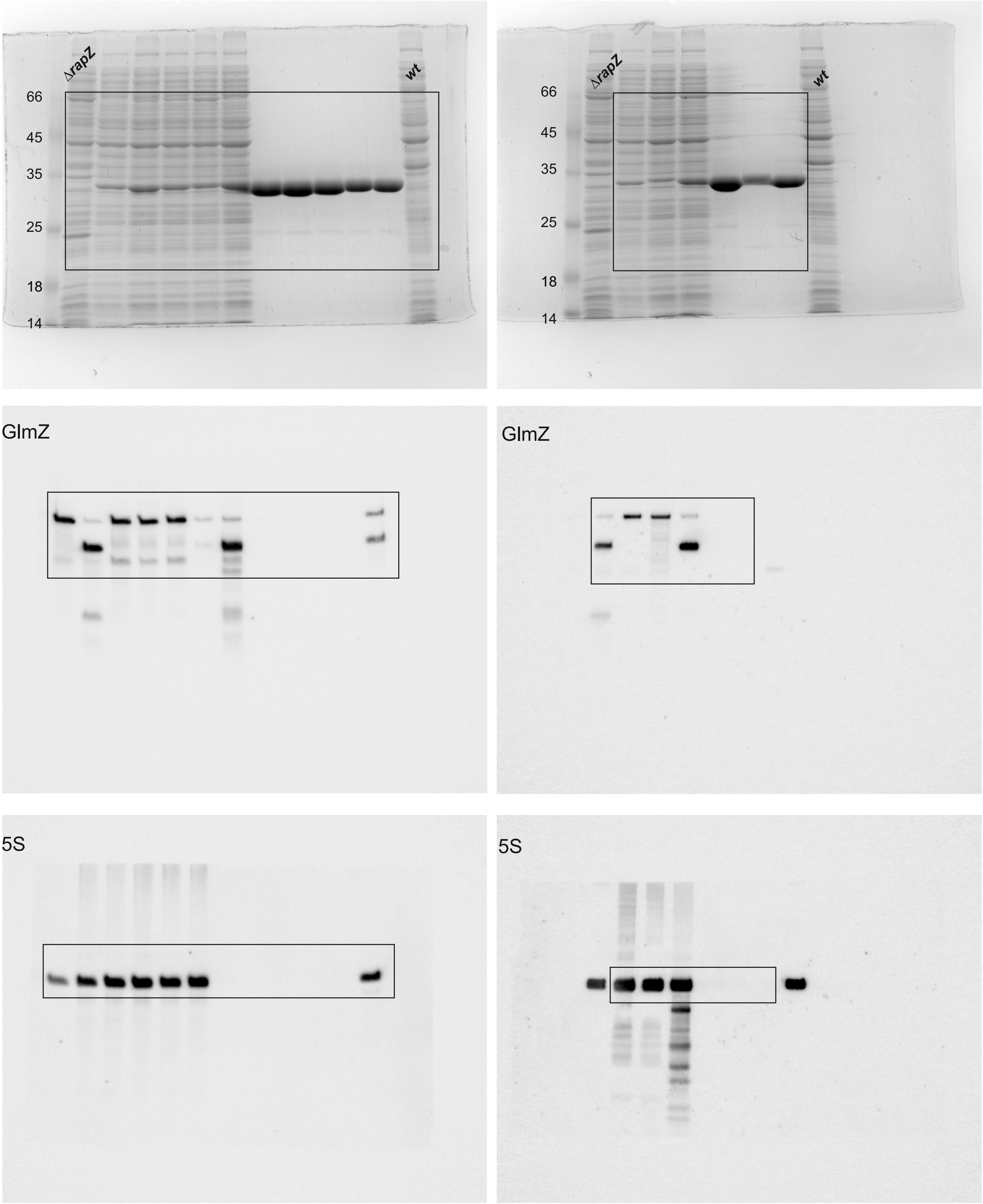

Supplement: Supplementary file 4 — Source Data for Appendix [file EMBJ-42-e112574-s004.zip › EMBJ_2490_SD Appendix Figues.zip!/Fig_S4_raw.pdf]
